# Outcome Measures for Disease-Modifying Trials in Parkinson’s Disease: Consensus Paper by the EJS ACT-PD Multi-Arm Multi-Stage Trial Initiative

**DOI:** 10.3233/JPD-230051

**Published:** 2023-09-08

**Authors:** Cristina Gonzalez-Robles, Rimona S. Weil, Daniel van Wamelen, Michèle Bartlett, Matthew Burnell, Caroline S. Clarke, Michele T. Hu, Brook Huxford, Ashwani Jha, Christian Lambert, Michael Lawton, Georgia Mills, Alastair Noyce, Paola Piccini, Kuhan Pushparatnam, Lynn Rochester, Carroll Siu, Caroline H. Williams-Gray, Marie-Louise Zeissler, Henrik Zetterberg, Camille B. Carroll, Thomas Foltynie, Anette Schrag, on behalf of the EJS ACT-PD Consortium, Roger Barker, James Carpenter, Yoav Ben Shlomo, Mark Edwards, Alan Whone, Carl Counsell, Dorothy Salathiel, Sue Whipps, Anna Jewell, Priti Gros, Tom Barber, Shlomi Haar Millo, K Ray Chaudhuri, Anthony HV Schapira, Oliver Bandmann, Simon Stott, George Tofaris, Esther Sammler, Heather Mortiboys, Li Wei, Alan Wong, Susan Duty, David Dexter, Paula Scurfield, Keith Martin, Edwin Jabbari, Stephen Mullin, Huw Morris, David Breen, Christian Lambert, Prasad Korlipara, Monty Silverdale, Kailash Bhatia, Alison Yarnall, Raj Khengar, Helen Collins, Fleur Hudson, Gareth Baxendale, Rebecca Croucher, Sandra Bartolomeu-Pires, Jennifer Allison, Jodie Forbes, Alex Edwards, Sheila Wonnacott, Dilan Athauda, Joy Duffen, Sonia Gandhi, Emily Henderson, Maryanne Graham, Shona Clegg, Karen Matthews, Vince Greaves, Eric Deeson, Laurel Miller, Joel Handley, David Dexter, Helen Matthews, Kevin McFarthing, Amit Batla, Nikul Bashi, Emma Lane, Miriam Parry, Natasha Ratcliffe, Romy Ellis-Doyle, Sally L Collins, Rebecca Chapman, Jesse Cedarbaum, Anthony Lang, Brian Fiske, Richard Wyse, Mahesh Parmar, Adam Boxer, Denise Wilson, Jean Christophe Corvol, Jennifer Harris

**Affiliations:** a University College London, London, UK; b King’s College London, London, UK; c Expert by experience, Guildford, UK; d Medical Research Council Clinical Trials Unit at University College London, London, UK; e University of Oxford, Oxford, UK; f Queen Mary University of London, London, UK; g University of Bristol, Bristol, UK; h Imperial College London, London, UK; i Expert by experience, London, UK; j Newcastle University, Newcastle, UK; k Expert by experience, Canterbury, UK; l University of Cambridge, Cambridge, UK; m University of Plymouth, Plymouth, UK; n University of Gothenburg, Mölndal, Sweden

**Keywords:** Parkinson’s disease, neuroprotection, outcome measures, biomarkers, clinical trials, consensus

## Abstract

**Background:**

Multi-arm, multi-stage (MAMS) platform trials can accelerate the identification of disease-modifying treatments for Parkinson’s disease (PD) but there is no current consensus on the optimal outcome measures (OM) for this approach.

**Objective:**

To provide an up-to-date inventory of OM for disease-modifying PD trials, and a framework for future selection of OM for such trials.

**Methods:**

As part of the Edmond J Safra Accelerating Clinical Trials in Parkinson Disease (EJS ACT-PD) initiative, an expert group with Patient and Public Involvement and Engagement (PPIE) representatives’ input reviewed and evaluated available evidence on OM for potential use in trials to delay progression of PD. Each OM was ranked based on aspects such as validity, sensitivity to change, participant burden and practicality for a multi-site trial. Review of evidence and expert opinion led to the present inventory.

**Results:**

An extensive inventory of OM was created, divided into: general, motor and non-motor scales, diaries and fluctuation questionnaires, cognitive, disability and health-related quality of life, capability, quantitative motor, wearable and digital, combined, resource use, imaging and wet biomarkers, and milestone-based. A framework for evaluation of OM is presented to update the inventory in the future. PPIE input highlighted the need for OM which reflect their experience of disease progression and are applicable to diverse populations and disease stages.

**Conclusion:**

We present a range of OM, classified according to a transparent framework, to aid selection of OM for disease-modifying PD trials, whilst allowing for inclusion or re-classification of relevant OM as new evidence emerges.

## INTRODUCTION

There is currently no proven intervention to delay the progression of Parkinson’s disease (PD). A number of novel and promising treatment approaches are being developed to address this and need to be tested in clinical trials. Multi-arm, multi-stage (MAMS) platform trials may help accelerate the identification of potentially successful treatments by improving efficiency of the clinical trial process. MAMS trials evaluate multiple agents simultaneously against a shared placebo arm and allow the addition of new arms as well as cessation of ineffective treatments at interim stages. However, there is no current consensus on the most appropriate outcome measures (OM) for disease-modifying trials in PD to be included in such an approach.

The Edmond J Safra Accelerating Clinical Trials in Parkinson Disease (EJS ACT-PD) initiative aims to accelerate the identification of disease-modifying treatments for PD through a MAMS platform trial approach. An important component of this novel approach is the identification and selection of appropriate outcome measures, suitable for inclusion across several different study arms as well as meeting the overarching aim. Here, we present an inventory of outcome measures based on current evidence and make initial recommendations for their potential inclusion as core, supplementary (depending on study arm) or exploratory outcome measures in such trials.

This inventory of potential outcome measures for use in disease-modifying trials is based on a consensus effort by an expert group with strong patient and public engagement input. The group used information from literature reviews, other existing and ongoing efforts, and discussion with regulatory bodies and group discussions. Particular consideration for inclusion in the inventory was given to clinically relevant outcome measures that are meaningful to patients, align with regulatory expectations and provide data to support adoption in larger healthcare systems. For future adaptation according to emerging new evidence, a framework was also created for evaluation and inclusion of outcome measures of potential relevance in the future, including clinical outcome measures, biomarkers, and novel measurement technologies.

## METHODS

**Fig. 1:**
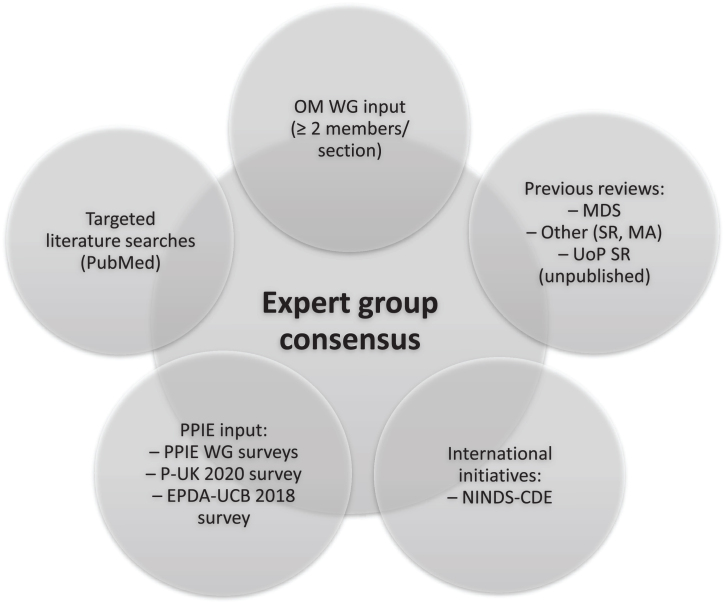
Sources for consensus of outcome measures in disease-modifying trials in Parkinson’s disease. EPDA-UCB, European Parkinson’s Disease Association-UCB Pharma; MA, meta-analysis; MDS, Movement Disorders Society (critique and recommendation papers on different outcome measures); NINDS-CDE, National Institute of Neurological Disorders and Stroke Common Data Elements initiative; OM, Outcome Measures; P-UK, Parkinson’s UK; PPIE, Patient and Public Involvement and Engagement; SR, Systematic review; UoP, University of Plymouth; WG, Working Group.

The methodology for this consensus paper is summarized in Fig. 1. A working group (WG) of experts from relevant fields, chaired by AS, was formed to review evidence, and provide expert input in written form and meetings. An initial list of outcome measures for inclusion in the inventory was compiled, based on 1) expert input from the members of the EJS ACT-PD Outcome Measures Working Group (OM WG), 2) the Movement Disorders Society (MDS) critique and recommendation papers, 3) the National Institute of Neurological Disorders and Stroke Common Data Elements (NINDS-CDE) version 2.0, 4) literature searches performed by CGR, 5) patient and public involvement and engagement (PPIE) input, and 6) a systematic review on disease-modifying trials in PD (Dr. Marie-Louise Zeissler, unpublished). Only outcome measures with published data in PD were included.

From this initial list, at least two members of the OM WG with relevant expertise, and supported by CGR, compiled and reviewed the most relevant PD endpoints in each of the following domains: motor, non-motor, disability, health-related quality of life (HR-QoL), resource use, cognitive, digital, quantitative motor, neuroimaging, and wet biomarkers. Where available, MDS critiques and recommendation papers on different measuring instruments for PD in the categories included in this paper were reviewed (https://www.movementdisorders.org/MDS/MDS-Rating-Scales/Rating-Scales-Critiques-and-Recommendations.htm), and the outcome measures classified as “Recommended” were included for consideration. Targeted literature searches in PubMed were then conducted to identify new measures developed since the publication of the MDS recommendations, or new evidence on measures not previously fulfilling the “Recommended” criteria, with a focus on “Suggested” measures. Other recent reviews were considered when writing this consensus paper. For example, for the disability measures, a 2022 systematic review was used to further guide the choice of outcome measures [1]. Similarly, a 2021 systematic review was employed to aid the decision on health-related quality of life outcome measures [2]. Supplementary Table 1 summarizes the “Recommended” instruments according to the corresponding MDS critique and review papers, the Neurological Disorders and Stroke Common Data Elements (http://www.commondataelements.ninds.nih.gov/) [3] in PD version 2.0 recommendations [4], and our selected outcome measures.

**Table 1:** Selected outcome measures for consideration and level of recommendation

Category	Instrument/Test	NINDS-CDE v2.0 classification	Proposed classification for disease-modifying trials
Global – Generic	CGI-I	Supplemental-HR	Core
Global – PD-specific	MDS-UPDRS (and UPDRS, and remote versions)	Core	Core
	LEDD	NI	Core^*^
Motor – General	MDS-UPDRS Gait-axial score	NI	Supplemental
	Hoehn &Yahr scale	Core	Core
	UDysRS	Supplemental-HR	Supplemental
Gait, balance, and falls	Question about falls (e.g., ProFaNE falls definition)	NI	Core
	Mini-BESTest	NI	Supplemental
	Berg Balance Scale	NI	Supplemental
	FES-I	NI	Supplemental
	ABC Scale	NI	Supplemental
Speech and swallowing	SWAL-QOL	Supplemental	Supplemental
	SDQ	Supplemental	Supplemental
	ROMP	Supplemental	Supplemental
Fluctuations	PD Home Diary (Hauser Diary)	Supplemental-HR	Supplemental
	CAPSIT-PD On/Off Diary	Supplemental-HR	Supplemental
	WOQ-9 and WOQ-19	Supplemental-HR	Supplemental
Non-motor – General	NMSQ	Supplemental	Supplemental
	NMSS	NI	Supplemental
	MDS-NMS	Supplemental-HR	Supplemental
Fatigue	FSS	Supplemental	Supplemental
Pain	KPPS	Supplemental	Supplemental
Sleep	PDSS-2	Supplemental-HR	Supplemental
	ESS	Supplemental-HR	Supplemental
Depression	PHQ-9	Supplemental	Core
	GDS-15	Supplemental-HR	Supplemental
C-SSRS	Supplemental-HR	Supplemental^**^
Apathy	AS	Supplemental-HR	Supplemental
	AES	NI	Supplemental
	LARS	Supplemental-HR	Supplemental
Psychosis	SAPS-PD	Supplemental-HR	Supplemental
	eSAPS-PD	Supplemental-HR	Supplemental
Autonomic dysfunction	SCOPA-AUT	Supplemental	Supplemental
Cognitive measures	MoCA	Core	Core
	DRS-2	Supplemental (MDRS)	Supplemental
	PD-CRS	Supplemental	Supplemental
	ACE-III	Supplemental	Supplemental
	ADAS-Cog	Supplemental	Supplemental
	MMSE	Supplemental	Supplemental
	MMP	NI	Supplemental
	SCOPA-COG	Supplemental	Supplemental
Disability	S&E ADL	Supplemental-HR	Core
	FSQ	NI	Supplemental
Capability	ICECAP	NI	Core
Carer measures	PQoL Carers	NI	Supplemental
	PDQ-Carer	NI	Supplemental
	Zarit Burden Interview	NI	Supplemental
HR-QoL – Generic	EQ-5D-5L	Supplemental-HR (EQ-5D)	Core
	SF-36	Supplemental-HR	Supplemental
	SF-12	NI	Supplemental
	PROMIS/Neuro-QoL	Supplemental-HR (Neuro-QoL)	Supplemental
	HUI	NI	Supplemental
HR-QoL – PD-specific	PDQ-8	NI	Core
	PDQ-39	Supplemental-HR	Supplemental
Resource use	CSRI in combination with EHR	NI	Core
	EHR in combination with CSRI	NI	Core
Milestone-based OM	To be determined (see Supplement)	NI	Exploratory
Digital measures – Active only	OPDC Smartphone app	Exploratory	Exploratory
	CloudUPDRS smartphone-based measures of limb-specific tremor/bradykinesia	Exploratory	Exploratory
	Mobility lab system (APDM)-measures acquired typically in controlled settings	Exploratory	Exploratory
	mPower smartphone-derived composite (dominantly motor) impairment score	Exploratory	Exploratory
Digital measures – Passive only	PKG-based proxy measures of whole-body tremor/bradykinesia/dyskinesia	Exploratory	Exploratory
	MM4D-based proxy measure of whole-body tremor/dyskinesia	Exploratory	Exploratory
	Axivity (AX3 &AX6) gait accelerometer	Exploratory	Exploratory
Digital measures – Active and passive	Roche smartphone app	Exploratory	Exploratory
	Other digital/timed motor measures	Exploratory	Exploratory
Quantitative motor measures	TUG 3 meter	NI	Supplemental
	Purdue Pegboard test	NI	Supplemental
	Alternate tap test	NI	Supplemental
	BRAIN tap test	NI	Supplemental
	9-hole peg test	NI	Supplemental
Composite quantitative motor measures	OPDC composite clinical score	NI	Exploratory
Molecular neuroimaging	Dopaminergic SPECT	NI (PET-SPECT Localization: Supplemental – HR; Supplemental)	Exploratory
	Dopaminergic PET	See above	Exploratory
	Non-dopaminergic SPECT	See above	Exploratory
	Non-dopaminergic PET	See above	Exploratory
	Magnetic Resonance Spectroscopy	Supplemental-HR	Exploratory
Structural neuroimaging	T1 Structural sequence	Supplemental-HR	Exploratory
	Diffusion imaging	NI	Exploratory
	Multiple Parametric Mapping Protocol	NI	Exploratory
	Neuromelanin	NI	Exploratory
	Iron-sensitive sequences	NI	Exploratory
Wet biomarkers	Plasma/serum NfL	NI	Exploratory
	Plasma tau	NI	Exploratory
	Plasma *α*-syn	NI	Exploratory
	CSF NfL	NI	Exploratory
	CSF tau	NI	Exploratory
	CSF *α*-syn	NI	Exploratory
	CSF *α*-syn aggregation	NI	Exploratory
	CSF A*β*	NI	Exploratory
	Salivary markers (e.g., salivary *α*-syn)	NI	Exploratory

^*^Change in LEDD is recommended as Core in trials including PD patients taking symptomatic medication (i.e., not drug-*na*ï*ve*). ^**^Administration of the C-SSRS is recommended if screening question on the PHQ-9 is >0. Core, to be included in all disease-modifying PD trials; Supplemental, recommended but not required for all disease-modification studies in PD depending on the particular trial; Exploratory, may fill current gaps once validation is complete but require further validation; ABC Scale, Activities-Specific Balance Confidence Scale; ACE-III, Addenbrooke’s Cognitive Examination; ADAS-Cog, Alzheimer’s Disease Assessment Scale-Cognitive Subscale; AES, Apathy Evaluation Scale; AS, Apathy Scale; BRAIN, Bradykinesia-Akinesia Incoordination; C-SSRS, Columbia Suicide Severity Rating Scale; CAPSIT-PD, Core assessment program for surgical interventional therapies in Parkinson’s disease; CGI-I, Clinical Global Impression Scale-Improvement; CSF, Cerebrospinal fluid; CSRI, Client Service Receipt Inventory; DRS-2, Mattis Dementia Rating Scale Second Edition; EHR, Electronic health records; EHR, Electronic Health Records; EQ-5D-5L, EuroQoL 5-dimension 5-level questionnaire; eSAPS-PD, Scale for the Assessment of Positive Symptoms in Parkinson’s Disease, enhanced version; ESS, Epworth Sleepiness Scale; FES-I, Falls Efficacy Scale International; FSQ, Functional Status Questionnaire; FSS, Fatigue Severity Scale; GDS-15, 15-item Geriatric Depression Scale; HUI, Health Utility Index; ICECAP, ICEpop CAPability measures; KPPS, King’s Parkinson’s Disease Pain Scale; LARS, Lille Apathy Rating Scale; LEDD, Levodopa-Equivalent Daily Dose; MDRS, Mattis Dementia Rating Scale; MDS-NMS, Movement Disorder Society-sponsored Non-motor Rating Scale; MDS-UPDRS, Movement Disorders Society-sponsored revision of the Unified Parkinson’s Disease Rating Scale; Mini-BESTest, Mini-Balance Evaluation Systems Test; MM4D, Motor fluctuations Monitor for Parkinson’s Disease; MMP, Mini-Mental Parkinson; MMSE, Mini-Mental State Examination; MoCA, Montreal Cognitive Assessment; NfL, neurofilament light chain; NI, not included; NINDS-CDE v2.0, National Institute of Neurological Disorders and Stroke Common Data Elements version 2.0; NMSQ, Non-motor Symptoms Questionnaire; NMSS, Non-motor Symptoms Scale; OPDC, Oxford Parkinson’s Disease Centre; PD-CRS, Parkinson’s Disease-Cognitive Rating Scale; PDQ-8, 8-item version of the Parkinson’s Disease Questionnaire; PDQ-39, 39-tem version of the Parkinson’s Disease Questionnaire; PDQ-Carer, 29-item Parkinson Disease Questionnaire for Carers; PDSS-2, Parkinson’s Disease Sleep Scale-2; PET, positron emission tomography; PHQ-9, 9-item Patient Health Questionnaire; PKG, Parkinson’s Personal KinetiGraph® (formerly Parkinson’s KinetiGraph®); PQoL carers, carers quality-of-life questionnaire for parkinsonism; ProFaNE, Prevention of Falls Network Earth; PROMIS/Neuro-QoL, Patient-Reported Outcomes Measurement Information System/Quality of Life in Neurological Disorders; ROMP, Radboud Oral Motor Inventory for Parkinson’s Disease; S&E ADL SCALE, Schwab and England Activities of Daily Living Scale; SAPS-PD, Scale for the Assessment of Positive Symptoms in Parkinson’s Disease; SCOPA-AUT, Scales for Outcomes in Parkinson’s disease-AUTonomic symptoms; SCOPA-COG, Scales for Outcomes in Parkinson’s disease- COGnitive symptoms; SDQ, Swallowing Disturbance Questionnaire; SF-12, 12-Item Short Form Survey; SF-36, 36-Item Short Form Survey; SPECT, single-photon emission computerized tomography; Supplemental-HR, Supplemental-Highly Recommended; SWAL-QOL, Generic Scale for Dysphagia-Related Outcomes (Quality of Life); TUG, Timed Up and Go; UPDRS, Unified Parkinson’s Disease Rating Scale; UDysRS, Unified Dyskinesia Rating Scale; WOQ-9, 9-item Wearing Off Questionnaire; WOQ-19, 19-item Wearing Off Questionnaire; *α*-syn, alpha-synuclein; A*β*, amyloid beta

This publication also used the National Institute of Neurological Disorders and Stroke Common Data Elements (http://www.commondataelements.ninds.nih.gov/) [3]. More specifically, the NINDS-CDE in PD version 2.0 [4], a NINDS guide to consistently capture and record data across PD studies and to standardize this process to increase comparability of studies, was reviewed. The levels of recommendation for each endpoint (Core, Supplemental – Highly Recommended, Supplemental, Exploratory, Not Recommended), where available, were considered and a modified version was used for classification of outcome measures for disease-modifying trials on the MAMS platform. In short, in the NINDS-CDE classification, “General Core” data elements are required for all NINDS funded studies, “Disease Core” elements collect essential disease-specific (i.e., PD) information and are required for all PD studies, “Disease Supplemental – Highly Recommended” elements have commonly been used and validated in PD and are essential only for some PD studies; “Disease Supplemental” elements are recommended but not required for PD studies and their use varies according to study type, and “Disease Exploratory” elements require further validation but may fill current gaps once validation is complete and can be used as long as their limited or pending validation is acknowledged within the study. We adopted a simplified version of this classification, namely “Core” indicating outcome measure collecting essential PD-specific information to be included in all disease-modifying PD trials (not necessarily as a measure of disease progression); “Supplemental” those that are recommended but not required for all disease-modification studies in PD (i.e., depending on the particular trial); and “Exploratory” those which may fill current gaps once validation is complete but require further validation.

A four-fold strategy was used to include PPIE input. First, data from a recent survey from Parkinson’s UK [5] was reviewed, in which 790 participants (people with PD (PwP) in different stages, partners, carers or family members) reported the symptoms they would most like to see improved. Furthermore, the 2018 European Parkinson’s Disease Association-UCB (EPDA-UCB) survey results were reviewed to extract the most challenging symptoms according to 984 respondents (PwP and families/carers) [6]. In addition to that, a questionnaire was completed by members of the PPIE Working Group of the EJS ACT-PD Consortium about their judgement on the most bothersome symptoms of PD. Measures for the highest-ranking motor and non-motor symptoms in both surveys were included in this list. Whilst this information is primarily relevant to symptomatic treatments, the aim of any disease-modifying treatment would be to reduce functional disability relevant to patients. The results of these surveys on the highest-ranking symptoms for patients inform about the critical aspects of the condition that disease-modifying therapies should aim to delay or ideally avoid. Further input was sought from two PPIE groups (total *n* = 22) about the maximal acceptable duration and frequency of study assessments, either remote or in-person. This approach aimed to guide the maximal number of outcome measures/visit to be included in a disease-modifying PD trial.

All outcome measures identified using the information from the above-mentioned strategies were then evaluated for their potential use in disease-modifying PD trials based on: feasibility, clinical meaningfulness for PwP and clinicians, acceptability to regulators, burden on PwP and clinician, reliability, validity, existence of a suggested clinically meaningful cut-off in PD, sensitivity to change, relevance for specific PD subgroups, interpretability, and current NINDS-CDE classification. When several outcome measures for the same feature arose from the search strategies with comparable properties, the frequency of use of each in clinical trials (obtained via a search in clinicaltrials.gov of the considered outcome measure) and its use in disease-modifying trials (identified in the systematic review (Dr. Marie-Louise Zeissler, unpublished)) together with expert opinion and PPIE input was considered for selection to inclusion.

Compilation of information from all sources was combined in a final report by CGR and AS and reviewed by the expert group. The expert group discussed the information in several meetings, and reviewed several drafts of the list. This methodology is intended to guide future evaluation of evidence on outcome measures for inclusion in future trials in the MAMS platform.

## RESULTS

Triangulation of the above methods resulted in a range of outcome measures on global impression, motor, non-motor features of PD, overall progression, disability, health-related quality of life, resource use, digital and quantitative outcome measures and neuroimaging and wet biomarkers. The full list and classification based on our criteria is shown in Table 1. Further details on the Core outcome measures are included in Table 2, and information on each individual outcome measures included in the above table can be found in the Supplementary Material.

**Table 2:** Brief overview of suggested Core OM in disease-modifying PD trials

Category	Instrument/Test	Brief description	Rater	Delivery	Length (min)	Strengths	Limitations
Global – Generic	CGI-I	7-point categorical scale (level of improvement/worsening) to determine the progress and treatment response of patients	Clinician	In-person or remote	<5	BriefOverall assessmentBroad use in clinical trials	No clinimetric data outside PsychiatrySubjectiveNot PD-specific
Global – PD-related	MDS-UPDRS	PD-specific scale with 4 parts: I: non-motor experiences of daily living (IA and IB) II: motor experiences of daily living III: motor examinationIV: motor complications	IB, II: PatientIA, III, IV: Clinician	In-personDeliverable remotely except for part III (Rigidity and Postural stability items)	30– 40 (whole)	Gold standard OM in most PD trialsComprehensive (motor, non-motor, medication-related complications) Widely used in trialsGood clinimetric propertiesClinically meaningful cut-offs availablePD-specific	LengthyRequires trainingAssociated costsNeeds in-person assessment (part III)Part I: screening of NMSPart III: excessive weight on tremor
	LEDD^*^	Summary of total daily antiparkinsonian medications	Clinician	In-person or remote	<5	PD-specific Widely used in PD (including disease-modifying trials) Potential indirect measure of efficacy	Different methods for calculation, although standard formulae suggested
Motor	Hoehn &Yahr scale	5-stage categorization of PD according to functional disability	Clinician	In-person	<5	PD-specific Brief Excellent clinimetric properties Wide experience in PD clinical trials	Non-granular – less responsive to change than other OMs No minimal clinically important difference
Falls	Question about falls (such as the International ProFaNE falls definition)^*^^*^	One question: In the past *n* months, have you had any fall including a slip or trip in which you lost your balance and landed on the floor or ground or lower level?	Clinician or patient	In-person or remote	<5	Very brief Administrable remotely International definition	Less detailed than other falls scales Not PD-specific
Cognition	MoCA	30-point test assessing different cognitive domains, namely: short-term memory, visuospatial abilities, executive functions, attention, concentration, working memory, language, and orientation to time and place	Clinician	In-person, but deliverable remotely	10 (20 if remote)	Brief Used in PD (including disease-modifying trials) Sensitive to change, less ceiling effect than MMSE Excellent clinimetric properties Clinically meaningful cut-offs defined for PD-MCI and PDD	Requires training Limited sensitivity for specific cognitive domains Low variability of scores (limited sensitivity to change) Not PD-specific
Depression	PHQ-9	Depression module from the PRIME-MD diagnostic instrument for common mental disorders, scores each of the 9 DSM-IV depression criteria from 0 to 3 according to frequency	Patient	In-person or remote	3	BriefUsed in PD (including disease-modifying trials)	Less sensitive to change than others (e.g., GDS-15)Not PD-specific
Disability	S&E ADL	Scale measuring the level of functional independence in 10 levels of ability to perform various chores, distributed in 10% intervals from 0% (“Bedridden”) to 100% (“Completely independent”)	Patient or clinician	In-person or remote	<5	BriefWidely availableUsed in PD (including disease-modifying trials) Good clinimetric propertiesResponsive to change	Not PD-specific
Capability	ICECAP	Scale measuring wellbeing beyond HR-QoL for a more meaningful economic assessment of interventions ICECAP-A (adults) has 5 questions on: stability, attachment, achievement, autonomy, and enjoyment; ICECAP-O (older people) covers: attachment, security, role, enjoyment, and control.	Patient	In-person or remote	<5	BriefEasy to completePreviously used in similar patient populationsFree to useIf collected at repeated timepoints then it allows calculation of CALYs	Not PD-specificCannot be used in standard cost-utility analysis as it does not return QALYs that are required for cost-utility analysis
HR-QoL – Generic	EQ-5D-5L	Measure of perceived health, constituted by 5 items with 5 response options and a VAS on the health status on the day of questionnaire completion, as perceived by the patient, from 0 to 100	Patient or clinician	In-person or remote	<5	BriefWidely used, including in PDGood clinimetric propertiesIf collected at repeated timepoints then it allows calculation of QALYs that can be used in cost-utility analysis, which is commonly used in health technology assessment	Not PD-specificNot as granular as other OMNo clinically meaningful cut-off available
Category	Instrument/Test	Brief description	Rater	Delivery	Length (min)	Strengths	Limitations
HR-QoL – PD-specific	PDQ-8	Short version of the PDQ-39, contains 8 items representing each of the 8 different domains in the PDQ-39, each of them asking about the frequency a PD-related issue on daily life, with 5 possible answers for each of them	Patient	In-person or remote	5	BriefPD-specificGood clinimetric propertiesSensitive to change and responsive to interventionsMinimal important difference availableCan be mapped to utility scores from EQ-5D-3L, so if collected at repeated timepoints then allows approximate calculation of QALYs that can be used in cost-utility analysis, which is commonly used in health technology assessment	Requires a licenseLower reliability and validity than PDQ-39
Resource use	Study-specific combination of CSRI and EHR	Resources used in the treatment and care pathways can be captured from participants/carers using the CSRI questionnaire, and/or from electronic health records, according to the specific study context	Patient, carer, site staff	In-person or remote	5– 20	CSRI can be tailored to meet specific study requirements and capture varied types of relevant resource informationEHR can reduce bias and missing data, and patient burden, and allow data collection outside the trial follow-up periodThe combination of CSRI and EHR to capture resource use allows advantages of each to be maximized and disadvantages minimized	Requires extensive input from trial team and other stakeholders during design of data collection plansCSRI can be burdensome for patients/carers to completeEHR can miss important information as they are not generally designed with research in mindEHR can be expensive to obtain

^*^LEDD is recommended as Core in trials included PD patients taking symptomatic medication (i.e., not drug-naïve). ^**^A question enquiring about falls is recommended as Core, and as an example, the ProFaNE falls definition is described under this section. CALYs, Capability-Adjusted Life-Years; CGI-I, Clinician Global Impression scale – Improvement; CSRI, Client Service Receipt Inventory; DSM-IV, Diagnostic and Statistical Manual of Mental Disorders, Fourth Edition; HER, Electronic Health Records; HR-QoL, Health-Related Quality of Life; ICECAP, ICEpop CAPability measures; LEDD, Levodopa-Equivalent Daily Dose; MDS-UPDRS, Movement Disorders Society-sponsored revision of the Unified Parkinson’s Disease Rating Scale; MMSE, Mini-Mental State Examination; MoCA, Montreal Cognitive Assessment; NMS, non-motor symptoms; OM, outcome measure; PD: Parkinson’s disease; PDD, Parkinson’s disease dementia; PD-MCI, Parkinson’s Disease with mild cognitive impairment; PDQ-39, 39-item Parkinson’s Disease Questionnaire; PDQ-8, 8-item Parkinson’s Disease Questionnaire; PHQ-9, 9-item Patient Health Questionnaire; PPIE, Patient and Public Involvement and Engagement; PRIME-MD, PRIMary care Evaluation of Mental Disorders; QALYs, Quality-Adjusted Life-Years; S&E ADL, Schwab and England Activities of Daily Living Scale; VAS, Visual Analogue Scale.

For *overall assessment* of health, two global status scales were included: the Clinical Global Impression Scale-Improvement (CGI-I) and the Clinical Global Impression Scale-Severity (CGI-S) [7]. We also included the change in Levodopa-Equivalent Daily Dose (LEDD) [8] for trials including PD patients on antiparkinsonian medications.

For assessment of *motor features* of PD, we included: the Hoehn and Yahr scale [9], the Movement Disorders Society-sponsored revision of the Unified Parkinson’s Disease Rating Scale (MDS-UPDRS) [10], the remote versions of both the Unified Parkinson’s Disease Rating Scale (UPDRS) and the MDS-UPDRS [11–13], the UPDRS Gait-axial score [14], and the Unified Dyskinesia Rating Scale (UDysRS) [15]. We also included scales on motor symptoms flagged as most bothersome by PPIE representatives, namely gait and balance problems (question about falls (e.g., Prevention of Falls Network Earth (ProFaNE) definition of a fall [16]), Mini-Balance Evaluation Systems Test (Mini-BESTest) [17], Berg Balance Scale [18], Falls Efficacy Scale International (FES-I) [19], and Activities-Specific Balance Confidence Scale (ABC Scale) [20]), and speech and swallowing issues (Generic Scale for Dysphagia-Related Outcomes (Quality of Life) (SWAL-QOL) [21–24], Swallowing Disturbance Questionnaire (SDQ) [25], and Radboud Oral Motor Inventory for Parkinson’s Disease (ROMP) [26]). Amongst the “Diaries and other fluctuation questionnaires”, the Hauser Diary [27–29], the Core assessment program for surgical interventional therapies in Parkinson’s disease (CAPSIT-PD) On/Off Diary [30], and the 9- and 19-item Wearing Off Questionnaires (WOQ-9 [31] and WOQ-19 [32]) were included.

The following global *non-motor scales and questionnaires* were selected: the Non-Motor Symptoms Questionnaire (NMSQ) [33], the Non-motor Symptoms Scale (NMSS) [34], and the Movement Disorder Society-sponsored Non-motor Rating Scale (MDS-NMS) [35]. Similar to the motor features, specific measures were included for the PPIE-reported most bothersome non-motor symptoms: apathy (Apathy Scale (AS) [36], Apathy Evaluation Scale (AES) [37], and Lille Apathy Rating Scale (LARS) [38]), depression (Geriatric Depression Scale-30 (GDS-30) and GDS-15 [39–41]), 9-item Patient Health Questionnaire (PHQ-9) [42], Columbia Suicide Severity Rating Scale (C-SSRS) [43, 44]), fatigue (Fatigue Severity Scale (FSS) [45]), pain (King’s Parkinson’s Disease Pain Scale (KPPS) [46]), psychosis (Scale for the Assessment of Positive Symptoms in Parkinson’s Disease (SAPS-PD) [47] and its enhanced version (eSAPS-PD) [48]), and sleep (Epworth Sleepiness Scale (ESS) [49], Parkinson’s Disease Sleep Scale-2 (PDSS-2) [50]). Given its relevance and relationship with medication, an autonomic OM was also included (SCales for Outcomes in PArkinson’s disease- AUTonomic symptoms (SCOPA-AUT) [51]).

For *global cognitive measures* we included the Montreal Cognitive Assessment (MoCA) [52–55], the Mattis Dementia Rating Scale Second Edition (DRS-2) [56], the Parkinson’s Disease-Cognitive Rating Scale (PD-CRS) [57–59], the Addenbrooke’s Cognitive Examination (ACE-III) [60–63], the Alzheimer’s Disease Assessment Scale-Cognitive Subscale (ADAS-COG) [64], the Mini-Mental State Examination (MMSE) [65], the Mini-Mental Parkinson (MMP) [66, 67], and the SCales for Outcomes in PArkinson’s disease- COGnitive symptoms (SCOPA-COG) [68].

For *overall progression* of features of PD, Milestone-based outcome measures [69] were included.

*Disability* measures selected for this review were the Schwab and England Activities of Daily Living (S&E ADL) Scale [70, 71], the Functional Status Questionnaire (FSQ) [72, 73], and part II of the MDS-UPDRS [74].

The ICEpop CAPability measures (ICECAP) [75, 76] was included as a *capability* measure. *Carer measures* taken into consideration were the carers quality-of-life questionnaire for parkinsonism (PQoL Carers) [77], the 29-item Parkinson Disease Questionnaire for Carers (PDQ-Carer) [78], and the Zarit Burden Interview (ZBI) [79, 80].

*Health-related quality of life* (HR-QoL) measures were divided into generic (EQ-5D-5L [81, 82], 36-Item Short Form Survey (SF-36) [83, 84], 12-Item Short Form Survey (SF-12) [85, 86], Patient-Reported Outcomes Measurement Information System/Quality of Life in Neurological Disorders (PROMIS/NeuroQoL) [87–90], Health Utility Index (HUI) [91, 92]) and PD-specific (39- and 8- item versions of the Parkinson’s Disease Questionnaire (PDQ-39 [93], PDQ-8 [94])).

*Resource use* data collection methods were also considered, specifically the Client Service Receipt Inventory (CSRI) [95–97] and electronic health records. A brief discussion of requirements for using HR-QoL and resource use information in health economics analysis is also included in the Supplementary Material.

*Digital measures* were divided into active (Oxford Parkinson’s Disease Centre (OPDC) smartphone app [98], CloudUPDRS [99], APDM [100, 101], and mPower smartphone-derived composite score [102]), passive (Parkinson’s Personal KinetiGraph® (formerly Parkinson’s KinetiGraph®) (PKG)-based proxy measures [103], Motor fluctuations Monitor for Parkinson’s Disease (MM4D)-based proxy measures [104], Axivity gait accelerometer [105–107]), and combined active and passive tools (Roche smartphone app [108, 109]).

Furthermore, the following *quantitative motor measures* were considered: Timed-Up and Go (TUG) 3 metre [110], Purdue Pegboard test [111, 112], Alternate tap test [113], BRadykinesia-Akinesia INcoordination (BRAIN) tap test [114, 115], and 9-hole peg test (9hpt) [112, 116–118]. The OPDC composite clinical score [119] was considered under the *composite quantitative motor measures* section.

*Molecular neuroimaging techniques* include dopaminergic single-photon emission computerized tomography (SPECT) [122–129], dopaminergic positron emission tomography (PET) [130–133], non-dopaminergic SPECT [134–140], non-dopaminergic PET [141–155,], and magnetic resonance spectroscopy (MRS) [156, 157].

Considered *structural neuroimaging techniques* [120] were magnetic resonance imaging (MRI) T1 structural sequence [158–162], diffusion imaging [163–166], multiple parametric mapping protocol, neuromelanin [167, 168] and iron-sensitive sequences [169–172].

The following *wet biomarkers* were selected for review [173–178]: plasma/serum neurofilament light chain (NfL) [179–189,], plasma tau [190–195], plasma alpha-synuclein (α-syn) [196–205], cerebrospinal fluid (CSF) neurofilament light chain (NfL) [206–210], CSF tau [211, 212], CSF α-syn [213–215], CSF α-syn aggregation [216–221], CSF beta-amyloid (Aβ) [222–229], and salivary markers, such as salivary α-syn [230, 231].

PPIE input revealed that the mean maximum time per study visit varied depending on the frequency of assessments: for 6-monthly visits, it varied between roughly 2 and 3 hours (longer visits more acceptable when remote), and for yearly visits, between 3 and 3.5 hours. Tables 3 and 4 detail the maximum acceptable length of visits for the PPIE WG and for the PPIE broader engagement group.

**Table 3:** PPIE WG input on maximal acceptable duration of visits (*n* = 10)

	Maximum minutes/visit – Remote assessments		Maximum minutes/visit – Clinic assessments	
	Mode	Mean	Mode	Mean
Monthly	60	72	60	72
Every 3 months	120	102	120	120
Every 6 months	180	138	180	162
Once a year	180	174	>180	198
Less than once a year	180	162	>180	192

**Table 4:** PPIE broader engagement group input on maximal acceptable duration of visits (*n* = 12)

	Maximum minutes/visit – Remote assessments		Maximum minutes/visit – Clinic assessments	
	Mode	Mean	Mode	Mean
Monthly	60	66	60	90
Every 3 months	120	120	180	150
Every 6 months	180	156	180	180
Once a year	180	180	240	210
Less than once a year	240	198	240 / Not acceptable frequency	216

240/Not acceptable frequency: the 2 most common answers were either 240 minutes or “this assessment frequency is not acceptable”.

The set of Core outcome measures proposed in Table 2 would take 70 to 90 minutes (i.e., 1 to 1.5 hours) to complete, making it acceptable to patients according to the above.

## DISCUSSION

We here present an up-to-date inventory of outcome measures for disease-modifying trials in PD based on expert and PPIE consensus. This inventory and framework will be used to guide the decision to select the outcome measures of the EJS ACT-PD MAMS platform trial, based on their fulfilment of desired criteria for an endpoint (e.g., validated, reliable, sensitive to change, acceptable) and their relevance to the intervention based on its mechanism of action and previously known effects (e.g., wet biomarkers as surrogate or direct markers of target engagement). When selecting and classifying the above measures as Core, Supplemental and Exploratory, a compromise had to be made between measures with the best clinimetric properties, acceptability to patients, feasibility, previous experience of use in PD trials, and regulatory considerations, which potentially might have led to prioritizing measures which appear less “promising” from a purely theoretical point of view (i.e., original validation study results) over others, to achieve an adequate balance and provide a realistic and practical tool. This work could also inform other trial initiatives aiming to identify disease-modifying treatments for PD and the framework used will allow updates with new emerging evidence in the future. However, this inventory of outcome measures was created as part of the development of a MAMS trial for progression of PD and as such, presents some particularities which might have influenced the final list of included outcomes. This type of trial requires large participant numbers across a variety of centers, and has a much longer duration than usual randomized controlled trials (RCTs) (i.e., several years) [232, 233]. Therefore, MAMS trials require endpoints which can be measured in different research settings (ideally remotely), and which are sensitive to changes and capture relevant events in disease progression in the longer term. Alternative trial designs, studies focused on particular PD subpopulations, or those looking into changes in a particular aspect of the disease (e.g., cognition, gait) might require an adaptation of this inventory, although it could provide a basis for such adaptations. All of these caveats emphasize the need for a common core set of outcome measures applicable across trial designs and PD populations, to ensure translatability of results regardless of differences between individual trials. Furthermore, it is important to note that this classification does not intend to dictate the choice of primary endpoint, which should be based on the individual trial characteristics (aim, intervention, population, design), and prioritize, among others, sensitivity to change (i.e., detection of disease-modifying effects), relevance, patient acceptability, and feasibility. We refer the readers to regulatory guidance on this subject [234, 235].

Despite being included as exploratory due to the lack of formal validation in this setting, novel outcome measures, and especially digital endpoints, are a promising alternative to complement the currently available instruments. Their potentially increased sensitivity and the possibility of continuous monitoring in real-life conditions (i.e., at home) is likely to be a valuable addition to the administration of scales in the clinical setting. In line with this, a number of initiatives are looking into the clinical validity of these endpoints and their implementation in clinical research [236, 237]. This group selected some of the digital outcomes with more information on PD populations to be included in the inventory. Nevertheless, this is a fast-moving field and recommendations here could require more frequent revision than for other types of endpoints.

The main strength of our approach was strong expert and PPIE consensus, embedding the patient’s voice into the development and recommendation of outcome measures, as well as evidence from literature reviews, information from other initiatives, and input from regulatory bodies.

### Conclusions

With the above methodology, we have identified a broad range of outcome measures which can be potentially included in disease-modifying PD trials, and make recommendations for their inclusion as core, supplementary (for specific arms) and exploratory measures in the EJS ACT-PD MAMS initiative. For other MAMS initiatives, this review aims to serve as a resource from which to select the desired outcome measures according to the requirements of the study (e.g., population, mechanism of action of the intervention, etc.). We also provide a framework for future update of the evidence on outcome measures in disease-modifying PD trials.

## EJS ACT-PD CONSORTIUM MEMBERS

Additional EJS ACT-PD consortium members (further details are provided in the Supplementary material): Roger Barker, James Carpenter, Yoav Ben Shlomo, Mark Edwards, Alan Whone, Carl Counsell, Dorothy Salathiel, Sue Whipps, Anna Jewell, Priti Gros, Tom Barber, Shlomi Haar Millo, K Ray Chaudhuri, Anthony HV Schapira, Oliver Bandmann, Simon Stott, George Tofaris, Esther Sammler, Heather Mortiboys, Li Wei, Alan Wong, Susan Duty, David Dexter, Paula Scurfield, Keith Martin, Edwin Jabbari, Stephen Mullin, Huw Morris, David Breen, Christian Lambert, Prasad Korlipara, Monty Silverdale, Kailash Bhatia, Alison Yarnall, Raj Khengar, Helen Collins, Fleur Hudson, Gareth Baxendale, Rebecca Croucher, Sandra Bartolomeu-Pires, Jennifer Allison, Jodie Forbes, Alex Edwards, Sheila Wonnacott, Dilan Athauda, Joy Duffen, Sonia Gandhi, Emily Henderson, Maryanne Graham, Shona Clegg, Karen Matthews, Vince Greaves, Eric Deeson, Laurel Miller, Joel Handley, David Dexter, Helen Matthews, Kevin McFarthing, Amit Batla, Nikul Bashi, Emma Lane, Miriam Parry, Natasha Ratcliffe, Romy Ellis-Doyle, Sally L Collins, Rebecca Chapman, Jesse Cedarbaum, Anthony Lang, Brian Fiske, Richard Wyse, Mahesh Parmar, Adam Boxer, Denise Wilson, Jean Christophe Corvol, Jennifer Harris.

## Supplementary Material

Supplementary Material 1

Supplementary Material 2

## Data Availability

Data sharing is not applicable to this article as no datasets were generated or analyzed during this study.

## ADDITIONAL STATEMENT

For the purpose of open access, the author has applied a Creative Commons Attribution (CC BY) license to any Author Accepted Manuscript version arising from this submission.
