# Supplementary material for: Outcome Measures for Disease-Modifying Trials in Parkinson’s Disease: Consensus Paper by the EJS ACT-PD Multi-Arm Multi-Stage Trial Initiative
Source: J Parkinsons Dis. 2023 Sep 8;13(6):1011–33. doi: 10.3233/JPD-230051 (PMC10578294; doi:10.3233/JPD-230051)
Supplement: Supplementary Material 1 [file jpd-13-jpd230051-s001.pdf]

Supplementary Material 1. Classification of considered outcome measures.

Global outcome measures (OM)

A 2016 publication analysed four measures of global severity in PD: Hoehn and Yahr (HY), Clinical Global Impression of Severity (CGI-S), Clinical Impression of Severity Index (CISI-PD), and Patient Global Impression of Severity (PGI-S). The authors concluded that all four scales were moderately to strongly related between them, that the clinician-based assessments correlated higher than patient-reported ones, and that the CISI-PD showed the greatest association with all PD measures (motor, non-motor, disability, and patient-reported HR-QoL) [1].

However, the CISI-PD has been less frequently used in PD studies (7 trials in clinicaltrials.gov as of October 11th, 2022), when compared with the CGI-I (77 studies) and the CGI-S (40 studies), so the last two are more comparable to data from previous studies.

We have also included the change in Levodopa-Equivalent Daily Dose (LEDD) (8) for trials including PD patients on antiparkinsonian medications.

Clinical Global Impression Scale (CGI) – Improvement (CGI-I) and Severity (CGI-S)

The CGI is a clinician-rated instrument to determine the progress and treatment response of patients, originally developed in Psychiatry, but currently used in other disciplines. Two of its components are the CGI severity (CGI-S) and improvement (CGI-I) scales [2].

The CGI-I is classified as Supplemental – Highly Recommended by the NINDS-CDE version 2.0, and the CGI-S is classified as Supplemental by that initiative.

Strengths

Both the CGI-I and the CGI-S are very brief and thus, extremely easy to complete. They consist of just one question each, to be answered in a 7-point categorical scale (level of improvement/worsening and severity of illness, respectively). They provide a global vision on the participant's situation, and there are participant-reported versions of both (PGI-I and PGI-S). Furthermore, they can be administered remotely. The CGI-S correlates well with other global assessment tools, such as the Clinical Impression of Severity Index scale [1].

Limitations

Despite its relatively wide use in PD clinical studies, there are no comprehensive clinimetric assessments of the CGI-I in non-psychiatric conditions. Furthermore, in both the CGI-I and the CGI-S, there is no clear guidance on how to apply this tool, and the language utilised is subjective and open to interpretation.

Levodopa-Equivalent Daily Dose (LEDD)

The LEDD consists of a calculation of the total daily dose of antiparkinsonian medications in a given PD patient [3].

The LEDD is not included in the NINDS-CDE version 2.0.

Strengths

The LEDD is brief, easy to calculate, and gives an overall indirect estimation of a patient's severity through their medication requirements. The change in LEDD has been widely used as an endpoint in PD studies, including disease-modifying clinical trials [4].

Limitations

Different methods have been described in the literature to calculate the LEDD, although standardised formulae have been suggested [3].

Motor scales

The NINDS-CDE version 2.0 include the Hoehn and Yahr (H&Y) scale, the UPDRS and the MDS-UPDRS as Core elements. The authors advise to “select either MDS-UPDRS (for studies focused on all severities and especially on mild/moderate participants) or UPDRS (for studies focused preferentially on advanced PD participants)”. We also included the H&Y and the MDS-UPDRS in this list.

Other scales included in this section are also considered within the NINDS-CDE version 2.0, and their degree of recommendation of each instrument is listed in its corresponding subsection.

MDS-UPDRS (motor and non-motor components) and UPDRS

The Movement Disorders Society-sponsored revision of the Unified Parkinson’s Disease Rating Scale (MDS-UPDRS) [5], a revision of the Unified Parkinson’s Disease Rating Scale (UPDRS), is currently the gold standard outcome measure in most PD trials [5]. It is divided into four sections: non-motor experiences of daily living (parts IA and IB), motor experiences of daily living (part II), motor examination (part III), and motor complications (part IV). Of those, parts IB and II are patient-completed, whereas

parts III and IV are clinician-administered. The NINDS-CDE classifies the MDS-UPDRS and the UPDRS as Core outcome measures in PD.

Strengths

This scale is comprehensive and covers a wide range of motor and non-motor features and medication-related complications of PD. It is partly patient reported, has good clinimetric properties and is widely available and used. It has already been used in disease-modifying trials in PD (part III more frequently in "off" than in "on" status). Since it assesses various aspects of PD, including motor and non-motor, it is clinically meaningful for both patients and clinicians, and the fact that it is widely used, includes patient-reported subscales and has well establish clinimetric properties makes it likely to be acceptable to regulatory bodies.

The clinimetric properties of the MDS-UPDRS are strong. In the original paper [5] it showed excellent internal consistency (Cronbach's alpha – 0.79-0.93), is strongly correlated with the UPDRS, and adequate test-retest reliability has been reported in the Spanish version of the scale [6]. The MDS-UPDRS has adequate test–retest reliability (all but two subscores showed $k > 0.60$) [6]. To the best of our knowledge, there is no published data on the MDS-UPDRS inter-rater reliability, but there is information on the within-subject reliability of this scale over one year [7]. Furthermore, there is extensive data on the UPDRS and the MDS-UPDRS development resolved ambiguities and corrected inconsistencies in the UPDRS, inferring greater inter-rater reliability. For the motor part of the UPDRS, a study with three experienced neurologists indicated good-to-excellent agreement for speeded repeated movements, resting tremor, arising from a chair, and gait; moderate agreement for action tremor, rigidity, posture, postural stability, and bradykinesia; and poor agreement for speech disorder and facial immobility [8]. Another group reported substantial agreement between neurologists and

advanced practice nurses on the mean ratings of the motor section of the UPDRS (ICC = 0.65), and moderate agreement for ascertaining whether all items of that UPDRS part were normal (kappa = 0.53) [9]. A further study on the agreement between nurse practitioners, residents in neurology, junior and senior movement disorders specialists on the motor UPDRS scores reported good inter-rater and intra-rater reliability for the majority of individual UPDRS motor items and for the sum score of the motor section [10]. Nevertheless, there were considerable differences between the senior specialist and the other raters, with the latter assigning higher scores, with mean between 1.7 and 5.4 and broad limits of agreement. The authors conclude that disagreement between raters, including the extent of their biases, should be quantified prior to starting PD clinical trials. In another small study, inter-rater reliability between movement disorder neurologists and PD specialist nurses measured using the intra-class correlation coefficient revealed ICC 0.95 for part III and 0.96 for part IV. The greatest was found for gait (ICC = 0.746; P<.0001) and the lowest, for postural stability (ICC = 0.918; P<.0001) [11]. It has been suggested that some items pose a greater challenge than others in regards to inter-rater reliability [8], that mild PD is more likely to receive heterogeneous scores across raters, and that appropriate teaching is key to minimising inter-rater variability [12].

Clinically meaningful cut-offs have been established for each of the scale's parts, and they differentiate between mild/moderate PD and moderate/severe PD [13].

It provides information on presence (all parts), impact (I, II, IV) and severity (all parts) of symptoms.

Limitations

Permission for use from the MDS is required and there are costs associated with commercial studies. Raters need to obtain an MDS-UPDRS Training Certificate via the

MDS website. It is relatively long to complete in its entirety (around 30-40 minutes). There is no statistical support for the combination of part III with other parts of the MDS-UPDRS [14]. However, the combination of parts I and II has already been studied as an outcome measure, and clinimetric properties for this combination are relatively favourable [15].

Remote UPDRS

A modified version of the motor section of the UPDRS (mUPDRS) without rigidity and retropulsion pull testing that could be administered remotely was presented some years ago, along with data on its reliability and validity [16]. The NINDS-CDE has not yet classified the Remote UPDRS in its system, but all digital health outcome measures are still considered Exploratory.

Strengths

The mUPDRS showed to be cross-sectionally (ICC = 0.92) and longitudinally (ICC = 0.92) reliable when compared with the motor part of the UPDRS. Furthermore, it demonstrated high internal consistency (Cronbach's alpha = 0.96) and high concurrent validity with the standard UPDRS ($r = 0.93$, $p < 0.0001$).

Limitations

This scale could replace the motor part of the UPDRS in those instances when face-to-face assessments are impossible. However, apart from those scenarios, this measure is exploratory for the moment and priority should be given to face-to-face assessments with the standard MDS-UPDRS. It is particularly unsuitable for PD patients with postural instability and gait disturbance (PIGD), as well as patients with a rigidity-predominant phenotype, since these features cannot be assessed through this scale and

hence progression or improvement are not detected. Furthermore, with exclusion of bradykinesia the scale becomes more heavily weighted towards the items on tremor. The mUPDRS has not been used in disease-modifying trials in PD. Apart from patient and clinician's burden (necessary video access, etc.) and the absence of data on sensitivity to change, this scale is not as meaningful as the full motor part of the UPDRS or MDS-UPDRS, and therefore is less likely to be accepted by regulators.

Remote MDS-UPDRS

A feasibility study on administration of the whole MDS-UPDRS via video conference has also been published [17], and it seems to be a useful measure of overall symptom severity over time, but technical constraints and the burden on patients (video conference, etc.) supports the utilisation of the classical MDS-UPDRS instead.

As with the remote UPDRS, the NINDS-CDE has not yet classified the Remote MDS-UPDRS in its system, but all digital health outcome measures are still considered Exploratory, and similar limitations exist as for the remote UPDRS.

8-item Remote MDS-UPDRS

Recently, an 8-item version of the MDS-UPDRS has been developed, which can be administered remotely [18]. This version was selected through an exhaustive search and analysis of all possible subsets of the 50 MDS-UPDRS items. The scale comprises the following items: (1.13) fatigue, (2.5) dressing, (2.10) tremor, (2.12) walking and balance, (3.2) facial movement, (3.4) finger tapping, (3.9) arising from chair, and (4.3) time spent in the off state.

Strengths

This subset was the most highly correlated to the MDS-UPDRS full score (Pearson's r 0.919, p-value <0.0001), and had an explained variance score of 0.844, while still being administrable remotely. The high agreement of this subset with the total MDS-UPDRS supports its use in situations where practicalities limit the application of the full MDS-UPDRS.

It is brief, reducing the burden for patients and clinicians, and as a remote measure, it is more feasible than other instruments. Since it includes items from all 4 parts of the MDS-UPDRS, it gives a global estimation of the patient's status.

Limitations

Since it has been very recently published, this scale has not yet been used in disease-modifying trials in PD, which limits its acceptability to regulators.

Consequently, this subscore now will need validation in other PD cohorts and a full clinimetric analysis, but the results from the original developers are promising.

MDS-UPDRS Gait-axial score

This outcome measure comprises the sum of the gait and axial components of the MDS-UPDRS motor examination. It was identified through factor analysis of the UPDRS part III, which revealed that a combination of 7 items relating to gait and axial measures accounted for the greatest variance both cross-sectionally (in the 'on' and 'off' state) [19,20] and longitudinally (over a mean (SD) follow-up of 5.5 (1.4) years) [21]. Although originally defined based on the original UPDRS, the 7 items are retained within the MDS-UPDRS and the score can be similarly derived from this scale. The component items include speech (item 3.1), facial expression (3.2), rising from chair (3.9), gait (3.10), postural stability (3.12), posture (3.13), and body bradykinesia (3.14).

The NINDS-CDE has not yet classified the MDS-UPDRS Gait-Axial score in its system.

Strengths

Although this score was first defined over 20 years ago, it has been slow to be adopted into clinical trials, but it is now being used in a disease-modifying trial of azathioprine for PD (AZA-PD, ISRCTN14616801). It is as feasible as the MDS-UPDRS, but of considerably shorter duration, hence the burden for both patients and clinicians is low. It does however require MDS-UPDRS rater training. Its reliability can be inferred from that of the MDS-UPDRS part III and it has shown a good relative validity when compared with a self-rated assessment of day to day symptoms and symptom severity [22], to Hoehn and Yahr stage, and to the Schwab and England independence scale [19]. Akin to the MDS-UPDRS, this scale is interpretable at the level of presence and severity of motor signs. A key strength of this score is its sensitivity to change: a factor analysis indicated that this gait-axial component of the UPDRS accounts for the greatest proportion of variance in longitudinal change in UPDRS part III [21]. Another advantage is that speech, posture, gait, postural stability and rising from sitting, which together account for a large proportion of this score, are considered ‘dopa-resistant’ [23]. As such, this subscore may be less confounded by increases in dopaminergic medication than is the case for the total MDS-UPDRS III, thus rendering it particularly suitable for employment in trials of putative disease-modifying agents. Indeed, the ‘dopa resistant’ axial score showed high sensitivity to change over time in an incident longitudinal cohort followed for a mean of 5 years [24]. A similar UPDRS axial score (comprising rising from chair, gait, postural stability, and posture), was also shown to be predictive of a poor 5-year outcome in 2 incident cohorts [25].

Limitations

There is currently little experience of using this measure in a clinical trial setting, and formal clinimetric testing of this subscore has not been carried out, although both the UPDRS and the MDS-UPDRS have extensive data on clinimetric properties, and this measure is embedded within them. Its acceptability to regulators may be lower than other scales as it is dependent solely on motor examination and does not include patient-reported components. Nevertheless, given its solid rationale, wider use in different settings and a formal clinimetric validation will likely lead to the MDS-UPDRS gait-axial score being adopted as an outcome measure in trials other than AZA-PD in the near future.

Hoehn and Yahr (H&Y) scale

The H&Y scale categorises PD patients according to their functional disability in 5 stages (1-5): 1 = unilateral involvement, with minimal or no functional disability; 2 = bilateral or midline involvement without impairment of balance; 3 = bilateral involvement with impaired postural reflexes, physically independent; 4 = severely disabling disease, still able to walk or stand unaided; 5 = confined to bed or wheelchair unless aided [26]. A modified version of the H&Y was created but it showed weaker clinimetric properties so the MDS encourages the use of the original H&Y [27].

Strengths

Having been developed in 1967, there is abundant experience with this scale, which is also PD-specific, brief to administer, and has shown excellent clinimetric properties, especially reliability and convergent validity with other OM in PD [27]. Axial symptoms (e.g., balance, gait), which are known to be less dopa-responsive, are a component of this scale, which could make it attractive for disease-modifying trials,

helping reduce the confounding effect of dopaminergic therapy. The progression to stage 3 has been defined as an important event in the course of the condition [27], and recording the H&Y stage can be potentially useful in the development of milestone-based OM.

Limitations

Its lack of granularity and brevity make the H&Y a less responsive scale to change than other instruments, and on its own it would not suffice as a motor OM in a disease-modifying trial. Being a categorical rather than a continuous scale, a minimal clinically important difference (MCID) cannot be established for the H&Y [27].

Unified Dyskinesia Rating Scale (UDysRS)

The Unified Dyskinesia Rating Scale (UdysRS) was developed by the MDS and presented in 2008 [28], including its clinimetric properties. It has four parts: I: Historical Disability (patient perceptions) of On-Dyskinesia impact (maximum 44 points); II: Historical Disability (patient perceptions) of Off-Dystonia impact (maximum 16 points); III: Objective Impairment (dyskinesia se- verity, anatomical distribution over seven body regions, and type (choreic or dystonic) based on four activities observed or video-recorded (28 points); IV: Objective Disability based on Part III activities (maximum 16 points). At the time of the latest MDS critique paper on dyskinesia scales, there was only evidence to classify the UDysRS as “Suggested” [29], but its use has increased since then and with it, the experience of its performance in clinical trials in PD.

The NINDS-CDE version 2.0 has classified the UdysRS as Supplemental – Highly Recommended.

Strengths

The UdysRS has been used in both symptomatic and disease-modifying trials in PD. It is brief (15 minutes to complete), widely available, and has excellent reliability: in its original paper, both subjective (I and II) and objective (III and IV) sections showed high internal consistency (Cronbach's alpha: 0.915, 0.971) acceptable interrater and intra-rater reliability, and reliable factor structures were found [28].

The minimally clinical important difference (MCID) for the historical parts of the UdysRS have already been calculated [30], which further aids interpretation of results along a clinical trial. It is useful in treated PD patients, who might experience dyskinesias, and it is interpretable at the level of presence and severity of dyskinesias. Regarding responsiveness of the UdysRS, in a randomised clinical trial of amantadine versus placebo using four different dyskinesia rating scales, the UDySRS Total Score showed the greatest effect size ($\eta 2 = 0.138$) for detecting treatment-related change [31].

Limitations

The UdysRS requires a physical assessment and clinician training, which implies a moderate burden for both patient and clinician. As mentioned above, the UdysRS was classified as "Suggested" by the MDS [29] because sensitivity to change had not yet been tested, and it had not yet been studied by other groups. Both of those issues have now been overcome, which would make the UdysRS qualify as Recommended. As opposed to the UdysRS, the other promising dyskinesia rating scale on the MDS critique paper [29], the Parkinson Disease Dyskinesia Scale (PDYS-26), has not had, to our knowledge, its sensitivity to change tested, and has been scarcely used.

Although the Rush Dyskinesia Scale and the Abnormal Involuntary Movements (AIMS) Scale were initially "Recommended" by the MDS [29], caveats on both of

them, especially on the AIMS Scale, were already mentioned in that paper. Furthermore, they are less commonly used in PD trials and in the case of the AIMS, to our knowledge, it has not been used in disease-modifying trials.

Specific scales on especially bothersome motor symptoms

As described above, we extracted information from the results of a Parkinson's UK survey on people with PD and carers [32], and the responses from an internal questionnaire in our PPIE Working Group on the most impactful symptoms of the condition. A combination of the results indicated that the most relevant symptoms for people with PD are the following: fatigue, pain, sleep problems, psychological problems (depression, apathy, hallucinations/psychosis), gait/balance/falls and speech/swallowing. Of those, the latter two are motor symptoms and thus specific rating tools for them are reviewed below.

Gait, balance and falls

In the latest MDS review of instruments to assess balance, gait and posture, the following were classified as "Recommended": 1) Rating scales: UPDRS-derived Postural Instability and Gait Difficulty score; 2) Scales requiring equipment: Berg Balance Scale, Mini-BESTest, and Dynamic Gait Index; 3) Questionnaires: Freezing of Gait Questionnaire, Activities-Specific Balance Confidence Scale, Falls Efficacy Scale, Survey of Activities, and Fear of Falling in the Elderly–Modified; and 4) Tests: 6-minute and 10-m walk tests, Timed Up and-Go, and Functional Reach.

Some of these tools are reviewed below. Nevertheless, the authors concluded that there is no single instrument that reviews all PD-specific gait features with a satisfactory clinimetric profile, and no scales with separate scores for balance and gait that shows good content validity in PD [33]. Furthermore, none of the above-mentioned instruments has been formally classified by the NINDS-CDE initiative. In a more recent systematic review of functional mobility measures in PD, the authors concluded that only the Timed Up and Go test met criteria to be recommended [34].

A systematic review of tools to measure balance and predict risk of falls in PD concluded that, out of 68 outcome measures, only the following 6 have acceptable psychometric properties: Mini-Balance Evaluation Systems Test (Mini-BESTest), Berg Balance Scale, Timed Up and Go test, Falls Efficacy Scale International, Activities-Specific Balance Confidence scale, and the Motor Examination of the UPDRS [35]. Of those, the MDS-UPDRS is reviewed above, and the Timed Up and Go has been described under “Quantitative motor measures”.

The NINDS-CDE v2.0 includes no specific gait or balance measurement tools.

Apart from the quantitative motor measures described below in the document (e.g., Timed Up and Go) and the digital measures which assess gait, in this section we have included a brief definition of falls, 2 measurement tools which require additional materials, and 2 scales which can potentially be administered remotely.

Prevention of Falls Network Earth (ProFaNE) Definition of a Fall

We recommend including a question about falls since last visit as a Core measure for disease-modifying PD trials, and present the ProFaNE definition of a fall [36] as an example of this mainly due to its conciseness. It is simply one question for patients: “In

the past *n* months, have you had any fall including a slip or trip in which you lost your balance and landed on the floor or ground or lower level?"

Strengths

It is very brief, administrable remotely and used widely, and the timeframe (i.e., number of months) can be adapted depending on the study's scope.

Limitations

It is less detailed than some of the below instruments (e.g. does not include frequency or cause of falls), and it is also not PD-specific. Furthermore, it enquires about loss of balance, which might lead to under-reporting of falls due to other PD-related causes (e.g. freezing of gait).

Mini-Balance Evaluation Systems Test (Mini-BESTest)

Strengths

The Mini-BESTest is a shorter version of the BESTest [37]. It includes 14 items to assess gait, stability, and posture, and takes about 10-15 minutes to complete. It is overall a good measure.

Limitations

This test requires many additional accessories (stopwatch, measuring tape, chair, item to pick from floor, stepping stool), which makes it less feasible depending on the setting [33].

Berg Balance Scale

Strengths

The Berg Balance Scale assesses functional balance initially validated in an elderly population. It comprises 14 tasks which evaluate both dynamic and static balance. It is brief and easy to administer in different settings [38]. It has been shown to have good internal consistency, test-retest reliability, validity and responsiveness.

Limitations

Similar to the Mini-BESTest, it requires several accessories (foam block, chair, ramp, obstacle, stopwatch, and a 6-m walkway), which can potentially reduce its applicability in some settings [33].

Falls Efficacy Scale International (FES-I)

Strengths

This is a 10-item questionnaire assessing the fear of falling and ability of the respondent to avoid falls in daily life activities [39]. It only takes 5 minutes to complete.

Limitations

Some activities are not included in the questionnaire, and it must be considered that fluctuations due to medication status can impact the score [33].

Activities-Specific Balance Confidence (ABC) Scale

Strengths

The ABC scale, as mentioned in its name, evaluates the confidence of the respondent in their balance [40]. It has 16 items, takes 5 to 20 minutes to complete, and has good clinimetric properties.

Limitations

As with the Falls Efficacy Scale, the results may vary depending on medication status, and some items are culture-specific [33].

Speech and swallowing

Speech impairments are covered in several outcome measures outlined below. Regarding swallowing, the most recent MDS review and critique paper on dysautonomia rating scales [41] includes dysphagia assessment tools. The authors concluded that the videofluoroscopic swallow study (VFSS) is the ‘‘gold standard’’ for the diagnosis of dysphagia in PD, and that no scales met criteria to be “Recommended” (namely, to be considered valid, reliable, and sensitive, to be reported in clinical studies beyond the group that developed it, and to be applied to PD populations). However, two dysphagia scales met criteria to be “Suggested”: The Swallowing Disturbance Questionnaire (SDQ) and the Generic Scale for Dysphagia-Related Outcomes (Quality of Life) (SWAL-QOL). Of those, the latter showed greatest promise given its good results in clinimetric testing in varied dysphagia populations. The SWAL-QOL has been used in 7 PD clinical trials registered in clinicaltrials.gov, and the SDQ, in 8 studies, none of which are disease-modifying.

A 2017 systematic review of dysphagia measures [42] identified another speech and swallowing instrument apart from the SDQ: the Radboud Oral Motor Inventory for Parkinson’s Disease [43].

The NINDS-CDE version 2.0 has classified all 3 measures as Supplemental in PD.

Generic Scale for Dysphagia-Related Outcomes (Quality of Life) (SWAL-QOL)

Strengths

The SWAL-QOL is a dysphagia-specific quality of life questionnaire, completed by patients, which aims to detect the usefulness of therapeutic interventions. It was developed for patients with mechanical or oropharyngeal dysphagia of different aetiologies [44–47].

It comprises 10 domains with 44 items in total, to be answered in a 5-point scale (1-5).

It has robust clinimetric properties and correlates well with generic measures. It is acceptable, patient-reported, and has a low burden despite taking longer than 10 minutes to complete [41].

Limitations

The SWAL-QOL was validated in a mainly English-speaking population, and in static dysphagia, which compromises its interpretability in a progressive and fluctuating condition such as PD [41].

To the best of our knowledge, the SWAL-QOL has not been formally validated in PD [48], but it is widely used in this condition [49,50].

Swallowing Disturbance Questionnaire (SDQ)

The SDQ is a PD-specific 15-question screening tool for dysphagia [48]. At the time of the MDS review and critique paper, it was "Suggested" because it had only been tested in a single PD population, and only some clinimetric data had been reported. However, since then, the SDQ has been validated in various languages and used by different groups [51–54], which would make it "Recommended".

Strengths

Due to its brevity, it has a low burden on patients [41]. It combines 4-point Likert scale and dichotomous (yes/no) items [42].

Limitations

Although validated in PD, internally consistent, several clinimetric aspects were not reported, and the validation sample was small.

Radboud Oral Motor Inventory for Parkinson's Disease (ROMP)

Since that review was published, another interesting measure for speech, swallowing and drooling problems in PD has been developed: the Radboud Oral Motor Inventory for Parkinson's Disease (ROMP) [43].

Strengths

It is a patient-reported measure of speech, swallowing and saliva control, with 23 items (7 of them in the swallowing domain) that must be answered on a 5-point Likert scale [42,43], and its swallowing domain has been rated as of high quality in a recent systematic review of dysphagia patient-reported outcome measures [42]. Its clinimetric testing was satisfactory, with high internal consistency (0.95 for the total ROMP and 0.87 to 0.94 for the 3 subscales), high reproducibility (ICC 0.94 for the total scale, 0.83 to 0.92 for the subscales), and substantial-to-good construct validity (correlations: 0.36 to 0.82). It also showed good sensitivity to change (differentiating significantly between patients in need of speech therapy and those who were not) and to PD stages (mild, moderate and severe based on Hoehn and Yahr (HY)). Interestingly, this scale exists in Dutch as well [42], and has already been translated to Brazilian Portuguese, showing excellent reliability and validity [55]. Furthermore, a recent review on drooling rating scales in PD showed that the only tool with solid evidence of its clinimetric profile and

data in PD was the ROMP-saliva [56], further strengthening the arguments to select this self-reported scale as a screening tool of speech, swallowing and drooling problems in PD.

According to the 2009 MDS review of dysphagia scales [41], the ROMP meets criteria to be “Recommended”: it has been validated showing good clinimetric properties [43], used beyond the original investigators (3 studies in clinicaltrials.gov are using the ROMP), and used in different PD populations [55].

Limitations

Being a more recent scale, its use in PD clinical trials is more limited.

Diaries and other fluctuation questionnaires

The MDS review and critique paper on wearing-off scales in PD [57] classified them as “Recommended” if they met the following three conditions: to have been applied in PD, to have been used beyond the original authors, and to have had their screening properties tested. According to those criteria, the following tools met the conditions to be “Recommended”: among screening questionnaires, the 19-item and the 9-item Wearing-OFF Questionnaires (WOQ-19 and WOQ-9) and among scales, Motor Fluctuation Diaries (of which the most common is the Parkinson’s Disease Diary, developed by Hauser and colleagues), and the Core Assessment Program for Surgical Interventional Therapies in Parkinson’s Disease (CAPSIT-PD) Diaries. All four of them are covered in more detail below.

Diaries can be used for health economic analysis, either as diaries, or as information collected at time intervals (e.g., 3 months), or from records, or a combination of all

three. They allow for the collection of more detailed data, with less risk of recall bias if they are collected very frequently. However, as with HR-QoL measures, the burden of diaries in the case of patients and carers is medium to high due to their potential length and the required frequency of completion, and minimisation of the gathered data is warranted to reduce the burden.

PD Home Diary (Hauser Diary)

The Parkinson's Disease Home Diary [58,59] is a self-reported measure of motor fluctuations, i.e., time in "on", "on with non-troublesome dyskinesia", "on with troublesome dyskinesia", "off", and "asleep". The patient chooses one of those five options every 30 minutes during the day.

A Hauser diary with pictograms and a visual analogue scale to rate severity of dyskinesia in each diary entry has also been developed, with a training video for patients previous to completion of the diary [60].

Given the greater background evidence, this section focuses on the traditional PD Home Diary [58].

The NINDS-CDE version 2.0 has classified the Hauser Motor Fluctuation Diary as Supplemental – Highly Recommended in PD (indicated for studies assessing motor fluctuations).

Strengths

The Hauser diary has been frequently used in clinical trials in PD, including trials of disease-modifying agents, and is widely available. As a patient-reported outcome which details motor fluctuations, it is clinically meaningful to both patients and clinicians, and

is likely to be accepted by regulatory bodies. Since it is a self-reported measure, the burden on the clinician is low.

Regarding its clinimetric properties, the mean percentage of the awake day with “good on” is a very stable parameter over time. It is highly reliable (Cronbach’s alpha in consecutive completion days: 2 days, r = 0.806; 3 days, r =0.868; 4 days, r =0.918; 5 days, r =0.934; 6 days, r =0.946) has good test–retest reliability, which increases as does the number of diary days. Its intraclass correlation coefficient (ICC) is greater than 0.70 (0.715, 95% one-sided lower limit: 0.710) and the results are not influenced by age, sex, or country [59]. The Hauser diary was reported to have good predictive validity by its original authors [59], but this was only moderate when estimating the strength of association with 5 visual analogue scale items (R = 0.36–0.57) [57]. It is interpretable at the level of presence and severity of symptoms.

Limitations

As a self-reported patient measure which requires completion every 30 minutes, it imposes a considerable burden on patients, and consequently it has been described that compliance diminishes beyond 3 days [59]. Fluctuation diaries like the Hauser diary require and assume an understanding of the different motor states of PD by the patient, to ensure accurate reporting. Since the diary’s sensitivity to change depends on the number of days that it is filled by the patient [59], compliance problems are a relevant caveat for this measure.

CAPSIT-PD On/Off Diary

Both the Hauser and the CAPSIT-PD diaries were “Recommended” in the last MDS critique paper on wearing-off scales in PD, although the latter was reported to have caveats [57].

Akin to the Hauser diary, the CAPSIT-PD diary measures motor fluctuations, i.e., time in “on”, “on with dyskinesias”, “partial off” and “off”, daytime every 30 minutes.

The NINDS-CDE version 2.0 has classified the CAPSIT-PD as Supplemental – Highly Recommended in PD (indicated for studies assessing motor fluctuations).

Strengths

Similar to the Hauser diary, the CAPSIT-PD diary is a clinically meaningful measure and since it is patient-reported, it is also likely acceptable by regulators. As a self-reported measure, clinician burden is low, implying at most training of patients to ensure full understanding of the different motor states before diary completion.

In terms of clinimetric properties, the overall patient–clinician agreement of the CAPSIT-PD diary during a 4-hour period was good (k = 0.62; weighted k = 0.84). Agreement for individual diary categories was good for “off” and “on with dyskinesias” (k ≥ 0.72) [61].

The CAPSIT-PD diary showed good predictive properties in data extracted from a 1-week period for all items except for “partial off” [57,61].

Limitations

Unlike the Hauser diary, to the best of our knowledge, the CAPSIT-PD diary has not been used in disease-modifying trials in PD, and it is used less frequently in PD trials in general than the Hauser diary.

The patient burden is again relevant, as it requires filling out every 30 minutes to fully understand the various response options.

Patient-clinician agreement of the diary was only moderate for “partial off” and “on” states (k = 0.49), and “partial off” could not be properly predicted even with two weeks’ data [61].

WOQ-9 and WOQ-19

The 9- and 19-item Wearing-Off Questionnaires (WOQ-9 [62], WOQ-19 [63]) are shorter versions stemming from the original 32-item Wearing-Off Questionnaire (WOQ-32) [64]. Both of them were “Recommended” as wearing-off scales by the MDS [57]. Interestingly, there is also the QUICK questionnaire (QQ), a Spanish validated version of the WOQ-19 [65].

The NINDS-CDE version 2.0 has classified the WOQ as Supplemental – Highly Recommended in PD.

Strengths

Both WOQ-19 and WOQ-9 have good sensitivity [57] which was also confirmed in a recent systematic review of both questionnaires (sensitivity = 0.81-1) [66].

For the WOQ-19, both a 1 positive item and a 2 positive items cut-off have been described [66], the latter displaying a sensitivity of 0.881 and a specificity of 0.674. Regarding test-retest reliability, the ICC of number of positive items of the WOQ-19 has been reported to be 0.858 for wearing-off-related symptoms as a whole [67].

The WOQ-9 has been used in several studies in PD, including disease-modifying trials.

Both scales are clinically meaningful, constitute patient-reported outcomes, and the burden on both clinicians and patients is low, although it requires patient understanding of the different PD motor states and in the case of the WOQ-19, it is a longer questionnaire.

There is a significant correlation between the Italian version of the WOQ-19 and the UPDRS motor section and Hoehn and Yahr (HY) scores [68].

In terms of responsiveness, there is one clinical trial with cathecol-O-methyltransferase (COMT) inhibitors, in which Cohen's effect size for the QQ was 0.5 [69].

Limitations

The WOQ-9 lacks specificity (0.1-0.69) [57,66], and in the case of the WOQ-19, it is variable (0.39-0.8), probably due to combination of studies with a 1-item and a 2-item cut-off [66].

On the other hand, the WOQ-19 has been only rarely used in PD trials, and so the experience with it in this setting is scarcer than that of the WOQ-9.

Non-motor scales and questionnaires

Non-Motor Symptoms Questionnaire (NMSQ)

The Non-Motor Symptoms Questionnaire (NMSQ) is a 30-item self-completed questionnaire on non-motor symptoms of PD, which can be grouped into relevant domains [70]. Both the NINDS-CDE and NINDS-CDE version 2.0 have classified the NMSQ as Supplemental for use as an outcome in PD patients.

Strengths

The NMSQ is available through the MDS, although costs incur if it is used in clinical studies and trials. As a patient-reported measure, it is clinically meaningful for both patients and clinicians, and the burden for both parties is low as the questionnaire is short and easy to complete (yes/no questions).

Overall, it provides an overview of the presence of specific non-motor symptoms in PD patients, and additionally clinically meaningful grading scores for the NMSQ total scores have been suggested for the NMSQ (0 = No NMS; 1–5 = Mild; 6–9 = Moderate; 10–13 = Severe; and >13 = Very severe) [71].

There is good reliability data on the original English version, as well as the Italian and Chinese versions of the questionnaire [71–73].

The questionnaire is relevant for all PD patients and can be useful to identify specific clusters of PD symptoms depending on the non-motor phenotype, and it is interpretable at the level of presence of non-motor symptoms, with possible answers being “yes”, “no” and “don’t know” [70].

In a systematic assessment of its sensitivity and specificity, the NMSQ proved to be effective for screening most non-motor complaints, but for some (such as somnolence, olfactory loss and apathy), the sensitivity was suboptimal [74].

Limitations

To our knowledge, the NMSQ has not been used in disease-modifying trials in PD, and it lacks some reliability data on the original English version. Moreover, the questionnaire only scores presence or absence of non-motor symptoms and, therefore, does not inform about severity of specific symptoms or non-motor burden in general.

For those reasons, among others, it is less likely to be accepted by regulators than other more widely used non-motor scales.

Non-Motor Symptom Scale (NMSS)

The Non-Motor Symptom Scale (NMSS) is a rater-administered tool that comprises 30 items across nine non-motor domains: cardiovascular, sleep/fatigue, mood/cognition, perceptual problems, attention/memory, gastrointestinal, urinary, sexual function, and miscellaneous [75].

The NINDS-CDE version 2.0 has not included the NMSS, and has replaced it with its updated version, the Movement Disorder Society-sponsored Non-motor Rating Scale (MDS-NMS). However, it fulfils the criteria used in several MDS Critique and Review papers for classification as “Recommended” instrument [33]: it has been used in PD patients, validated, and applied by research groups beyond its original creators.

Strengths

The NMSS has been used in disease-modifying trials in PD, and in over 100 original research studies in PD [76]. The scale is clinically meaningful to patients and clinicians and is potentially acceptable to regulatory bodies. It provides information on presence, severity and frequency of specific non-motor symptoms (NMS) and of overall non-motor burden [75]. It is available through the MDS, although costs incur if it is used in clinical studies and trials. The NMSS is relevant for all PD patients and can be useful to identify specific PD clusters depending on the non-motor phenotype. The scale has negligible to no floor or ceiling effects, has good clinimetric properties in general, and correlates strongly with self-administered NMS questionnaire items [76]. The burden for both patients and clinicians is low, and it has an acceptable to good reliability [75].

Clinically meaningful cut-offs have been suggested for this scale, dividing the NMS burden into: no NMS (0 points), mild (1-20 points), moderate (21-40 points), severe (41-70 points) and very severe (71 points and higher) [76].

Limitations

Some domains of the scale group unrelated symptoms together, especially the "miscellaneous" domain, grouping weight change, hyperhidrosis, change in smell/taste and pain together, and hence making interpretation of the scoring of this domain difficult [76].

Furthermore, there might be floor effects in less prevalent symptoms of the NMSS, and it has low internal consistency for some domains, including perceptual problems (hallucinations) and sexual function. The NMSS has a low correlation with both disease duration and motor scores [76]. The latter is however not surprising as increasing evidence is showing that NMS have a non-linear progression and high NMS burden can already be present in early stages, or even prodromal stages, of the disease. The non-linear association with other scales might pose some difficulties, but as this appears to be inherent to NMS presentation in PD patients this is unlikely to be addressed by other holistic non-motor scales [76,77].

Movement Disorder Society-sponsored Non-motor Rating Scale (MDS-NMS)

The Movement Disorder Society-sponsored Non-motor Rating Scale (MDS-NMS) is the updated version of the NMSS, developed with the support of the International Parkinson and Movement Disorder Society (IPMDS) [78]. The scale has 52 items, grouped into 13 domains (depression, anxiety, apathy, psychosis, impulse control and related disorders, cognition, orthostatic hypotension, urinary, sexual, gastrointestinal,

sleep and wakefulness, pain, and other (unintentional weight loss, decreased smell, physical fatigue, mental fatigue, and excessive sweating)) [79]. Items are scored for frequency and severity, and then multiplied to obtain the total score [79].

Part of the MDS-NMS constitutes the Non-Motor Fluctuations (NMF) subscale. It has eight items related to these fluctuations: depression, anxiety, thinking or cognitive abilities, bladder symptoms, restlessness, pain, fatigue, and excessive sweating. It is scored for typical degree of change from “on” to “off” periods, and the sum of changes is multiplied by the amount of time spent in the “off” state with non-motor symptoms (‘’non-motor off’’)[79].

The NINDS-CDE version 2.0 has classified the MDS-NMS as Supplemental – Highly Recommended in PD.

Strengths

The MDS-NMS is available through the MDS, although costs incur if it is used in clinical studies and trials. Similar to the NMSS, it is clinically meaningful to both patients and clinicians, includes patient-reported outcomes, and implies a low burden for both parties, all of which make it a good candidate for approval by regulatory bodies. It is relevant for all PD patients and can be useful to identify specific PD clusters depending on the non-motor phenotype.

Regarding clinimetric properties, it has acceptable internal consistency (MDS-NMS and NMF total score’s average Cronbach’s alpha = 0.66 and 0.84, respectively); excellent interrater reliability (ICC >0.95); and test-retest reliability (ICC = 0.84 for MDS-NMS and 0.70 for NMF), and precision was found to be excellent for the MDS-NMS and fair for NMF [79].

Limitations

To the best of our knowledge, and probably due to its recent development, the MDS-NMS has not yet been included in disease-modifying trials in PD, but it is being used in other clinical trials.

Likewise, no solid data on sensitivity to change are available on this scale due to the lack of longitudinal cohort studies using the MDS-NMS, nor are there clinically meaningful cut-offs available yet. A moderate floor effect was present in patients for most MDS-MNS domains, with some components showing weak internal consistency, but there were no floor or ceiling effects of the total MDS-NMS score and its internal consistency was high. [79].

Scales for specific non-motor symptoms

Guided by the input of our Patient and Public Involvement and Engagement Working Group (PPIE WG) and by previous patient and carer input on the importance of different PD symptoms [32], we selected the NMS reported to be the most important (fatigue, pain, sleep, mood (depression, apathy, psychosis)) and looked into specific scales for these. As recently highlighted, there is no suitable scale for the screening of psychosis in PD, and a specific one should be implemented [80]. The other symptoms have recommended scales that are reviewed in more detail below.

Fatigue

Fatigue Severity Scale (FSS)

The Fatigue Severity Scale (FSS) includes 9 items involving motivation; physical function; responsibilities; work, family or social life; exercise; how easily fatigued; frequency of problems; and priority of symptoms. Each item is graded 1 to 7 and the total score is the mean of the 9 scores. It measures the interference of fatigue with daily life [81,82].

In its last critical review of fatigue scales, the FSS was “Recommended” by the MDS, both for screening and for severity rating [83].

The NINDS-CDE version 2.0 has classified the FSS as Supplemental in PD.

Strengths

The FSS has been used in disease-modifying trials in PD, and the burden of this scale on both patients and clinicians is low, since it is self-administered and takes approximately five minutes to complete [83]. As a patient-reported outcome, it is likely to be accepted by regulators. It gives information on the presence of fatigue and its functional impact [83]. The list of score items and the scale are available in the original FSS article [81], and although copyrighted, it is freely available from its developers [83].

In non-PD populations, floor or ceiling effects are low, the score distribution is normal, and it has a good association with the visual analogue scale (VAS) and various other fatigue rating scales, as well as with scales measuring related items (e.g., depression). The FSS has a high internal consistency (Cronbach’s alpha > 0.80), and there are acceptable item-total and intraclass correlation coefficients [82,83].

In PD, the FSS has good psychometric properties as well, and it has been widely used by different groups. Sensitivity to change has already been demonstrated in clinical trials.

A cut-off of 4 and a time frame covering the past 2 weeks have been frequently used by the developers and other groups [81], but other cut-offs have been suggested and used as well, such as a cut-off of 5 instead of 4 for high fatigue in the Norwegian version of the FSS [84].

Limitations

The FSS does not provide a definition of fatigue, and although extensive clinimetric testing is available for other conditions, this is relatively scarce in PD [83]. Furthermore, the MCID of the FSS in PD has not yet been established.

Pain

King's Parkinson's Pain Scale (KPPS)

The King's Parkinson's Pain Scale (King's PD Pain Scale, KPPS) is a rater-interview–based scale composed of 14 questions grouped into seven domains (musculoskeletal pain, chronic pain, fluctuation-related pain, nocturnal pain, orofacial pain, discolouration/swelling, and radicular pain), covering pain occurring over the last month, each item rated by severity and multiplied by frequency, the sum of which gives the total score [85].

In its critique paper on rating scales for pain, the MDS classified the KPPS as "Recommended" for rating pain intensity and "Suggested" for syndromic pain classification [86].

The NINDS-CDE has classified the KPPS as Supplemental in PD.

Strengths

The KPPS is widely available, easy to complete, provides patient-reported outcomes and has been used by multiple investigators [86], including in disease-modifying PD trials, which makes it likely acceptable to regulatory bodies. It provides information about the presence, severity, and frequency of pain. In addition, several key interventional pain trials have used the KPPS as an outcome [87].

Furthermore, the KPPS is the only pain scale with clinimetric assessment in PD [86]. There are clinimetric data on overall ratings of the KPSS, which show adequate internal consistency and inter-rater and test-retest reliability. Its validity is inferred by its moderate correlation with pain items from the EQ-5D-3L, the 8-Item Parkinson's Disease Questionnaire (PDQ-8), and the NMSS; and moderate/strong correlations with PD severity, quality of life and mood measures were also found [85,86].

The following cut-offs have been suggested for the Persian version of the KPPS: no pain (0 points), mild pain (1-17 points), moderate pain (18-68 points) and severe pain (69 or more points), with a sensitivity of 0.80 [88].

Limitations

The KPPS has significant floor effects [88], and due to the brief description of the different types of pain, it can be difficult for raters to classify a patient's description of pain, and rater training is warranted before administering the scale [86].

Sleep

The 2010 MDS review and critique paper on sleep rating scales, the following instruments were recommended: Parkinson's Disease Sleep Scale (PDSS), Pittsburgh sleep quality index (PSQI), the Scales for Outcomes in Parkinson's Disease – Sleep (SCOPA-Sleep), and the Epworth Sleepiness Scale (ESS) [89].

After the publication of this review, a revised version of the PDSS was validated: the PDSS-2 [90]. This scale, along with the ESS and the SCOPA-Sleep, are classified as Supplemental – Highly Recommended, by the NINDS-CDE version 2.0.

In this toolbox, we have incorporated a generic sleepiness screening tool, the ESS, and a PD-specific one, the PDSS-2. The latter is more widely used in PD studies than the SCOPA-Sleep (12 and 42 studies on clinicaltrials.gov as of October 12th 2022, respectively).

Parkinson's Disease Sleep Scale 2 (PDSS-2)

The Parkinson's Disease Sleep Scale 2 (PDSS-2) is the revised version of the PDSS. It is a patient-completed questionnaire with 16 questions on sleep-related items and 5 categories of possible responses for each question (Likert type scale), addressing the frequency of each item [90]. It includes important items not captured by the initial PDSS version, such as nocturnal akinesia, pain and sleep apnoea, and has a more accurate description on items related to restless leg syndrome (RLS) [91]. On the other hand, the PDSS-2 focused on nocturnal symptoms, and removed items like daytime sleepiness, which were present in the original PDSS [90,91].

Strengths

The PDSS-2 is widely available, has been used in trials of disease-modifying agents in PD, is brief and self-administered, and constitutes a patient-reported outcome.

It has been validated across the whole spectrum of PD severity and has shown satisfactory internal consistency and reproducibility. Regarding convergent validity, the PDSS-2 was closely correlated with the MOS sleep scale and with the PDQ-39 summary index [91].

As recently reported in a review [91], the PDSS-2 has negligible floor and ceiling effects, and it has also demonstrated good sensitivity [90] and responsiveness [92,93]. A clinically relevant cut-off of 18 points has been suggested for the PDSS-2 [94].

Although it was developed after the last MDS critique paper on sleep scales in PD, in which the PDSS was "Recommended", the PDSS-2 also fulfils the criteria to be classified as "Recommended" [89,91].

Limitations

The correlation of the PDSS-2 with the UPDRS sections III and IV and the Global Impression of severity was weak to moderate [91]. However, as both scales measure different domains of Parkinson's symptoms (motor symptoms with the UPDRS and sleep with the PDSS) this is less relevant.

Epworth Sleepiness Scale (ESS)

The Epworth Sleepiness Scale (ESS) is a screening tool for sleepiness developed for the general population. It consists of 8 items which reflect daily life situations each of them with a 4-point scale (0-3) to answer regarding the likelihood of falling asleep on each of them [95].

Strengths

It is widely available, brief (taking less than 5 minutes to complete), self-administered and hence a patient-reported outcome. There is broad experience in its usage, and it has been extensively translated into other languages.

Limitations

It only captures the tendency towards “falling asleep”, so drowsy or sleepy participants who do not fall asleep completely may score within the normal range; nor does it capture sleep attacks. For both reasons, in a PD population it would be best administered along with a PD-specific scale such as the PDSS-2.

Depression

In the latest MDS review of scales to assess depression in PD [96], no definite recommendations were made; the NINDS-CDE version 2.0 includes the Geriatric Depression Scale-15 (GDS-15) as Supplemental – Highly Recommended. However, a comparison of nine tools to assess depression in PD concluded that the GDS-30 may be the most efficient depression screening scale in PD [97].

We are aware of the complexity of depressive symptoms and the difficulty to capture it with a single scale. However, this OM toolbox does not aim to be a comprehensive review of measures to diagnose and monitor depressive symptoms (e.g., MADRS, CSDD, BDI, HAM-D, etc.), but rather a set of recommendations for suitable OM in each PD domain.

Geriatric Depression Scale (GDS)

The Geriatric Depression Scale-30 (GDS-30) is the 30-item version of the GDS [98]. It is a short, self-administered screening scale for depression originally developed for the elderly, but also used in PD [96]. Its main focus is psychological items rather than somatic ones, and each item is answered dichotomously (“yes”: 1 point/”no”: 0 points) [96,98].

The NINDS-CDE version 2.0 has not included the GDS-30 but the 15-item version of the GDS (GDS-15) is classified as Supplemental – Highly Recommended in PD.

Strengths

The GDS has been used in disease-modifying trials in PD, is widely available and lacks copyright protection [97], is brief (taking as little as 3 minutes to complete [97]), and self-administered, thus providing patient-reported outcomes and implying a low burden to both patients and clinicians.

It has excellent clinimetric properties, with sensitivity ranging from 0.72 to 0.81, specificity 0.82-0.85, positive predictive value (PPV) 0.58-0.84 and negative predictive value (NPV) 0.79-0.94, and a Cronbach's alpha value of 0.92 [97,99,100], and has also been shown to be sensitive to change in some studies [101].

In a review of depression scales for PD, a cut-off point of 10 or more points was determined as achieving an optimal sum of sensitivity and specificity, but the authors specified that this was not intended as a recommendation for clinical practice [97]. In this review, the GDS-30 was reported as having the best combination of clinimetric characteristics and easiness to complete [97]. Previous to that, a cut-off score of 9/10 was suggested, yielding a sensitivity of 0.809, a specificity of 0.837, a PPV of 0.584, and an NPV of 0.939 [100].

The GDS-30 is not affected by patient characteristics, which supports its use in all PD patients [97]. Although the GDS was validated on subjects age 55 and older [96], a study on potential effects of age on GDS-15 performance reported comparable validity of the scale in PD patients of all ages [102].

Limitations

Although initially devised for general elderly populations [98], there is growing evidence that the GDS-30 is a good rating scale for depression in PD patients of all ages [97,99,100].

9-item Patient Health Questionnaire (PHQ-9)

The 9-item Patient Health Questionnaire (PHQ-9) is the module of the Primary Care Evaluation of Mental Health Disorders dedicated to assess depression [103]. It scores each of the 9 criteria for depression included in the Fourth Edition of the Diagnostic and Statistical Manual of Mental Disorders (DSM-IV) from 0 to 3 according to frequency. It is classified as Supplemental in the second version of the NINDS-CDE.

Strengths

The PHQ-9 is a patient-reported outcome, and it is also brief (approximately 3 minutes to complete) and can be administered in person or remotely. It has already been used in disease-modifying PD trials [4].

Limitations

The PHQ-9 is not a PD-specific OM and its sensitivity to change is lower than other depression tools [104].

Columbia Suicide Severity Rating Scale (C-SSRS)

The Columbia Suicide Severity Rating Scale (C-SSRS) is not strictly a measure of depression, but one that assesses suicidal ideation and behaviours [105]. It is classified as Core as a general CDE recommendation and as Supplemental – Highly Recommended in PD in the second version of the NINDS-CDE.

The scale is administered as a clinical interview to detect suicidal ideation and suicidal behaviour. Four constructs are measured: severity of ideation (“severity subscale”, rated on a 5-point ordinal scale), intensity of ideation (“intensity subscale”, with 5 items, each rated on a 5-point ordinal scale), behaviour subscale (5-category nominal scale), and lethality subscale (6-point ordinal scale).

Strengths

The C-SSRS is a very brief (up to 5 minutes to complete) scale which can be administered remotely. It is clinician-administered tool, but it also has a self-report version. The C-SSRS has been extensively used and has excellent clinimetric properties: a publication reviewing the results of three multisite studies showed that the C-SSRS has convergent, divergent, and predictive validity; as well as good sensitivity to change; sensitivity and specificity [106]. In the same publication, the intensity subscale was reported to have good internal consistency.

Limitations

The C-SSRS is not a PD-specific measure. Training is recommended when the C-SSRS is used in academic research and usually required commercially sponsored trials.

Apathy

Apathy Scale (AS)

In a past review, the International Parkinson and Movement Disorder Society (MDS) Task Force recommended only the Apathy Scale (AS) to assess apathy in PD, although more recent reviews have shown other scales as potentially having better psychometric

properties, including the Lille Apathy Rating Scale (LARS) and the Starkstein Apathy Scale (SAS) [107–109]. The AS contains 14 items rated on a 4-point Likert scale (0 – not at all to 3 – a lot). Total scores can range from 0 to 42, with higher scores representing greater levels of apathy. The NINDS-CDE version 2.0 has included the AS and classifies it as Supplemental – Highly Recommended in PD.

Strengths

The AS is widely available and relatively brief to complete. It has been used in several studies aiming to determine its usefulness and capability in determining various aspects of apathy in PD patients [110–112]. Regarding its clinimetric properties, the AS has good internal consistency, interrater and test-retest reliability; and moderate item-total correlations. It seems that the AS can be decomposed into two factors reflecting Motivation-Interest-Energy on the one hand and Indifference symptoms on the other. These factors are differentially associated with clinical variables, including cognition and independent activities of daily living [112].

Limitations

As mentioned above, it seems likely that the AS can be decomposed into two factors, each associated with different aspects of apathy, indicating the importance of evaluating apathy from a multidimensional perspective and possibly limiting the use of AS as a comprehensive apathy scale in PD patients, despite its popularity.

Apathy Evaluation Scale (AES)

The Apathy Evaluation Scale (AES) [113] is a generic apathy scale, validated in *de novo* PD, PD with dementia and depression, PD with mild cognitive impairment (MCI)

and PD with deep brain stimulation of the subthalamic nucleus (DBS-STN). The rating varies in its 3 available versions: patient (self-reported, AES-S), clinician (AES-C), and informant (AES-I). It has 18 items and is one of the first instruments to quantitatively assess apathy in neurological populations [114].

In the latest MDS critique paper on apathy and anhedonia scales in PD, only the UPDRS and the Apathy Scale (AS) were Recommended, and the AES was only classified as ‘’suggested’’ because it lacked sufficient clinimetric testing in PD [109], but this has changed since then, hence now meeting criteria for “Recommended”.

The NINDS-CDE version 2.0 has not included the AES in its last classification, and classifies the AS Supplemental – Highly Recommended in PD.

Strengths

The AES-C is widely available, relatively brief to complete (20 minutes) [114], and adaptable to each individual situation, as it can be completed by clinicians, patients or informers.

It is currently being used [114], and has previously been used, in numerous PD studies.

Regarding its clinimetric properties, the AES-C has good internal consistency, interrater and test-retest reliability; and moderate item-total correlations [114]. Moreover, the AES-I and AES-P have good convergent validity [114]. The AES is the apathy scale with the highest sensitivity and specificity: both 90%, as reported in a validation study in *de novo* untreated PD patients [115].

Limitations

The AES-I and AES-P have a weak concurrent validity with the Neuropsychiatry inventory-apathy (NPIa) subscale [114].

There is scarce data on the AES' sensitivity to change and responsiveness to interventions in PD, although its high sensitivity and specificity are a good preamble. A longitudinal study on apathy and cognitive impairment in untreated newly-diagnosed PD patients showed stable mean overall AES scores, in line with the hypothesis that apathy is a persistent PD feature [101]. Nevertheless, when factoring in cut-off scores and clinical criteria for apathy, diagnosis of clinically relevant apathy changed over two years in several participants of the study, which supports the good sensitivity to change of the AES despite the persistency of apathy throughout the course of the condition [101].

Lille Apathy Rating Scale (LARS)

The LARS [116] is a PD-specific apathy scale, validated in a cohort with and without dementia. It includes 33 items grouped into 9 domains, to be answered either with a 5-point Likert scale or as "yes" or "no".

In the latest MDS critique paper on apathy and anhedonia scales in PD [109], the LARS was only classified as ''suggested'' because it had not been used in PD beyond its original developers, but this has changed since then, hence now meeting criteria for "Recommended".

The NINDS-CDE version 2.0 has classified the LARS as Supplemental – Highly Recommended in PD.

Strengths

The LARS has a favourable clinimetric profile (internal consistency, test–retest reliability, interrater reliability, and item-total correlations), and has shown good sensitivity and specificity.

Limitations

The LARS is longer to administer than other apathy scales, increasing the burden, although it can be completed in about 10 minutes.

Psychosis

The last MDS review on psychosis measurement instruments [117] concludes that no one scale can fully assess psychosis but, for clinical trials, recommended the Neuropsychiatric Inventory (NPI) (if cognitive impairment or if a caregiver is needed), the Scale for the Assessment of Positive Symptoms (SAPS), and the Positive and Negative Symptom Scales (PANSS). The authors also recommend combining a tool which measures clinical change over time (e.g., CGI-S) with a specific psychosis scale (e.g., NPI, SAPS).

The NINDS-CDE version 2.0 classifies the Scale for the Assessment of Positive Symptoms in PD (SAPS-PD (SAPS-PD) as Supplemental and its enhanced version (eSAPS-PD) as Supplemental – Highly Recommended.

Scale for the Assessment of Positive Symptoms in PD (SAPS-PD)

The SAPS-PD is a modified version of the SAPS, which was initially created to assess psychosis in schizophrenia [118]. The SAPS-PD has nine items (Auditory hallucinations, Voices conversing, Somatic or tactile hallucinations, Visual hallucinations, Global rating of severity of hallucinations, Persecutory delusions, Delusions of jealousy, Delusions of reference, and Global rating of severity of delusions) evaluated with a 6-point Likert scale (0-5) [119].

Strengths

The SAPS-PD maintains the reliability, sensitivity to change, and effect size of SAPS but is adapted to PD and briefer to administer [119]. It has been deemed effective as an outcome measure in clinical trials, and a clinically meaningful change has been established as 2.33 points (defined as a 1-unit change in clinical global impression).

Interestingly, the authors report no significant difference in scores between PD patients with and without cognitive impairment, although the authors established a Mini-Mental State Examination (MMSE) cut-off of 24 points and admit that below 21 points, there may be a change in symptoms between both populations.

Limitations

The SAPS-PD does not include some relevant items, such as illusions and olfactory hallucinations. It may not be suitable in cognitively impaired participants, although this trial will not include PD patients with dementia at the time of recruitment.

Enhanced Scale for the Assessment of Positive Symptoms in PD (eSAPS-PD)

The eSAPS-PD is a more comprehensive version of the SAPS-PD, which also includes questions about olfactory, gustatory, and minor hallucinations, as well as about less common delusions. It consists of and a global rating and 30 items in the following 4 domains: hallucinations (7 items), delusions (13 items), bizarre behaviour (5 items), and positive formal thought disorder (9-items) [120].

Strengths

The eSAPS-PD is very brief, taking 1-2 minutes to complete in asymptomatic individuals and up to 10 if symptoms are present [120]. It is the expansion of the

already validated SAPS-PD scale and includes items which are overlooked by that scale.

Limitations

To our knowledge, the eSAPS-PD has not been validated in PD nor used beyond their original developers, so further experience and formal validation would be warranted before recommending its use, despite its promising results in the original publication [120].

Autonomic dysfunction

There are two relatively recent MDS reviews of autonomic scales in PD. The earlier, in 2009, focused on sialorrhea, dysphagia, and constipation, but also included global scales and classified the Scales for Outcomes in PD-Autonomic (SCOPA-AUT) and the Non Motor Symptoms Questionnaire for PD (NMSQuest) as Recommended [41]. Another review in 2011, which focussed on orthostatic hypotension, also reported the SCOPA-AUT to be Recommended, and although the authors also classified the Composite Autonomic Symptom Scale (COMPASS) as Recommended, they mention that it had not been validated in a PD population and needed further assessment in this condition [121].

The NINDS-CDE version 2.0 include two autonomic scales: The Composite Autonomic Symptom Scale (COMPASS-31) as Supplemental – Highly Recommended, and the SCOPA-AUT as Supplemental.

Nevertheless, the SCOPA-AUT is a PD-specific autonomic scale and has been more widely used in PD studies than the COMPASS-31 (25 studies versus 3 studies in clinicaltrials.gov as of October 11th 2022, respectively).

For the above reasons, we have selected the SCOPA-AUT for consideration as a tool to measure autonomic dysfunction in our trial.

Scales for Outcomes in Parkinson's Disease – Autonomic Dysfunction (SCOPA-AUT)

The SCOPA-AUT [122] is a 25-item questionnaire which covers 6 domains: gastrointestinal (7 items), urinary (6), cardiovascular (3), thermoregulatory (4), pupillomotor (1), and sexual dysfunction (2 items for men and 2 items for women).

Strengths

It is a comprehensive, PD-specific scale, which correlates with disease severity, has good test-retest reliability, its total score did not show floor or ceiling effect in an independent validation study [123]. Although heterogeneous, it showed acceptable internal consistency, with Cronbach's alpha coefficients between 0.64 (cardiovascular and thermoregulatory subscales) to 0.95 (Sexual dysfunction in women).

Most importantly, it is highly correlated with specific HR-QoL and functional measures (r_S = 0.52–0.56) [123].

Limitations

The SCOPA-AUT has shown ceiling effects in some items (gastrointestinal, urinary), and includes some relatively non-specific items, such as swallowing, which may be impaired by causes other than autonomic dysfunction, including motor function. It lacks items such as fatigue, but this can be addressed by including one of the recommended fatigue-specific scales as suggested within the EJS ACT-PD initiative. In line with other comprehensive scales, the SCOPA-AUT is heterogeneous in its content, thus weakening its clinimetric properties.

Cognitive measures – Global

The 2018 MDS review and critique paper on cognitive scales in PD classified instruments as “Recommended” if they had been used in PD, by groups different than the creators of the scale, and if they underwent satisfactory clinimetric testing (reliability, validity, sensitivity to change). Accordingly, the review identified the following “Recommended” scales for cognitive screening in PD: Montreal Cognitive Assessment (MoCA), the Mattis Dementia Rating Scale Second Edition (DRS-2), and the Parkinson’s Disease-Cognitive Rating Scale (PD-CRS) [124].

Montreal Cognitive Assessment (MoCA)

The NINDS-CDE version 2.0 has classified the MoCA as Core in PD.

Strengths

The MoCA [125] has been widely used in PD research, including disease-modifying trials [126]. It is widely available, and although it requires training, it takes 10 minutes to complete, which means that the burden for both patients and clinicians is low.

It has good reliability with internal test-test accuracy, and inter-rater reliability [127]. It has been shown to be superior to the MMSE in detecting PD-MCI and PDD [128,129], with less of a ceiling effect.

Clinically meaningful cut-offs have been proposed of <26/30 [129,130] or <24 for PD-MCI [131], and <21/30 for PDD [129,131].

The MoCA has also proven to be sensitive to change [132,133], and can be used for all PD patients across the spectrum of cognitive impairment including those with PD-MCI and PDD. The MoCA has been proposed as the most useful instrument in PD-MCI, when compared with the MMSE, the Frontal Assessment Battery (FAB) and the Mattis Dementia Rating Scale [130].

It is classified as “Disease Supplemental – Highly Recommended” by the NINDS-CDE.

Limitations

The main limitation of the MoCA is that it is a measure of global cognition, with only limited sensitivity for each cognitive domain. Most patients score between 18-30, giving low variability in scores. This can limit sensitivity to detecting change over time, and in detecting relationships with other disease-related measures.

Mattis Dementia Rating Scale (DRS-2)

The Mattis Dementia Rating Scale-2 gives an overall scoring of cognitive function based on 5 subscales (attention, initiation/perseveration, construction, conceptualisation, and memory) [134].

It provides a minor refinement compared with the earlier version, with the tasks and stimuli unchanged, but allowing a wider age range; and a lower floor so that more impaired individuals can also be assessed.

The NINDS-CDE version 2.0 has classified the Mattis Dementia Rating Scale (MDRS) as Supplemental in PD. The authors explain that “although the MoCA and Mattis DRS-2 scored equally high on rating scale usage, only the MoCA is classified as “Core” given its more extensive use in PD and its significantly shorter administration time”.

Strengths

As with MoCA, the DRS-2 has already been used in PD disease-modifying trials [135].

It has good validity and reliability, and clinically meaningful cut-offs have been suggested: ≤ 132/144 for PDD; ≤ 139/144 or 140 for PD-MCI [134,136].

Furthermore, it has shown to be sensitive to change [137].

Limitations

The DRS-2 is copyrighted, and has costs associated with its use. It also requires training and takes 30 minutes to complete, which makes it slightly more inconvenient than the MoCA or MMSE.

Parkinson's Disease-Cognitive Rating Scale (PD-CRS)

The NINDS-CDE version 2.0 has classified the PD-CRS as Supplemental in PD.

Strengths

The PD-CRS is widely available, and although it requires training, it takes only 20 minutes to complete, making its burden for both patients and clinicians low/moderate.

It has shown good validity and reliability, and cut-offs have been suggested for PD-MCI (<81 [138]) and PDD (<62 or 64 [139,140]). Moreover, it is sensitive to change [138].

Limitations

The PD-CRS has already been used, to our knowledge, in at least one disease-modifying PD trial (MOVES-PD trial, NCT02906020), but in general it is used less widely in PD studies than the MoCA and the DRS-2 (clinicaltrials.gov search).

Addenbrooke’s Cognitive Examination-III (ACE-III)

The NINDS-CDE version 2.0 has classified the ACE-III as Supplemental in PD.

Strengths

The ACE-III is currently being used in disease-modifying trials in PD [141], is widely available, and although it requires training, it takes 20 minutes to complete, making its burden for both patients and clinicians low/moderate. It is composed of five cognitive domains (attention, memory, language, verbal fluency, and visuospatial abilities) and allows for generation of domain subscores.

The previous version of the ACE, the ACE-Revised (ACE-R), is reliable and has shown good validity for both PD-MCI [142] and for PDD [143,144]. It has also shown sensitivity to change [145] and clinically meaningful cut-offs have been suggested: <89 for PD-MCI [142,144]; <82 for dementia [146], <83 for PDD [144].

The ACE-R is no longer available for use due to copyright issues relating to inclusion of MMSE items within the scale. It has been replaced by the ACE-III, which substitutes the MMSE items for similar items. The ACE-III has been validated against the ACE-R and demonstrated high sensitivity and specificity in Alzheimer’s and frontotemporal dementia, with similar cut-offs as for the ACE-R [147]. Although the psychometric properties of the ACE-III have not been studied specifically in PD, its similarity with the ACE-R suggests that these can be inferred from ACE-R data.

Limitations

It is limited in assessment of executive functioning.

Alzheimer’s Disease Assessment Scale–Cognitive Subscale (ADAS-Cog)

The NINDS-CDE version 2.0 has classified the ADAS-Cog [148] as Supplemental in PD.

Strengths

The ADAS-Cog has already been used in several disease-modifying trials in PD, is widely available, and has shown good reliability and validity [149].

Limitations

The ADAS-Cog is copyrighted, and has costs associated with its use. It also requires training and takes 30 minutes to complete, which makes it more inconvenient than other shorter global cognition scales.

Furthermore, there is less data on the ADAS-Cog than on other cognitive scales in PD, and thus there is just limited information on its sensitivity to change and, to our knowledge, no clinically meaningful cut-offs.

Mini-Mental State Examination (MMSE)

The NINDS-CDE version 2.0 has classified the MMSE [150] as Supplemental in PD.

Strengths

The MMSE is one of the most commonly used cognitive instruments, and has been used in several disease-modifying trials in PD. It takes only 10 minutes to complete and has good reliability.

A cut-off of <26 points for PDD has been suggested [151].

Limitations

The MMSE is now copyrighted, has costs associated with its use (https://www.parinc.com/), and requires training.

Furthermore, shortcomings of the MMSE in PD include the lack of assessment of executive function and limited assessment of visuospatial abilities, both of which are commonly affected in PD.

There are mixed results on its validity, and is not sensitive in detecting mild impairment [152] or mild change [128].

Mini-Mental Parkinson (MMP)

The MMP was “Recommended with caveats” in the latest MDS review of cognitive scales [124].

The NINDS-CDE has not included the MMP in its last classification of outcome measures in PD.

This scale has been adapted from the MMSE and was designed for use in PD as a screening tool for cognitive change [153].

Strengths

It is quick to perform (15 minutes) and has good reliability [153], with a moderate correlation with the MMSE [154]. It is publicly available.

Limitations

The MMP lacks assessment of executive functions [124] and we lack information on how sensitive this scale is to change.

Scales for Outcomes in Parkinson’s Disease-Cognition (SCOPA-COG)

This scale was also designed as a PD-specific tool to assess cognition. It takes approximately 15 minutes to perform and is available in the public domain.

The SCOPA-COG was “Recommended with caveats” in the latest MDS review of cognitive scales [124].

The NINDS-CDE version 2.0 has classified the SCOPA-COG as Supplemental in PD.

Strengths

It is reliable and correlates with other widely used cognitive measures in PD [155].

Limitations

It has not yet been shown to have sensitivity to change, and in one longitudinal study, scores did not change over 4 years of follow-up [102]. It is more strongly weighted to memory rather than other cognitive domains.

Supplementary Table 2, adapted from the NINDS-CDE version 2.0 Cognitive Subgroup Summary document [156], summarises some of the most relevant cognitive scales.

Cognitive measures – Specific domains

A large number of assessment tools are available to assess specific domains of cognition which are of relevance in PD. However, the NINDS-CDE has not included any domain specific cognitive measures in its last classification of outcome measures in PD, and it is unlikely that inclusion of a comprehensive battery of domain-specific tests in a clinical trial protocol would be feasible given the time required for completion.

However, targeted testing of specific domains or tests may be considered, for domains that are known to be affected early in PD, or to have high predictive value for future cognitive decline. For example, verbal fluency (semantic) is quick to administer and has previously been shown to be highly predictive of future PD dementia [157]. Visuo-

spatial deficits are also emerging as predictive for cognitive decline [157–159]. These can be detected with tasks such as pentagon or cube copying, clock-drawing (as a 10-point test); the Judgement of Line Orientation [160], or Hooper's Visual Organisation Test [161], which are also not time consuming to perform. Tests of executive function such as the Trails test or the Stroop may also be considered, have low burden for both patients and clinicians, and are frequently impaired in early PD, but are less clearly predictive of later dementia.

Disability measures

In the MDS Critique paper on disability rating scales [162], these were classified as "Recommended" if they met the following criteria: to have been used in PD, to have been applied beyond their original developers, and to have undergone satisfactory clinimetric testing both in non-PD and in PD. In accordance to those criteria, the "Recommended" scales were: the Functional Status Questionnaire (FSQ), the Lawton-Brody Activities of Daily Living (LBADL), the Nottingham Activities of Daily Living (NEADL), the Schwab and England Activities of Daily Living (S&E ADL), the Self-Assessment PD Disability (SPDDS), the Short Parkinson's Evaluation or Scale/Scales for Outcomes in PD (SPES-SCOPA), the Unified PD Rating Scale (UPDRS)–II: Activities of Daily Living, the Movement Disorders Society UPDRS (MDS-UPDRS) Motor Experiences of Daily Living, and the PROMIS and Neuro-QoL Physical Function.

After searching on clinicaltrials.gov to estimate the frequency of use of each of those nine rating scales in PD studies, the three most commonly used as outcome measures in

PD studies, excluding the MDS-UPDRS part II and the UPDRS part II (already discussed above), were the FSQ, the Schwab and England ADL, and the PROMIS/Neuro-QoL. All three of them have been used in disease-modifying trials in PD, and therefore we have considered those in this toolbox of OM. However, we have classified the PROMIS/NeuroQoL under “HR-QoL: Generic” below.

Of note, in a recent systematic review of ADL measures in PD, only the MDS-UPDRS (either the full scale or part II alone) and the Schwab and England Activities of Daily Living Scale (S&E ADL) were recommended [163].

Schwab and England ADL Scale (S&E ADL)

The S&E ADL [164] measures the level of functional independence in 10 levels of ability to perform various chores, distributed in 10% intervals from 0% (“Bedridden”) to 100% (“Completely independent”) [162].

The NINDS-CDE version 2.0 has classified the S&E ADL as Supplemental – Highly Recommended in PD.

Strengths

The S&E ADL scale is widely available, brief to complete, and broadly used in PD, including in disease-modifying trials. It can be administered by clinicians, staff or be self-completed by patients [165].

This scale has high interrater reliability between patient and physician ratings (ICC = 0.82), good validity can be deducted from studies using the S&E ADL as a benchmark for other scales, and it has shown responsiveness to change over 1 and 4 years [162]. The S&E ADL scale has also shown to be remarkably responsive to change over time

[166] and regarding longitudinal validity, changes in this scale correlate with H&Y changes [167].

Limitations

At the time of the MDS review, there was a lack of direct assessments of the S&E ADL's validity, hence it being inferred from studies in which it was used as benchmark for other scales [162]. Nevertheless, test-retest (ICC = 0.70) and absolute reliability (standard error of the mean (SEM) = 4.45) were calculated for the S&E ADL after the MDS review was published [168].

Functional Status Questionnaire (FSQ)

The FSQ [169] is a self-administered questionnaire of six summary scales (basic and intermediate activities of daily life (ADLs), mental health, social activity, work performance, and quality of interaction), and six single-item scores. The items must be answered in either 4- (1-4) or 6-point (1-6) scales.

The FSQ is not included in the NINDS-CDE version 2.0.

Strengths

The FSQ has been validated in PD, with good internal consistency, (Cronbach's alpha = 0.92), content validity, and convergent validity in comparison to the UPDRS ADL, 36-item Short Form Health Survey (SF-36), and Schwab and England (S&E) scales [170]. More importantly for a disease-modifying trial, it has demonstrated sensitivity to change in both PD and non-PD populations [171,172]. A review of the reliability and validity of the FSQ in different non-PD populations also yielded good results [173].

Limitations

The FSQ was initially developed for primary care settings, and its use in disease-modifying trials in PD (e.g., ADAGIO follow-up study, NCT00936676) is less frequent than that of the PROMIS/NeuroQoL or the S&E ADL.

Capability measures

ICEpop CAPability measures (ICECAP)

The ICEpop CAPability measures (ICECAP) aim to capture broader wellbeing than health and focus on issues important to patients beyond their health-related quality of life, in order to allow more meaningful economic assessment of interventions that take place across health and social care. The ICECAP-A for adults [174] has five questions covering five dimensions: stability, attachment, achievement, autonomy, and enjoyment; and the ICECAP-O for older people [175], covers these five dimensions: attachment, security, role, enjoyment, and control. This suite of measures has scoring tariffs that have been developed using preferences, but the scores calculated using the tariffs are not utility weights and cannot be used to construct quality-adjusted life-years (QALYs) as there is no anchor of zero being equivalent to death, and this is a requirement for the construction of utility weights that are used to calculate QALYs.

Strengths

Brief and easy to complete. Provides an assessment of what is important to patients in terms of being able to enjoy life. The ICECAP-O in particular has previously been used in this and similar patient populations.

Limitations

Cannot be used in standard cost-utility analysis (i.e., involving QALYs) as the tariff preference weighting does not use the zero anchor at death so does not produce utility weights.

Carer burden measures

Carers quality-of-life questionnaire for parkinsonism (PQoL Carers)

The 26-item Parkinsonism Carers Quality of Life (PQoL Carers) is a self-completed questionnaire enquiring about various aspects of the wellbeing of people caring for people with parkinsonism (e.g., social activities, relationship to the patient, stress, mood), from which a summary index is calculated [176].

Strengths

It has high internal consistency (Cronbach's alpha = 0.96), as well as good convergent, concurrent, and discriminant validity [176], the latter being especially helpful in a disease-modifying trial, and has been shown to have good psychometric properties in Rasch analysis. It has been validated in carers of patients with parkinsonism [176] and it has been used in clinical trials in PD (personal communication, AS).

Limitations

This scale includes a question comparing the current level of health to that of 12 months ago, a relatively long timespan, which increases the risk of recall bias.

29-item Parkinson Disease Questionnaire for Carers (PDQ-Carer)

The 29-item Parkinson Disease Questionnaire for Carers (PDQ-Carer) is a self-completed questionnaire assessing the quality of life of carers of PwP. It covers four dimensions of quality of life (Social and Personal Activities, Anxiety and Depression, Self-care, and Strain) [177].

Strengths

In the original validation paper, it showed good internal consistency and reliability for all domains, as well as high data completeness and construct validity [177].

A single summary index score for all four domains has been proposed, with good internal reliability (alpha = 0.94) and valid, as shown by strong correlations with all domains of a generic health-related quality of life (HR-QoL) measure, the 36-Item Short Form Health Survey (SF-36) (all $p < 0.001$).

The PDQ-Carer has been used by other groups (e.g., it has been validated in Spanish [178]).

Limitations

The PDQ-Carer is relatively lengthy (29 questions), with which can be burdensome for the respondents.

To the best of our knowledge, it has not been used in disease-modifying PD trials.

Zarit Burden Interview (ZBI)

The ZBI is a 22-item questionnaire originally developed in 1980 to assess the burden on carers of people with dementia [179]. Since then, shorter versions have been developed, and its use has widened in a broad range of conditions, including PD [180].

Strengths

It has shown good clinimetric properties in PD: reliability (ordinal alpha 0.89—0.95), external construct validity, and corrected item-total correlations were ≥ 0.42, as well as negligible floor/ceiling effects (< 20%) except for the briefest forms.

The authors of the above-cited study suggest the 12- or 22-item forms for studies where carer burden is a central OM and the shorter versions when that is not the case [180].

Limitations

The briefest forms of the ZBI, namely the 4- and 1-item versions, have considerable floor effects (20% and 40% floor effects, respectively) [180].

The ZBI is not PD-specific, and to the best of our knowledge it has not been used in disease-modifying PD trials, although it has been included in a large number of symptomatic studies.

Health-related quality of life measures: Generic

In the MDS critique paper on health-related quality of life (HR-QoL) scales in PD [181], akin to other MDS critique papers, “Recommended” scales had to have been used in PD patients, beyond original developers, and have undergone successful clinimetric testing. In that review, HR-QoL measures were divided into generic and PD-specific.

The following generic HR-QoL scales are “Recommended”: EQ-5D, Nottingham Health Profile (NHP), 36-Item Short-Form Health Survey (SF-36), and Sickness Impact Profile (SIP). Of those, only EQ-5D and SF-36 have been used in disease-modifying trials in PD, according to our database. In addition, the MDS reviewed methods and

instruments for health economic studies of PD, and, regarding utility instruments, the EQ-5D and Health Utility Index instruments met criteria to be ”Recommended (with limitations)” and the EQ-5D was reported to have extensive data of performance on PD patients [182]. We also conducted a search in clinicaltrials.gov and found both to be widely used in PD studies. For all of the above reasons, this section includes the EQ-5D, SF-36 and HUI.

Please note that all the generic HRQoL measures can be used for both participants and their carers.

EQ-5D

The EQ-5D [183] is a measure of perceived health, constituted by 5 items with 3 response options (EQ-5D-3L) or 5 response options (EQ-5D-5L), and a visual analogue scale on the health status on the day of questionnaire completion, as perceived by the patient, from 0 to 100.

The NINDS-CDE version 2.0 has classified the EQ-5D as Supplemental – Highly Recommended in PD.

Strengths

The EQ-5D is one of the most widely used HR-QoL measures in research, including PD, and its clinimetric properties in this condition have also been studied. It is widely available and very brief.

It has adequate face/content validity, is responsive to interventions, highly reliable (good/excellent ICC), correlates with PD-specific (UPDRS) and general (SF-36) HR-QoL measures, and can differentiate PD stages [181,184].

The EQ-5D can be used for cost-utility analysis (i.e., calculating incremental cost per quality-adjusted life-year (QALY)) as there are tariffs to convert the responses into utility scores that can be used, if recorded across multiple timepoints, to calculate QALYs [185]. This type of analysis is often used in health technology assessment as it allows decision makers to consider allocation of health care resources across different disease areas that they might be responsible for, because QALYs are a generic outcome measure.

Limitations

No clinically meaningful cut-off is available for the EQ-5D in PD, and its brevity and it is not a PD-specific HR-QoL measure.

36-Item Short-Form Health Survey (SF-36)

The SF-36 [186] is a questionnaire including 36 items in 8 domains, and allows to summarise the results into physical and mental function.

The NINDS-CDE version 2.0 has classified the SF-36 as Supplemental – Highly Recommended in PD.

Strengths

The SF-36 is widely available, brief, self-completed, and has been extensively used in PD. It measures impact and change of quality of life compared to one year ago.

It has shown good reliability (Cronbach's alpha > 0.70) [187], Brown 2009), discriminative validity, and partial responsiveness to PD progression and interventions. In the aforementioned study, the responsiveness of the SF-36 was even higher than that

of two PD-specific HR-QoL scales (PDQ-39 and PDQUALIF) [187]. The minimal detectable change for the SF-36 has also been calculated [188].

A subset of responses to the SF-36 can be used to calculate utility scores via the SF-6D tariff [189], so it can be used to perform cost-utility analysis.

Limitations

Some subscales of the SF-36 have shown floor and ceiling effects, which would make it less suitable in very mild and very severe PD, and, to our knowledge, there is no evidence supporting the two physical and mental health components of the SF-36 in PD [181].

12-Item Short-Form Health Survey (SF-12)

The SF-12 is a shorter form of the SF-36, derived by it from its original developer [190].

Either of SF-12 or SF-36 could be used (via the SF-6D algorithm) [189] to calculate utilities and therefore QALYs.

The SF-12 is not included in the NINDS-CDE version 2.0.

Strengths

It is accessible, briefer than the SF-36, self-completed, and has shown good reliability and validity [190].

Limitations

It presents similar limitations to the SF-36, and additionally, it has less experience of use in PD trials than the SF-36.

Health utility index (HUI)

The HUI (versions I, II, and III) is another method to assess quality of life that also allows calculation of utilities [182,191,192].

The HUI is not included in the NINDS-CDE version 2.0.

Strengths

The HUI has been used in PD and there are data on its reliability and validity for a number of conditions [182]. The responses to relevant versions of the HUI can be converted into utility scores for cost-utility analysis.

Limitations

The HUI has not been validated in PD, and its use requires payment [182].

Patient-Reported Outcomes Measurement Information System (PROMIS)/Quality of Life in Neurological Disorders (Neuro-QoL)

The Patient-Reported Outcomes Measurement Information System (PROMIS) and Quality of Life in Neurological Disorders (Neuro-QoL) are two National Institutes of Health (NIH)–sponsored interrelated systems of patient-reported outcome measures [193,194].

They evaluate the ability of the responder to do a series of tasks, hence reflecting the physical, mental, and social consequences of neurological conditions. Since they are generic scales, comparisons can be made with other conditions [162]. The scoring is based on a T-distribution referenced to the U.S. general population where the mean is 50 and standard deviation is 10 [162].

The NINDS-CDE version 2.0 has classified the PROMIS-29 Profile and the PROMIS Item Bank v1.2 – Global Health as Supplemental in PD, and the Neuro-QoL as Supplemental – Highly Recommended in PD.

Strengths

The Neuro-QoL and PROMIS have been used in disease-modifying trials in PD, are widely available, clinically meaningful, brief, and easy to administer. Since they are PROs, they are good candidates in terms of acceptability to regulatory bodies.

Both have been validated [162], and have shown good validity to measure disability [195].

The reliability, validity, and sensitivity to change of both scales has been assessed.

The Neuro-QoL has been validated in PD, showing overall high reliability: good internal consistency (Cronbach's alpha = 0.81 to 0.94), test-retest reliability, and correlation with PD-specific measures, such as the UPDRS [162,195]. The PROMIS has been validated in population samples including PD patients [162] and is highly reliable as well, especially in advanced PD [196].

There are no clinically meaningful cut-offs for these scales, but in general, PROMIS' cut-off is established between 2 and 6 T-score points [197], and in the case of Neuro-QoL, a clinically Minimal Detectable Change can be calculated for its short forms [198].

Both scales have shown, to a greater or lesser extent, sensitivity to change. The Neuro-QoL has been reported to be responsive to self-reported change in the fatigue, mobility, positive emotion, and emotional/behavioural control domains [195]. Nevertheless, the only domain of the PROMIS that changed significantly for individuals with PD over 6 months in a study was Applied Cognition–General Concerns [196].

One of the greatest advantages of these measures is their adaptability, which ensures they are applicable to all PD patients: there are short forms, tailored forms for assistive devices [162], and PROMIS might be especially useful in advanced PD and in the case of caregivers completing it [196].

They both provide information on the presence and impact of disability in PD.

Limitations

None of these scales are PD-specific measures, and, to our knowledge, PROMIS has not been validated in a PD population exclusively, apart from the sleep disturbance (PROMIS-SD) and sleep-related impairment (PROMIS-SRI) tools [199].

Health-related quality of life measures: PD-specific

In the MDS critique paper on HR-QoL scales in PD [181], the following PD-specific health-related quality of life (HR-QoL) scales are “Recommended”: Parkinson’s Disease Questionnaire (PDQ-39), Parkinson’s Disease Questionnaire Short Form (PDQ-8), Parkinson’s Disease Quality of Life Questionnaire (PDQL), Parkinson’s Impact Scale (PIMS), and Scales for Outcomes in Parkinson’s Disease–Psychosocial (SCOPA-PS). Of those, only PDQ-39, PDQ-8 and PDQL have been used in disease-modifying trials in PD, according to our database. We also conducted a search in clinicaltrials.gov and found the PDQ-39 and, to a lesser extent, the PDQ-8 to be widely used in PD studies.

Moreover, in a recent systematic review of tools to measure HR-QoL in PD, the two most common questionnaires were the PDQ-39 and the PDQ-8 [200].

For all the above reasons, this section includes the PDQ-39 and its shorter form, the PDQ-8.

39-item Parkinson's Disease Questionnaire (PDQ-39)

The PDQ-39 [201] is a HR-QoL instrument comprising 39-questions on frequency of different PD-related issues on daily life, with 5 possible answers for each, and grouped in 8 subscales. Global HR-QoL can be estimated from the summary index of the PDQ-39 (PDQ-39 SI) [202].

The NINDS-CDE version 2.0 has classified the PDQ-39 as Supplemental – Highly Recommended in PD.

Strengths

The PDQ-39 is broadly used and has been translated and validated in various countries [181]. It is widely available, self-completed (minimal clinician burden), and the PDQ-39 SI has shown excellent clinimetric properties: satisfactory content and convergent validity, internal consistency and stability, and no relevant floor and ceiling effects. The Cronbach's alpha of the total PDQ-39 scale in different studies ranges from 0.84 to 0.94 [203].

Both discriminative validity for different PD stages and interpretability parameters have been defined for the PDQ-39 SI [181].

The minimal clinically relevant change has been calculated to be 1.6 points [204]; and a clinically meaningful change of 3.5 points has also been suggested [205].

The PDQ-39 is sensitive to change. In fact, changes in it have been reported to corresponds to clinicians' judgements of change over time in a prospective observational study of patients attending neurology clinics [206], and it also produces statistically significant changes for the majority of domains for which PD patients reported deterioration [207].

A subset of responses to the PDQ-39 (i.e., those in the PDQ-8) can be mapped to the EQ-5D-3L for calculation of utility scores, for use in cost-utility analysis, although this is less preferred than using a direct measure (e.g., EQ-5D-5L) and avoiding mapping [208].

Limitations

The PDQ-39 lacks some relevant HR-QoL areas, such as sleep and sexual function [181].

Furthermore, although overall an excellent HR-QoL scale in PD, the PDQ-39 is lengthier than other scales, which might represent a burden to some patients. Thus, a shorter version of the PDQ-39 has been developed: the PDQ-8 [202].

8-item Parkinson's Disease Questionnaire (PDQ-8)

The PDQ-8 [202] is the short version of the PDQ-39, and contains 8 items representing each of the 8 different domains in the PDQ-39.

The NINDS-CDE version 2.0 does not include the PDQ-8.

Strengths

The PDQ-8 is widely available (although applying for a license is necessary: https://process.innovation.ox.ac.uk/clinical), briefer than the PDQ-39, reducing patient's burden, and can be used in health-economic analysis.

Regarding psychometric properties, the PDQ-8 has shown satisfactory internal consistency, item–total correlation, test–retest reliability, and convergent validity, as well as responsiveness to interventions and sensitivity to change [181,209]. No floor or ceiling effects have been identified, and a minimal important difference (MID) has been

calculated (MID values ranged from 5.8 to 7.4 points, but should be taken as preliminary estimates [209]). Responses to the PDQ-8 can be mapped to the EQ-5D-3L for calculation of utility scores, for use in cost-utility analysis, although this is less preferred than using a direct measure (e.g., EQ-5D-3L or 5L) and avoiding mapping [208].

Limitations

Probably due to its simpler content, the PDQ-8 has shown lower reliability (Cronbach's alpha = 0.80) [210] and validity than the PDQ-39 [181].

Health economics – Resource use

Costs of treatment pathways are generally calculated as the sum of resource use items multiplied by their unit cost, with exact decisions about which costs to include being based on the required perspective for the analysis, e.g., national health service perspective, or wider perspective, for example including social care, patients' and/or carers' out-of-pocket costs, and productivity losses. Capture of information on resource use is commonly performed using patient-reported questionnaires including the Client Service Receipt Inventory (CSRI) [211–213], supplemented by information from electronic health records where appropriate, to both improve quality and reduce burden on participants.

Client Service Receipt Inventory (CSRI)

Strengths

The CSRI is free and no registration is required. Any type of data can be collected, including social care, education support, time off work and other non-health information. The CSRI requires modification by the health economist before use with input from PPIE members, clinicians, carers, and any other stakeholders to tailor it to the specific context. This means that patient and clinician burden in completing the questionnaire can be minimised, and relevance/applicability can be maximised.

Limitations

The burden in the case of patients and carers is medium due to potentially lengthy assessments (although this is tailored to the specific needs of the study as mentioned), and minimisation of the gathered data can be done to reduce the burden. Data completion rates can be variable.

Electronic health records

Strengths

Using electronic health records reduces burden for participants, and often leads to more complete data capture and therefore less bias. Time periods before the trial start date and after follow-up has finished can also be covered.

Limitations

Most routinely available electronic records were not designed for research use, rather they were designed for auditing health services, or for reimbursement of providers of services, and this can mean that the data are not always straight forward to cost, and that not all items of interest are captured. Costs for obtaining datasets can also be high, in terms of paying directly for access to datasets and the researcher time required for

making the application (e.g., NHS Digital), or researcher time required to go in to services and retrieve data.

Health economics – Cost-utility analysis

Health economics analysis requires information on both quality of life and costs. Quality of life information should be captured such that responses to appropriate questionnaires can be converted into utility scores, and over a number of time points so that QALYs over the relevant time period can be calculated for cost-utility analysis. Costs depend on the decision maker's requirements but generally include resources associated with treatment and care pathways. Costs are calculated by applying appropriate unit costs to resource use information, captured for example using the CSRI and/or electronic health records.

Appropriate approaches would be to use QALYs calculated from repeated measures of a generic HR-QoL measure such as the EQ-5D-5L as the primary cost-utility analysis, with secondary analyses using QALYs calculated by mapping from other HR-QoL measures described above (e.g., PDQ-8 [208]). The cost component of the analysis would be calculated as the treatment and care pathway costs in the arms to be compared, from the perspective of the National Health Service (NHS) and Personal Social Services (PSS), which is the approach recommended by NICE [214], using a combination of a tailored CSRI and electronic health records where possible.

Milestone-based outcome measures

In recent years, there has been a change of paradigm on clinical measures in PD, especially in disease-modifying trials, due to the challenges they purport, such as the need for greater sample sizes, and the potential masking effect of symptomatic therapy. Its progressive nature has led to the development and application of milestone-based outcome measures, which determine the time until a relevant situation in the course of the disease is reached. Milestones used in the literature include the time to levodopa therapy [215], dyskinesia onset, postural instability, dementia and death [24]. More recently the concept of using "emergent symptoms" as a milestone has been proposed. [216].

One of the most recent examples is derived from the MDS-UPDRS, and the authors analysed data from the STEADY-PD3 study to detect emergent symptoms on the patient-reported non-motor (MDS-UPDRS part IB) and motor (MDS-UPDRS part II) experiences of daily living in participants who started symptomatic treatment and those who did not, on years 1 and 2 of the study. In this study, emergent symptoms in part II were more frequent in the group starting symptomatic treatment [216].

Milestone-based OM have shown various advantages so far, such as potentially requiring smaller sample sizes due to accrual [216], and furthermore, they have the potential to overcome the masking effect of symptomatic medication when assessing a putative disease-modifying intervention. This was initially reinforced by researching "dopa-resistant" milestones (i.e., focused on symptoms less responsive to dopaminergic therapy: axial symptoms, cognition, etc.), but recent studies show that time-to-event approaches involving MDS-UPDRS part III can also be promising outcome measures, especially when combined with known minimal clinically important differences [217]. Importantly, clinical milestones have shown to be related to PD mortality, and to have a cumulative effect on it [218].

Digital measures of individual motor signs and function

All digital health OM are still considered Exploratory in the NINDS-CDE version 2.0.

Digital measures of motor signs or function that can be acquired at higher-frequency than clinic-based measures, providing the opportunity to capture more than a snapshot of disease burden compared with traditional clinic-based measures. Expert-rater measures such as the MDS-UPDRS III, are generally time-consuming and so more difficult to perform frequently, more expensive to administer and subject to greater inter and intra-rater variability. Digital technology can help surmount these difficulties, ensuring that selected PD outcomes are measured in a more realistic manner objectively, more frequently, at home and on an individual level. Despite these potential advantages, the exact value of measurement properties of many motor digital measurements remains unclear, and there is lack of clarity regarding the exact criteria a digital trial endpoint should fulfil. Here, we chose to divide digital health measures into (i) active only testing- requiring the individual to actively perform scheduled tasks with the digital device (e.g., smartphone, actiwatch), (ii) passive only testing–pre-programmed to be worn for specific purposes over prescribed time points (e.g., Parkinson's Personal KinetiGraph® (formerly Parkinson’s KinetiGraph®) (PKG), Axivity sensor device, and (iii) combined active and passive testing (e.g., combined Roche smartphone app and wrist-worn sensor).

Active only digital testing measures

OPDC (Oxford’s Parkinson’s Disease Centre) Smartphone app

This app provides a measure of overall motor function: voice, standing balance, gait, reaction time, finger tapping, rest and postural tremor tasks – all combined to give single motor composite score for right and left sides. The test comprises 7 tasks, of which 4 are bilaterally performed, giving right and left scores in addition to mean motor score. It was designed as a diagnostic marker [219] but is currently undergoing evaluation as a marker of PD and prodromal PD progression.

Strengths

It is currently being used in disease-modifying trials in PD (Exenatide-PD3 trial), and its burden on patients and clinicians is low: it requires 20 minutes of trainer time to administer it in clinic or at home, and the total test takes 8 minutes to complete. It is also available at no cost as a research collaboration with OPDC, and is available in 13 languages.

In a study of > 900 PD patients, the smartphone-predicted motor composite scores showed high correlations (absolute r=0.70-0.90) with MDS-UPDRS parts II, III, Purdue Pegboard Test and Timed Up and Go (TUG) clinical tests (unpublished data, in preparation). Test-retest reliability was found to be excellent (ICC 0.84 in PD (n=111, unpublished data in preparation). This indicates that the smartphone-predicted composite motor score has relevance to clinician and patient-rated quality of life scores.

This measure is aimed at a broad range of PD patients, including prodromal PD (RBD) patients, compared with age- and gender-matched control participants. It informs on the severity of the motor impairment, and might be potentially a diagnostic tool [220].

Limitations

In terms of regulatory status, the OPDC smartphone app is not currently listed as a medical device, and no data on its clinical relevance as a prognostic marker or reliability are currently published.

Future publications covering this area focusing on score metrics, and utility to measure motor progression in manifest PD and prodromal subjects with RBD are expected soon.

CloudUPDRS smartphone-based measures of limb-specific tremor/bradykinesia

This tool makes individual MDS-UPDRS III subscore predictions derived from smartphone sensor data during 16 bilateral upper and lower limb active assessments including finger-tapping, pronation/supination, leg agility, and resting/postural/intention tremor.

Strengths

The cloudUPDRS has been CE marked as a class 1a medical device, and has self-guided instructions. It takes 15 minutes to complete one full test, which means the burden on patients is likely only low, and the burden on clinicians is minimal.

It has been cross-validated against 3 blinded expert raters in 60 PD patients, and its out-of-sample individualised prediction accuracy for subscores of MDS-UPDRS-III was 53.2-97% [221].

The predicted MDS-UPDRS part III subscores will have similar meaning, sensitivity to change and cut-offs as part III of the clinical scale. The underlying latent digital score should have increased sensitivity to change, but this has not been empirically quantified.

Limitations

This tool does not provide a composite score and has not been used yet in PD disease-modifying trials. Its reliability has not yet been measured (although its software has been designed to be relatively consistent across a range of modern smartphones). Furthermore, as with the MDS-UPDRS part III, it is moderately meaningful to clinicians but not to patients.

Feasibility caveats include the fact that it is an active measure, the need to have access to a smartphone, and the fact that is only available on Android OS, and only in English. It has not been tested in PD patients without dementia.

Mobility lab system (APDM)-measures acquired typically in controlled settings

These measures usually assess bradykinesia, dyskinesia, gait, and postural control.

They are possibly acceptable to regulators, since it has already been included in disease-modifying trials, but has some limitations, especially in terms of feasibility and due to its lack of patient-reported outcomes (purely motor assessment).

Strengths

These measures have already been used in disease-modifying trials in PD, are reliable (ICC’s 0.55-0.84), moderately meaningful to clinicians and have a good correlation with MDS-UPDRS III (r=0.73). It is important to bear in mind that not all of these measures aim to replicate the MDS-UPDRS, since they are highly specific and the MDS-UPDRS is heterogeneous, so depending on the measure, a strong correlation with that scale is not always expected. They have also shown a moderate-good correlation with the PIGD subscore of MDS-UPDRS III [222,223].

Limitations

Although their cost is only moderate, their main limitation is that they are used in controlled settings due to limited battery life. This, among others, makes them moderately burdensome to patients and clinicians.

mPower smartphone-derived composite (dominantly motor) impairment score

This is a single composite motor scope derived from smartphone sensor data during 5 tasks: tapping, voice, walking, balance, memory.

Strengths

This score has a low burden on patients (it takes less than 5 minutes to complete) and clinician burden is unknown but likely minimal.

This score correlates with MDS-UPDRS part III (r=0.88) and with the Timed Up and Go (TUG) test (r=0.72) [224]. It has also shown to be responsive to interventions, as treatment with levodopa increased the score by mean of 16.3 [224].

Limitations

The mPDS has not been used in disease-modifying trials in PD yet. Its cross-platform feasibility is unclear, it is not a medical device, there are no data on its reliability and usefulness in specific PD subgroups, and no clinically meaningful cut-offs have been suggested. Moreover, its clinical meaning to both patients and clinicians is not well established. In addition to that, it is an active measure, and requires access to a smartphone. This score is only available on Android OS, only in English, and is not a medical device. HopkinsPD smartphone-derived composite (motor dominant) impairment score (mPDS score).

Passive only digital testing measures

Parkinson's Personal KinetiGraph® (formerly Parkinson’s KinetiGraph®) (PKG)-based proxy measures of whole-body tremor/bradykinesia/dyskinesia

These are high-frequency measures of ‘whole-body’ bradykinesia, tremor and dyskinesia inferred from continuous unilateral wrist-worn sensor data.

Strengths

PKG-based measures are passive measures, which reduces both patient and clinician burden, as the patient only needs j wear the device for one week. Regarding regulation, they are CE and FDA approved.

Motor signs are split into meaningful parameters for clinicians, but sign magnitude is less interpretable. Its reliability is unknown but it is in widespread use and many clinicians have built-up familiarity with it.

A validation of the algorithm was conducted 34 PD patients and 10 age-matched controls, and the mean Bradykinesia score and UPDRS part III correlated with r=0.64 [225].

This is a familiar output to clinicians, although some algorithms are partially proprietary.

Limitations

To the best of our knowledge, PKG-based proxy measures have not yet been used in disease-modifying trials in PD, and the motor signs measured are not meaningful to patients.

It also presents some feasibility challenges: the proprietary hardware and software are needed, and despite the low patient and clinician burden, an expert review of data post-acquisition is required. Furthermore, although reliability data are expected from the proprietary hardware, the inter-rater reliability of semi-supervised is unknown.

Its variability across patient subgroups is unknown and although it is clearly sensitive to change, the specific sensitivity is not known.

Motor fluctuations Monitor for Parkinson's Disease (MM4D)-based proxy measures of whole-body tremor/dyskinesia

High-frequency measures of 'whole-body' tremor and dyskinesia inferred from continuous unilateral wrist-worn sensor data.

Strengths

Similar to the PKG, the MMM4D provides passive measures (patients have to wear the device for a week), and the motor signs are split into meaningful parameters for clinicians.

Its reliability is unknown but proprietary hardware is likely to give reliable results. Regarding validity, a study in 118 PD patients reported that within-sample tremor amplitude correlates with MDS-UPDRS part III ratings (Rank correlation coefficient 0.8), although its validity in dyskinesia was less clear, but the false positive detection was 2-61% depending on activity [226].

This is a familiar output to clinicians, although some algorithms are partially proprietary.

Limitations

To the best of our knowledge, the MM4D has not yet been used in disease-modifying trials in PD. Similar to other digital measures, the specialist proprietary hardware (Apple Watch) and software are needed, and it is only available on Apple OS, and in English. Furthermore, although reliability data is expected from the proprietary hardware, the inter-rater reliability of semi-supervised data collection is unknown.

As with the PKG, the motor signs measured are not meaningful to patients.

Axivity (AX3 & AX6) gait accelerometer

These tools measure laboratory and free-living gait, falls and ADLs over 7-21 days.

Strengths

The Axivity gait accelerometers are widely available at a low cost, with a low burden to patients and clinicians, good to moderate reliability (ICC's = 0.913-0.5), and a good association with history of falls and physical activity [227–229].

This measure is useful in all patients with PD and in subgroups, and provides proxy measures of severity (e.g., motor measures such as gait) which are well interpretable.

Limitations

Whilst we are aware that the Axivity gait accelerometers are being used in a number of clinical studies, no data in disease-modifying trials in PD have been reported.

Combined active and passive digital testing measures

Roche smartphone app

The second version of this app (V2) provides a combination of active and passive testing, with 10 active smartphone-administered tasks (shape drawing finger tapping, pronation/supination, speech, phonation, postural and rest tremor, standing balance, gait with U-turn and cognitive testing) and 3 passive monitoring tasks using a wrist-worn sensor that include gait, arm swing, tremor and mobility [230], In the recently published Phase II Pasadena study [230] using V2, a provisional, single summary sensor-based measure 'the PASADENA Digital Motor Score' was developed and used as an exploratory end point, reflecting global motor function.

Strengths

It has been used in several Roche-sponsored PD disease-modifying trials, including the Phase I prasinezumab PD study, in which preliminary reliability and clinical validity were established [231]; showing 82% adherence with all possible active tests performed around 2 weeks baseline. Test-retest reliabilities were good, with moderate to excellent test-retest reliability (average intraclass coefficient =0.84) [232]; and good associations with individual components of the MDS-UPDRS III (Spearman's rho -0.23-0.63) [231].

Limitations

The burden of this assessment on both patients and clinicians is unclear, although likely higher for the former in V2, which has an increased number of tests.

Likewise, the interpretability of the Roche app is not clear, and individual subscores may improve interpretability if supplied.

Other Digital/Timed motor measures

These include a number of measures assessing gait, balance, activity, turning, tremor, and dyskinesia. For a review of state of the art on this field please refer to Del Din et al., 2021 [229].

Strengths

These measures are feasible, and there are systems in place that can take those measurements, some commercial and some solely for research use (e.g., McRoberts Move Monitor, Axivity AX6).

Mobility related measures, such as Digital Mobility Outcomes are clinically meaningful, and these measures in general seem to be valid in terms of measuring what they aim to measure [228].

The burden on patients and clinicians depends on the system but in general, it should be relatively low if data are passively captured, which also increases compliance. On the other hand, the need of some measures to be obtained through patient interaction increases the burden on them.

In the case of Mobilise-D, work is in progress to produce reliability data from free-living, and early evidence suggests accuracy of measurement. The technical and clinical properties are currently being validated in a large consortia effort with the objective of regulatory approval (e.g., Mobilise-D – https://www.mobilise-d.eu/) [233].

Limitations

These measures have not been used in disease-modifying trials in PD yet. In fact, the Verily watch application to the FDA was recently rejected, as they are looking for measures that capture meaningful aspects of health.

As already mentioned, UPDRS motor-related measures are not clinically meaningful and are therefore less likely to be approved by regulatory bodies.

No clinically meaningful cut-offs have been established for these measures.

Quantitative motor measures

None of the below instruments has been included in the NINDS-CDE version 2.0.

Timed up and Go test (TUG) 3 metre

This test measures the time needed to stand from a chair, walk 3 metres, turn around, return to the chair and sit back down [234].

Strengths

The TUG has already been used in disease-modifying trials in PD. It is widely available, requires minimal training and has no cost. Consequently, the burden on both patients and clinicians is low.

It has shown good to excellent test-retest and intra-rater reliability (ICC 0.87-0.99), and has a good correlation with the mini-BESTest, the Berg Balance sale, and the Postural instability and gait difficulty scale.

A cut-off point of >2.2 seconds offered an 85% sensitivity and a 100% specificity [235].

The TUG assesses the severity of motor impairment, and is relevant for all PD patients.

Limitations

The TUG has poor sensitivity to change, and a study reported an effect size of 0.16 [236].

Purdue Pegboard test

This test evaluates manual dexterity and, to a lesser degree, attention and executive cognitive ability [237].

Strengths

The Purdue Pegboard test has already been used in disease-modifying trials in PD. It is widely available at a minimal cost and in terms of clinical meaning, it has shown a moderate association with self-reported manual dexterity. The burden of the Purdue test on both patients and clinicians is low.

In terms of reliability, it has shown moderate to good test-retest reliability, good inter-rater ability, moderate correlation with the Manual Ability Measure-36 (MAM-36) questionnaire score, and adequate construct validity to assess mild to moderately severe PD in on, but not at the end of dose [238]. It appears to be more sensitive to PD-related changes in dexterity than the 9-hole peg test [238]. It has been shown to be a sensitive predictor of prodromal PD conversion in RBD patients [239] and it is applicable to all PD and RBD patients. This test could also be potentially relevant in PD subgroups with cognitive impairment or at risk for it, since it has been shown to predict the development of dementia in PD [240].

Limitations

Limited data exists on the sensitivity of this test to detect change in PD patients.

Alternate tap test

This test looks at the patient's manual dexterity by assessing the motor speed in the hands [241] (Nutt et al., 2000). In short, it quantifies the number of times a single hand

taps alternately two counters separated by a fixed distance and during a fixed time interval [242]. The original tap test consisted of two mounted counters separated by 5 inches which had to be tapped as many times and as accurately as possible over a 30-second period [243].

Strengths

The alternate tap test is widely available at a minimal cost, it has shown a moderate association with self-reported manual dexterity, and its burden on both patients and clinicians is low. It assesses the severity of the manual dexterity impairment. Similarly to the Purdue pegboard test, the alternate tap test has shown to predict the development of dementia in PD [240].

A threshold between 10% and 20% increments in the tapping test yielded a 100% sensitivity and specificity to detect change in maximal velocity during acute drug challenges [242].

This test correlates to the bradykinetic component of motor slowness in PD [242], as well as with other parameters such as walking speed [242].

Limitations

Tapping speed is inversely related to age, and this needs to be considered when interpreting results in a heterogeneous population [241]. Furthermore, there is a practice effect associated with this test, which needs to be factored in especially if repeated measures are needed during the same assessment [241]. The tapping test is strongly influenced by on and off motor states [244] which might impair its suitability to detect disease modification.

To the best of our knowledge, the alternate tap test has not been used in disease-modifying trials in PD yet.

Bradykinesia Akinesia Incoordination Tap Test (BRAIN test)

The BRAIN test is an alternate tapping task administered online using a standard computer: the subject is asked to tap the “S” and “;” keys as quickly and precisely as possible during a 30-second time period [245]. It replicates the traditional alternate tap test.

Strengths

It is freely available online, objective, and very brief, consequently implying a low burden. Apart from having shown good clinimetric properties, it can monitor motor function longitudinally and has been used in observational and interventional studies both of manifest and of at-risk PD populations [246].

Like other tapping tests, it is important to factor in the participant’s age when interpreting the results of the BRAIN test [246].

Limitations

As with any measures involving technology, there can be barriers to participants less familiar with it. The BRAIN test was developed to assess the effect of symptomatic treatment in motor PD function, so its suitability to detect disease modification is still to be elucidated. To the best of our knowledge, results from use of the BRAIN test in disease-modifying trials in PD have not been published.

9-hole peg test (9hpt)

This test assesses upper limb function by requesting subjects to pick up 9 small pegs from a holding well, place them into holes on a board, and then move them back to the well, all with one hand, as quickly as possible [247].

Strengths

As it was the case with the previous two tests, the 9hpt is widely available at a minimal cost, and its burden on both patients and clinicians is low. It assesses the severity of the manual dexterity impairment, and it is applicable to all PD and RBD patients. It is a standardised test with available normative values for healthy controls across ages [247].

Regarding its clinimetric properties, it has shown moderate to good test-retest reliability, good inter-rater reliability, moderate correlation with MAM-36 score, and adequate construct validity to assess mild to moderately severe PD in on, but not at the end of dose [238,247]. There is also support for its construct validity to assess the performance of the more affected hand in mild to severe PD [248].

The 9hpt has already been used in PD disease-modifying trials (e.g., N-acetylcysteine, NCT01470027).

Limitations

The 9hpt is less sensitive to PD-related changes in dexterity than the Purdue pegboard test [238].

Composite Quantitative motor measures

Similar to the previous section, none of the below instruments has been included in the NINDS-CDE version 2.0.

OPDC Oxford Parkinson's Disease Centre (OPDC) composite clinical score

The OPDC composite clinical score combines the MDS-UPDRS part III, the Purdue Pegboard, and the TUG test scores using PCA approach, to obtain a single semi-quantitative composite score derived from principle component analysis of those three assessments [249].

Strengths

This score is now widely available through an online publication and composite score calculator [249] and it has shown significant correlations with the EQ-5D-3L visual analogue scale and the MDS-UPDRS part II. Clinical trial modelling [249] shows that the use of the smartphone composite motor score as primary study output requires a smaller sample size than the MDS-UPDRS part III for the same 50% effect size.

Its reliability is good, as shown by the lower coefficient of variation with the composite clinical score (37%) than with the MDS-UPDRS part III (67%) [249]. Furthermore, greater magnitude of change over time has been reported with the composite clinical motor score compared with MDS-UPDRS III scores [249]. It is useful for all PD and also for prodromal PD with RBD sleep disorder and informs of the severity of symptoms.

Limitations

The OPDC composite clinical score has not been used in disease-modifying trials in PD yet. Its burden on both patients and clinicians is moderate, as it requires 1 hour of in-person testing.

Molecular neuroimaging

An extensive review on neuroimaging techniques in PD was recently published [250]. Here, we will focus on the suitability of molecular techniques as outcome measures for PD disease-modifying trials.

The NINDS-CDE version 2.0 has classified PET-SPECT Localization as Supplemental – Highly Recommended and Supplemental.

Dopaminergic SPECT

This imaging technique assesses the integrity of dopaminergic terminals [251].

Strengths

It has already been used as an outcome measure in disease-modifying trials in PD (e.g., BIIB054, NCT03318523), and is available in several centres for a moderate cost. SPECT studies are preferable to PET from an economic and infrastructure point of view, since they do not require an on-site cyclotron or radiochemistry facilities, and their radiotracers are produced industrially, which reduces costs and potentially allows for assessment of larger patient groups [252].

Regarding clinical meaningfulness, studies show a good correlation of DAT levels with bradykinesia and rigidity, and less with tremor [253,254].

The burden for the clinician is relatively low, and only implies training in the interpretation of the results.

It is a reliable technique, and Dopamine transporter (DAT) 123-I Ioflupane SPECT imaging (DaT-SCAN) is the only commercially approved functional imaging modality

to establish presence of presynaptic dopamine deficiency [219]. Moreover, it is a valid technique, as the degree of DAT depletion correlates with motor impairment severity, especially between DAT uptake contralaterally to the clinically more affected side [255,256].

Regarding possible “cut-offs” in this case, it is worth mentioning that in clinical practice, DAT imaging is interpreted qualitatively based on the visual interpretation, whereas quantitative analysis is routinely used in the research domain.

Dopaminergic SPECT techniques can inform about the presence of dopaminergic loss and, to a lesser extent, about its severity.

Limitations

The correlation between PD clinical features and DAT levels described above seems to be limited to the baseline scans, because longitudinal evidence suggests that there is no correlation between change in the striatal DAT uptake and the change in UPDRS motor score [253,254].

It is a burdensome test for some patients, as some medications might need to be stopped before the test [251]. Furthermore, its results are affected by medication, and various studies have described normal or upregulated binding of postsynaptic dopamine D2 receptor PET and SPECT ligands is in treatment-*naïve* PD, and reduced in medicated PD subjects [257,258].

Dopaminergic SPECT imaging may have floor effects, as a study described that the reduction in DAT binding was more marked in ipsilateral than in contralateral putamen [219].

Finally, this imaging technique is less effective at later stages: a study reported that the annualized change in DAT binding was greatest at year 1 when compared with years 2

and 4, consistent with recent pathology data suggesting that DAT terminal have largely disappeared by year 4 [219]. In line with this, dopaminergic SPECT might be of special relevance in pre-disease, early stage and *de novo* PD.

DAT imaging has poorer sensitivity in very early-stage disease: based on meta-analysis of available literature, 1 in 5 scans will be normal (in most DAT validation studies, the disease duration is approximately 6 years and when this is restricted to less than 2 years, accuracy is 80%).

Furthermore, there are ceiling effects, and the normalisation procedures (for so-called quantitative DAT) also pose challenges.

Dopaminergic PET

There are several imaging dopaminergic PET studies available: for instance, 18Fluoro-dopa PET assesses the integrity of dopaminergic terminals [259], and DAT PET studies (e.g., 11C-PE2I) focus on dopamine transporters [260].

Strengths

As it was the case with the previous technique, dopaminergic PET imaging has already been used in disease-modifying trials in PD.

18F-dopa PET levels correlate with dopamine cell densities in the substantia nigra and with striatal levels of dopamine [259], and in the case of DAT-PET, there are good correlations between DAT levels and bradykinesia and rigidity, but less with tremor [260].

DAT-PET (e.g., 11C-PE2I) correlates clinically and accordingly to PD progression: a recent study described a strong correlation between 11C-PE2I uptake and UPDRS over time, apart from tremor [260].

Dopaminergic PET techniques can inform about the presence of dopaminergic loss and its severity. Furthermore, a study revealed that nigrostriatal dopaminergic denervation detected in [11C]dihydrotetrabenazine (DTBZ) monoaminergic PET imaging can predict fatigue in mild PD, but not in moderate-to-severe PD [261].

Limitations

Its feasibility is very low, as it is a costly, not widely available technique. Similarly to dopaminergic SPECT, dopaminergic PET can be burdensome for some patients, as some medications might need to be stopped before the test [251].

Being a dopaminergic imaging technique, its effectiveness decreases with disease progression. A study showed that F-Dopa uptake decreases in different regions of the striatum at different rate during the progression of PD: the putamen uptake decreases faster in the early stages of PD, the caudate uptake stays relatively stable at least during the first years of the disease [262]. Consequently, and as with dopaminergic SPECT imaging, it might be more useful in early stages of PD.

Unfortunately, another study reported a weak correlation between F-dopa and UPDRS over time [260].

Non-dopaminergic SPECT

This section focuses on cerebral perfusion SPECT, which assesses the metabolic activity in the brain by measuring changes in the cerebral blood flow. It provides a measure of the perfusion and metabolic status of the brain tissues, which can be imaged

using lipophilic radiotracers, such as 99mTc-ECD, 99mTc-HMPAO and 123I-IMP [250]. It is mainly used in cerebrovascular diseases, epilepsy, traumatic brain injury, inflammation, assessment of brain death, and in the detection and differential diagnosis of dementia [263].

Recent and extensive reviews on the evolution of the quality of SPECT scans and the data on neurologic and psychiatric conditions [264], and on technical aspects of perfusion SPECT neuroimaging and image processing [265], have recently been published.

Strengths

As mentioned above, SPECT techniques are less costly and therefore more feasible.

There is less available literature on the value of perfusion SPECT for monitoring disease progression in PD, since this technique has been mainly used to aid the differential diagnosis of dementia [263].

Consequently, it might be of particular use in PD patients with cognitive impairment, and there has been a study correlating its findings in probable PD dementia patients with neuropsychometric testing and structural MRI, although the authors conclude that no distinct pattern is found in either test, confirming the previously described overlap of PD dementia with other neurodegenerative dementias [266].

A recent meta-analysis concluded that cerebral perfusion imaging has good prognostic value for mild cognitive impairment [267].

Limitations

To our knowledge, this technique has not been used in disease-modifying trials in PD.

Its interpretability is lower than that of dopaminergic imaging techniques, because it only informs of hypoperfusion of different cerebral regions, which can be multifactorial. In line with that, a study revealed that its ability to differentiate PD from MSA was uncertain [268].

It takes around 2 hours to complete and an intravenous injection, with the consequent burden in patients, and needs an expert assessor to interpret the imaging results. Caffeine, alcohol, and drugs affecting the cerebral blood flow interfere with the results of brain perfusion SPECT, as do comorbidities (e.g., psychiatric conditions), which need to be taken into account when analysing the images [269].

This, added to its unclear validity and high costs, makes it unlikely to be an acceptable measure to regulatory bodies.

Non-dopaminergic PET

Non-dopaminergic PET can measure different parameters: neurotransmitter systems (cholinergic, serotonergic, noradrenergic), proteins (amyloid, tau, alpha-synuclein), glucose metabolism, and neuroinflammation.

Strengths

Some non-dopaminergic PET techniques (18FDG-PET, [11C]-PK11195 PET non-dissociable binding potential (BPND)) have already been used in disease-modifying trials in PD.

The validity of these techniques for what they intend to measure changes depending on the specific technique. In the case of serotoninergic PET imaging, it is variable, and a study reported altered imaging in some PD patients, but similar results to HC in others [270]. As opposed to that, cholinergic PET has shown significant correlations between

attention, memory, and executive function domains and global cortical acetylcholinesterase hydrolysis rates [271]. A current longitudinal cohort study includes cholinergic PET as part of the phenotyping instruments for a *de novo*, treatment-*naïve* at baseline PD population [272]. The authors hypothesise that cognitive impairment will be related to cognitive impairment related to regional cortical and subcortical cholinergic denervation, and that such denervation will predict cognitive decline in PD over time.

There are different suggested cut-offs depending on the specific target: e.g., for amyloid 18F-florbetaben (FBB) PET to detect PDD, some authors identified a centiloid cut-off of 31.3 [273].

FBB PET has a high sensitivity (97.9%) and specificity (88.9%) for detecting histopathology-confirmed neuritic beta-amyloid plaques [274].

Evidence suggests that non-dopaminergic PET imaging techniques may be more sensitive to change in later disease stages [270,271]. In line with that, advanced PD and cognitive subgroups might be the best target population for these techniques, the latter especially in the case of serotoninergic, cholinergic (memory, attention, and executive functions) and amyloid PET. In fact, a cross-sectional study in PD patients with and without MCI showed an extremely low prevalence of beta-amyloid positivity in PD without dementia compared with cognitively normal elderly controls [275], and a different study showed higher prevalence of executive dysfunction and neuropsychiatric symptoms in PDD patients with amyloid deposition in PET imaging [276]. However, the role of amyloid PET in PD is controversial, and a more recent study did not show a correlation between amyloid PET and cognitive impairment in PD (see *Limitations*).

Serotoninergic PET studies have shown an inverse correlation between serotoninergic innervation and cortical beta-amyloid burden, both measured via PET, which led to

hypothesise about the potential role of serotoninergic medications in reducing beta-amyloid deposition and in doing so, the risk of cognitive impairment in PD [270]. A recent study has also shown the ability of [11C]DASB serotoninergic PET imaging to distinguish between multiple system atrophy (MSA) and PD and suggested a correlation of imaging changes with motor severity in MSA, but this was not found in PD [277]. However, another study reported serotoninergic dysregulation IN ([11C]MADAM) PET as PD progresses, in the form of disturbed signalling from raphe nuclei to subcortical and cortical regions [278].

Given its ability to discriminate DLB from PD and to identify underlying pathology, some authors have suggested the combination of amyloid PET with DAT studies as a biomarker in trials of targeted PD therapies [279].

They can inform of the severity of the disease, as several of these techniques have shown clinical stage-dependent changes in (e.g., adenosine A2A PET [280]).

Limitations

Despite its use in previous disease-modifying trials in PD, its high costs and unclear validity make it a less acceptable outcome measure altogether, except for targeted subgroups, such as for the assessment of target engagement or treatment effect in specific trial arms (e.g., azathioprine [141], istradefylline [281]).

Being relatively novel and specific techniques, they are less widely available, with the subsequent impact on feasibility. They take between 2 to 3 hours in total and an intravenous injection, which can make this technique burdensome to patients, and an expert is required to interpret the results [282]. Depending on the specific tracer used, patient medications (e.g., dopaminergic treatment [280]) and comorbidities (e.g.,

hyperglycaemia in the context of diabetes mellitus [282]) can influence the results and difficult interpretation.

Techniques such as adenosine A2A PET are influenced by symptomatic PD therapy [280], which complicates its interpretation in a disease-modifying trial. Although this influence does not seem relevant in the short term, repeatability issues have been reported with this particular technique [283].

The utility of these techniques is yet to be elucidated. In line with that, it was recently reported that, unlike nigrostriatal dopaminergic denervation, cholinergic denervation is not a predictor of fatigue in PD [261].

Amyloid PET is considered appropriate when the patient has MCI, and/or fulfils core clinical criteria for possible Alzheimer's disease, and/or has early-onset (≤ 65 years of age) progressive dementia [284], which makes it less relevant as an outcome measure in a disease-modifying PD trial unless cognitive interventions are included. In a recent study, amyloid PET was not associated with cognitive impairment in a moderately large PD sample, enriched with PD-MCI patients at risk of dementia, which suggests that other pathways are more relevant in the development of cognitive impairment in PD [273].

Magnetic Resonance Spectroscopy (MRS)

This technique has been reviewed elsewhere [285]. In short, and with similar theoretical principles to magnetic resonance imaging (MRI), proton MRS can study different metabolites, which give an indirect measure of neuronal status and the functioning of various intracellular organelles and processes (e.g., mitochondria, protein and lipid synthesis).

Strengths

MRS is a non-invasive technique, and it is consequently less burdensome for patients. Furthermore, it does not need extra equipment other than additional software and hardware to process MRI images. It also has good test-retest reliability and, compared with the above-mentioned molecular imaging techniques (PET, SPECT), it is less costly and does not require exposure to radioactive tracers [285].

It has been suggested both as a diagnostic aid, especially in the differential diagnosis of parkinsonism and in early PD, and as a marker of treatment response. Regarding the latter, N-acetylcholine (NAA) recovery has been postulated as a biomarker of response to dopaminergic treatment [285].

More recently, a phase 2 disease-modifying trial of ursodeoxycholic acid (UDCA) in PD has used midbrain ^{31}Phosphorus Magnetic Resonance Spectroscopy (^{31}P-MRS) as a marker of target engagement [286].

Limitations

The NAA variations in patients on symptomatic therapy makes the interpretation of these results in disease-modifying trials extremely challenging, and its use warrants further investigation.

Structural neuroimaging

As mentioned above, neuroimaging in PD has been recently reviewed elsewhere [250].

This section focuses on magnetic resonance imaging (MRI) imaging sequences and techniques.

The main strengths of these outcome measures are that they are widely available, the burden to patients and clinicians is, in general, low (moderate for PD patients with more difficulty to stay still for the duration of the test, and for clinicians in cases where post-imaging processing is needed, as in volumetric studies), and they have been broadly used in PD trials. Regarding disease-modifying PD trials, structural imaging has also been already included as an outcome measure (e.g., memantine (NCT03858270), intraputaminal adeno-associated virus type 2 (AAV2)-neurturin [287]).

All of the above make this structural imaging techniques likely acceptable to regulators, their main obstacle being, in some cases, the lack of experience with use in disease-modifying trials of PD.

For all MRI-based metrics, it is essential to factor in the inter-scanner variation, which can be significant, although minimised in true quantitative acquisitions, and in line with that, to perform phantom/travelling head standardisation. From an analysis perspective, identifying and aligning structures of interest will be an essential step for all imaging methods.

Moreover, the analyses intended to be performed must be considered when designing the imaging package, and isotropic sequences are essential for any morphometric analysis.

The NINDS-CDE version 2.0 has classified MRI and Spectroscopy as Supplemental – Highly Recommended.

T1 structural sequence

T1 brain MRI sequences are of most use for assessing volume or cortical thickness.

Strengths

It is widely available, and not particularly burdensome to patients or clinicians, although volumetric studies might require post-imaging processing before interpretation. Its main utility is as a vehicle to assist in the analysis of quantitative sequences rather than as an outcome measure in isolation.

Volumetric studies derived from T1 sequences might be of particular interest in PD subgroups with cognitive impairment, or to assess interventions which target this problem. A recent review highlighted common findings across longitudinal grey matter studies in PD, such as hippocampal thinning, although with some caveats (see *Limitations*).

It can inform of the presence of atrophy and its severity, as well as monitor it through repeat assessments, although its significance might be difficult to elucidate.

A study correlated the grey matter intensity and total MDS-UPDRS part III scores, finding a significant association between rigidity scores (anterior striatum) and severity of axial symptoms (left-sided anterior striatum and precentral cortex) [288].

Limitations

Generally speaking, it is a poor marker in isolation. In line with that, frontal and cingular atrophy were correlated to progression to PDD, but its predictive value increased considerably when combined with clinical and wet biomarkers [289].

The validity of this technique is not completely clear in this scenario, and there are inconsistent findings in the literature about its validity and sensitivity to change [290–292].

Diffusion imaging

MRI diffusion imaging techniques offer an insight into white matter integrity.

Strengths

It is widely available and interestingly, reduced fractional anisotropy (FA) has been described in the substantia nigra (SN) in early PD [293]. Other techniques that might be more sensitive include free water in white matter and higher order models. This publication focuses on potential imaging outcome measures for disease-modifying trials in PD, and a review of diffusion imaging techniques can be found elsewhere [294].

Regarding sensitivity to change, this might be better in later-stage PD, as a study already showed white matter loss in higher-risk patients for dementia [159].

Diffusion imaging informs of severity of white matter involvement, and could potentially differentiate PD from atypical forms [295].

Free-water imaging can detect neuroinflammation as well as neurodegeneration with more accuracy than classical diffusion tensor imaging studies, and can characterise the aetiology of microstructural changes. A recent study reported neuroinflammatory changes preceding neurodegeneration, and microstructural white matter alterations preceding grey matter changes in PD [296].

Within diffusion imaging, multiple B-shell diffusion imaging opens the doors to a broad variety of measures, that can all be derived from the same sequence. It has the added advantage of being robust to motion, and has excellent reconstruction pipelines to deal with issues, and is very quick with the modern multiband accelerated sequences.

The reliability of diffusion imaging depends on the specific technique (the classical techniques are less reliable than newer approaches), the amount of information available for analysis, and the purpose of the study (e.g., deep brain stimulation planning needs less data to achieve reliability than functional MRI) [294].

Akin to T1 and volumetric studies, diffusion imaging might be of particular use in PD patients with cognitive impairment.

Limitations

Similar to molecular neuroimaging techniques, the limited availability of some diffusion techniques and the expert staff to process and interpret them makes them less feasible than traditional sequences.

The data in PD progression, although promising, is still limited, but this should not preclude its implementation in upcoming trials, either as an exploratory or for specific PD subgroups or treatment arms.

Multiple Parametric Mapping Protocol

This is a 21-minute 1mm isotropic protocol that provides MT, R1, R2s, and proton density sequences.

Strengths

Its main utility is that it allows for mapping and aligning structures that are affected in PD (i.e., substantia nigra and brainstem nuclei) at a single subject level.

Limitations

This protocol has not been previously used directly in trials and, depending on the system, it might pose a challenge (e.g., it is currently not possible to run on Philips devices).

Neuromelanin

This MRI technique is sensitive to neuromelanin within the substantia nigra (SN).

Strengths

This technique is potentially meaningful clinically and has good validity, as shown by a study that reported reduced signal in posterior SN in early PD [297].

It is sensitive to change, as reported in various studies [297,298], shows the presence/absence of neuromelanin in SN, and is potentially useful for all PD patients.

Limitations

Although MRI is widely available, neuromelanin analyses are not yet widespread, and clinically meaningful cut-offs are not yet known.

Furthermore, most are not quantitative, nor are they designed for volumetric analysis. On the contrary, they provide a restricted window, designed for qualitative analysis. The normalisation procedure (i.e., dividing by pontine signal) assumes that there is no disease in the pons, which is incorrect and would also correlate with severity, so it is important to decide how to best use this sequence (e.g., as a way to identify LC to extract Quantitative Susceptibility Mapping (QSM)).

Iron-sensitive sequences

Sequences such as R2* (1/T2*) and quantitative susceptibility mapping (QSM) are susceptibility sequences developed to detect iron in brain tissues.

Strengths

Iron-sensitive sequences have been reported to relate to clinical measures [299,300], which makes them a potential clinically meaningful imaging technique.

They are interpretable at the level of presence (early-stage PD) and severity, and similarly to diffusion techniques, they might help differentiate PD from atypical parkinsonisms [301].

Quantitative susceptibility maps have increasing levels of evidence supporting their use, and even though they would be more exploratory, on a trial basis, they are proper measures of brain microstructure, and therefore would be more robust to measurement error (seen in clinical metrics).

A recent meta-analysis reported a pooled sensitivity of 92% and a pooled specificity of 90%, and identified longer mean disease duration (≥ 5 years), subjective analysis, a smaller size of pixel (< 0.6 mm2), larger flip angle (> 15°), smaller slice thickness (≤ 1 mm), and specific targeting of the substantia nigra pars compacta as factors improving the diagnostic performance [302].

Limitations

Although MRI is widely available, iron-sensitive analyses are not yet widespread, and clinically meaningful cut-offs are not yet known. Moreover, lack of longitudinal data makes it difficult to assess their sensitivity to change.

Wet biomarkers

Some blood and cerebrospinal fluid (CSF) parameters have been found to yield some diagnostic and/or prognostic value in PD [303], and have even been correlated with its neuropathological findings and with other neurodegenerative conditions such as Alzheimer's disease (AD) [304].

Regarding laboratory tests and biospecimens/biomarkers, the NINDS-CDE has only classified PD Genetics as Supplemental – Highly Recommended and Supplemental. Consequently, none of the below biomarkers have been included as such in the NINDS-CDE version 2.0.

Strengths

All biomarkers are, to a greater or lesser extent, feasible at the cost of the corresponding assay, and if it is unavailable in the study site, the sample can be sent out to a central laboratory.

In terms of patient and clinician implications, blood biomarkers bring about a relatively low burden in both patients and carers, as they involve a blood draw.

Wet biomarkers (e.g., CSF and serum alpha-synuclein) have already been included as outcome measures in disease-modifying PD trials [305].

Limitations

Contrary to blood biomarkers, CSF determinations require clinician training in lumbar puncture (LP), which is an invasive and uncomfortable procedure for patients, and hence their use should be especially justified.

Furthermore, to date there are no blood or CSF clinically meaningful cut-off levels of any biomarker in PD, although this is currently being investigated by numerous groups.

Although wet biomarkers constitute a promising approach, and their levels can be quantified and monitored, there is currently not enough evidence to support their use as a measure of primary target engagement, and its clinical meaningfulness is unclear [303,306–308]. For those reasons, acceptability to regulatory bodies is lower than other measures such as patient-reported outcomes.

Plasma/serum neurofilament light chain (NfL)

NfL is a neuronal cytoskeletal protein released during axonal damage, which has been studied as a potential biomarker in several neurological conditions such as AD, amyotrophic lateral sclerosis (ALS), multiple sclerosis (MS), and progressive supranuclear palsy (PSP).

High sensitivity assays are available, namely Simoa (Lower Limit of Detection (LLOD) = 0.038 pg/mL) and Meso Scale Discovery (MSD) (LLOD = 5.5 pg/mL), but additional assays are in development by clinical chemistry companies like Roche Diagnostics, Fujirebio and Siemens Healthineers.

Serum NfL measured using Simoa technology was stable with a 24-hour delay before freezing and through up to three freeze thaw cycles [309], and when processing is delayed for 7 days at room temperature [310]. Serum and plasma NfL levels correlate highly, but plasma levels have been found to be around 25% lower than serum concentrations [311]. Multi-centre validation studies of NfL are being carried out to ensure inter-site reliability to progress the use of NfL in future trials [312].

Serum NfL has been found to be raised in PD patients vs controls, and increased longitudinally in PD patients compared with controls, indicating its validity (but low specificity) as a marker to support the diagnosis of PD [313]. In another study, serum NfL was also found to be 37% higher in PD patients compared with controls with an AUC of 0.64 [314]. In a further study, a serum NfL cut-off of 13.75 pg/ml had a sensitivity of 76% and specificity of 85% for distinguishing between PD and healthy controls, and with a cut-off of 13.65 pg/ml had a 76.7% sensitivity and a 84.1% specificity to distinguish between PD and essential tremor (ET) [315].

Despite the promising findings, it is important to consider that serum NfL is not specific for PD pathology and is therefore not a diagnostic marker in PD, as there is a substantial overlap between patients with PD and healthy controls.

Regarding correlations between NfL and clinical parameters, higher serum NfL levels were associated with older age in healthy controls, patients with PD and patients with essential tremor (ET), and a positive correlation between serum NfL levels and motor severity (UPDRS part II, HY) was seen [315]. A positive correlation of serum NfL and motor score was also found in data from the Parkinson's Progression Markers Initiative (PPMI) [313], and another study reported that higher serum NfL was associated with a lower Mini-Mental State Examination (MMSE) score [314].

In terms of particular PD subgroups, serum NfL measured using the Simoa platform stratified patients into subtypes of later PD according to their likelihood to reach clinically relevant progression milestones during a long-term observational study (walking-aid usage (hazard ratio (HR): 3.5; 95% confidence interval (CI) 1.4–8.5), nursing home living (5.1; 2.1–12.5), motor end-stage (6.2; 2.1–17.8), and death (4.1; 1.7–9.7) [316]. Moreover, in a longitudinal study, PD patients classified at baseline as postural instability gait disorder (PIGD) subtype had higher plasma NfL levels than PD patients classified as tremor-dominant (TD) subtype [317]. Serum NfL levels have also been found to be related to worse cognitive performance and a cortical macro- and microstructural compromise ($p < 0.005$ corrected) in data from PPMI [318].

Although blood NfL may be less sensitive to change than CSF NfL, blood NfL is more practical as a screening test, due to the less invasive nature of the blood draw compared with a lumbar puncture [319]. Future larger longitudinal follow-up studies that incorporate other biomarkers are needed to validate whether blood NfL may be used to predict PD progression [315].

Plasma tau (tau)

Different phosphorylated forms of the tau protein (p-tau) have been studied in neurodegenerative disorders such as AD [320] and dementia with Lewy bodies [321], and both p-tau and total tau (T-tau) levels have been investigated as a biomarker in PD.

Plasma tau can be measured by enzyme-linked immunosorbent assay (ELISA) or alternatively, by high sensitivity assays, such as Simoa for total tau (LLOD = 0.019 pg/mL, p-tau181 (LLOD = 0.028 pg/mL) or p-tau231 (LLOD = 0.621 pg/mL); and MSD S-Plex assays for total tau (LLOD =0.012 pg/mL), p-tau181 (LLOD = 0.077 pg/mL), and p-tau231 (LLOD = 3.1 pg/mL). Other techniques that can be used to determine plasma tau levels are immunomagnetic reduction (IMR) and enhanced immunoassay using multi-arrayed fiberoptics (EIMAF), but these technologies are less validated from an analytical standpoint.

To test reliability of total-tau, the same samples were tested in a run, and the coefficients of variation (CVs) for Simoa, IMR, a-EIMAF (EIMAF combined with rolling circle amplification), MSD, and ELISA were 9%, 5.04%, 5.51%, 7.9%, and 2.9%, respectively. Simultaneously, the influence of different lab/instrument for Simoa, IMR, and a-EIMAF was evaluated, and the CVs were 7.3%, 4.76%, and 5.16%, respectively [322], providing evidence for the reliability of the tau assay.

Regarding diagnostic value, in a study of several plasma biomarkers, total tau and p-tau were raised in PD patients compared with healthy controls (HC) (total tau levels (pg/ml): HC: 12.12 +/- 0.96, PD: 20.32 +/- 2.73; p-tau levels (pg/ml) HC: 1.52 +/- 0.18, PD: 3.98 +/- 1.38) [323]. In another study of plasma biomarkers to detect cognitive impairment, plasma total tau levels were also significantly raised in PD patients

compared with HC (HC: 14.67(11.02, 22.48), PD: 31.87 (26.06, 3733) [324]. Nevertheless, there is currently no blood-based measurement of tau with clinically meaningful diagnostic performance.

In terms of prognostic value, a recent study reported that in PD, increased plasma p-tau181 is related to worse motor symptoms, which leads to hypothesise that tau pathology may influence motor progression in PD. In that paper, plasma alpha-synuclein (α-syn) and p-tau181 were positively correlated and associated with motor and cognitive dysfunction in *de novo* PD, being therefore suggested as a biomarker panel for the detection of *de novo* PD [325]. T-tau was also independently associated with PD patients with cognitive impairment (odds ratio (OR) = 1.069, 95% CI = 1.026–1.115), with a T-tau cut-off value for predicting cognitive deficits in PD patients of 30.6 pg/ml of T-tau (AUC = 0.726) [324]. However, the precise meaning of this concentration is uncertain, as the assay is research-grade without standardization against a certified reference material. Given the correlation of p-tau181 with motor severity and the long-studied relationship between tau pathology and cognitive decline, this biomarker could be particularly helpful in advanced PD and PD patients with cognitive complaints.

Combinations of biomarkers have also been evaluated, and in regards to this, plasma beta-amyloid 42 (Aβ42) combined with plasma p-tau181 was found particularly useful for discriminating patients with frontotemporal dementia (FTD) from patients with PD and atypical parkinsonian syndromes (AUC = 0.932) [323]. Also, the combination of plasma p-tau181 with plasma α-syn, age and sex, showed good performance in discriminating *de novo* PD patients from HC (AUC = 0.806) [325].

The above evidence supports the future use of plasma p-tau181 as a diagnostic tool, especially in combination with other biomarkers.

Plasma alpha-synuclein (α-syn)

Akin to tau, total, phosphorylated and oligomeric levels of α-syn are wet biomarkers commonly studied in PD.

Total α-syn can be measured by immunoassay, and some examples are: LEGEND MAX™ Human a-Synuclein ELISA Kit (LLOD = 6.1 pg/mL), Simoa (LLOD = 0.955 pg/mL), and MSD (LLOD = 0.9 pg/mL). Exosomal alpha synuclein can also be measured by MSD. Comparison of three different platform immunoassays for the measurement of plasma total α-syn (ELISA, Simoa, and MSD) showed significant correlations between all three platforms: ELISA and MSD ($r=0.6718$, $p<0.000$), ELISA and Simoa ($r=0.6255$, $p<0.0001$), and MSD and Simoa ($r=0.7822$, $p<0.0001$) [326]. A major discovery was the fact that > 99% of the α-syn resides in the red blood cells (RBC) and less than 1% remains free in the plasma, and this implies that plasma α-syn levels can increase artificially with RBC contamination, which results in low precision for blood α-syn measurements [327].

There is conflicting evidence regarding the serum or plasma levels of total α-syn in PD and HC: there are studies reporting that α-syn is higher, lower and not significantly different in PD versus HC [328]. In parallel, the case is the same with RBC total α-syn [303]. As a consequence, studies on measurement of plasma total α-syn have not progressed as much as other wet biomarkers of PD.

Contrary to the results from total α-syn, oligomeric serum α-syn is increased in PD and can help identify PD from AD samples [329]. Also, another form of α-syn , RBC-derived Serine 129-phosphorylated α-syn (pS-α-syn), separated PD patients well from

HC, with a sensitivity of 93.39% (95% CI: 90.17-95.81%), a specificity of 93.11% (95% CI: 89.85-95.58%), and an AUC of 0.96 [330].

There have also been promising results for alpha synuclein from neuronal exosomes as a diagnostic marker: total α-syn levels in plasma neuronal exosomes were significantly higher in PD patients than controls [331]. More recently, it has been shown that pathological α-syn derived from blood plasma neuronal extracellular vesicles was significantly increased in PD patients versus controls and using an α-syn seeding assay, misfolding of the α-syn could be shown, demonstrating the potential for this to evolve into a blood-based biomarker of PD [332].

Regarding clinical significance of blood α-syn, pS-α-syn has been reported to correlate with motor stage [330]. Interestingly, the plasma levels of pS-α-syn remain high and do not change during the course of the disease, whereas the level of total α-syn tends to increase over time for up to 20 years after the initial symptoms of PD. This can be best explained by a steady increase in the concentration of plasma non-phosphorylated α-syn levels as the disease progresses [333]. Some evidence shows that plasma α-syn might be particularly useful in advance-stage PD, and cognitive subgroups: in a recent study, higher plasma α-syn levels were significantly associated with worse UPDRS Part III motor scores, higher modified HY stages, and increased risk of PD with mild cognitive impairment (PD-MCI) ($P < 0.05$) [325]. Moreover, neuronal exosomal α-syn concentrations were significantly correlated with MDS-UPDRS part III/(I + II + III) scores, NMSQ scores, and Sniffin' Sticks 16-item test scores in a cohort of PD patients. Additionally, after a mean follow-up of 22 months in patients with Intermediate PD, a Cox regression analysis adjusted for age and gender showed that longitudinally increased neuronal exosomal α-syn levels rather than baseline levels were associated with higher risk for motor symptom progression in PD ($P = 0.039$) [331]. Another form

of α-syn, RBC-derived pS-α-syn, has been reported as potentially predictive of subtypes and stages of PD [330]. All of the aforementioned studies provide supporting evidence that some forms of α-syn might be robust predictors of motor and cognitive outcome in PD.

The potential utility of α-syn for PD subgrouping is variable depending on which form is studied. On the one hand, plasma α-syn was not found to differ between the postural instability gait disturbance (PIGD) or tremor dominant (TD) subtypes of PD, or relate to the severity of PD [334]. On the other hand, the association of exosomal total plasma α-syn with akinetic-rigidity symptom severity in PD patients led the authors describing it to suggest its potential use to subtype PD patients [335].

Although plasma α-syn is not currently an established diagnostic tool for PD, and despite its conflicting evidence on the significance of its levels, some forms of α-syn hold promise to become future diagnostic markers for PD (neuronal exosomal α-syn, pS-α-syn).

CSF neurofilament light chain (NfL)

In the case of CSF NfL (cNfL), it has been correlated with several parameters in PD patients, such as subtype, age, motor and cognitive scores [336,337]. Additionally, some sensitivity and specificity values of cNfL have been described, to differentiate multiple system atrophy (MSA) from PD, DLB, and HC [338].

Regarding the predictive value of cNfL, there is class II evidence that in patients with PD, cNfL concentrations are associated with more severe disease and shorter survival. After adjustment for age and sex, higher cNfL correlated with striatal dopamine transporter uptake deficits and lower fractional anisotropy in diffusion tensor imaging of

several axonal tracts [339]. In another study, cNfL levels correlated with motor and cognitive impairment as well as with age, but the conversion to cognitive impairment could not be predicted by the baseline cNfL level [338].

As with serum NfL, there is no clinically meaningful cut-off for cNfL, and even though cNfL levels have been shown to be raised in atypical PD (APD) patients, the optimal cut-off values for diagnosis varies considerably between studies. CSF NfL concentrations above the median of 903 ng/L have been reported to confer PD patients an overall 5.8 times increased hazard of death during follow-up [339].

Given the above findings, cNfL might be of special interest in early and *de novo* PD, since the increase of cNfL with age in the general population makes it difficult to differentiate PD from HC in older subjects, and requires carefully selected age-related cut-offs for NfL [337]. CSF NfL may also be helpful in cognitive subgroups, as high cNfL, low Aβ42, and high heart fatty acid-binding protein are related to future PD with dementia (PDD), although cNfL as such cannot predict the conversion to PDD [340].

Akin to other wet biomarkers, cNfL is not a diagnostic tool for PD but might be a useful parameter especially in combination with other biomarkers [338].

CSF tau

Phosphorylated tau protein (p-tau231, p-tau181), and total tau can be quantified in CSF as a biomarker for PD. The feasibility of this biomarker is same as for plasma tau, and in the case of CSF, there is also the possibility of using Elecsys® total tau and p-tau181 assays.

A study assessing the Elecsys® CSF total tau and p-tau 181 assays reported that both of them have high sensitivity (limit of quantitation (LoQ): 63 pg/mL (tTau); 4 pg/mL

(pTau)) and linearity over the measuring range (80–1300 pg/mL; 8–120 pg/mL), with consistent lot-to-lot and platform comparability demonstrated good consistency (Pearson's r: 0.998; 1.000). There was also a high precision with repetitive and between laboratory measures for both assays [341].

Unlike the good performance of its assays, studies on CSF total tau and phosphorylated tau have not succeeded in showing a distinctive “PD profile”, with findings of both lower and not significantly different CSF tau values in PD compared with HC and other parkinsonian disorders [303].

Regarding sensitivity to change, CSF tau has been reported to be either normal or low in early disease stages, but increased in a proportion of late-stage PDD cases [304].

There is some evidence on the predictive abilities of CSF tau in PD. In a study on CSF tau and beta amyloid (Aß42) in PD, once levodopa treatment was initiated, higher CSF p-tau and CSF p-tau/Aß42 predicted subsequent decline on cognitive tasks involving both memory and executive functions [342]. Thus, and similarly as with serum tau, CSF tau might be particularly useful in advanced PD and in cognitive subgroups, and could help with stratification as it has shown predictive value for the development of cognitive decline in PD.

CSF alpha-synuclein (α-syn)

As is the case with plasma, CSF levels of α-syn (total, oligomerised, phosphorylated) are one of the most commonly studied biomarkers in PD. The feasibility of CSF α-syn is the same as for plasma α-syn, with assays already described in that section.

The reliability of measurement of CSF total α-syn was assessed using four different immunoassays: Elecsys® Total α-Synuclein Prototype Assay, MSD® U-PLEX Human

α-Synuclein Kit, BioLegend® α-Synuclein Immunoassay, and the α-Synuclein Prototype Immunoassay from ADx®. Each of the four methods showed high analytical precision, excellent correlation between laboratories (R^2 = 0.83–0.99), and good correlation with each other (R^2 = 0.64–0.93), although the slopes of the regression lines were different between the four immunoassays [343].

The validity of α-syn to distinguish between PD and HC is variable and, in the case of total α-syn has a low diagnostic accuracy, with a pooled sensitivity of 78–88% and a specificity of 40–57% for total α-syn in the CSF which is still unsatisfactory to sufficiently discriminate PD from controls [303].

There is no clinically meaningful cut-off for CSF α-syn, but some cut-offs to potentially differentiate PD from other conditions have already been described, e.g., a cut-off of ≤ 865 pg/mL total α-syn level is suggested to distinguish synucleinopathies (PD/MSA) from tauopathies (PSP/CBD) [344]. Given that all available α-syn assays are research-grade and not standardized to a certified reference material, cut-off values need to be interpreted with caution.

Regarding sensitivity to change, CSF levels of oligomeric α-syn showed a longitudinal increase, and the change in oligomeric α-syn/total α-syn was associated with motor deterioration, particularly in PIGD [303,345]. Based on those results, CSF α-syn, and especially the ratio of oligomeric α-syn to total α-syn in CSF, might be of especial relevance in the PIGD subgroup of PD patients.

As with serum α-syn , the interpretability of CSF α-syn is variable, depending on the α-syn species (total, oligomerized, phosphorylated).

CSF alpha-synuclein (α-syn) aggregation

Alpha synuclein aggregation is progressively gaining relevance as a promising biomarker in PD.

It can be determined by assays using protein misfolding cyclic amplification (PMCA) or real-time quaking-induced conversion (RT-QuiC), the latter is described using commercial products for all assay components [346].

CSF α-syn aggregation is clinically meaningful for PD diagnosis and there is increasing evidence showing that the use of CSF α-syn aggregation assays could be beneficial in trials [347]. Future efforts should focus on further optimization of the assay using multiple fluorophores, improving the mechanistic insight to the determinants of α-syn aggregation that relate to assay quantification and interlaboratory comparison of the assays. These measures are a pre-requisite for the widespread clinical implementation of the α-syn RT-QuIC in the future [348].

In terms of reliability, a cross-validating study comparing two types of CSF α-syn aggregation assays (PMCA and RT-QuiC) run in different laboratories showed 92% concordance [347].

As for sensitivity and specificity of CSF α-syn aggregation as a biomarker, in the above study, concordant RT-QuiC and PMCA results gave an AUC for the diagnosis of PD versus controls of 0.95[347]. In another study it was reported that 66 of 74 Parkinson's disease patients and 2 of 55 controls tested positive for the RT-QuiC assay, corresponding to a sensitivity of 89% (95% confidence interval [CI] 80, 96%) and specificity of 96% (95% CI 88, 100%) [348]. Another study found that CSF samples gave positive signals in 105/108 (97%) PD cases versus 11/85 (13%) HC, with an AUC of 95% [349]. Finally, it was shown that the CSF α-synuclein RT-QuIC assay was positive in 47 (90%) patients with isolated RBD (IRBD) and in four (10%) controls,

resulting in a sensitivity of 90·4% and a specificity of 90·0% for detecting iRBD, a prodrome of PD [350].

Leading on from this work, when comparing RT-QuiC results to PMCA, a cut-off of 1,000 max relative fluorescence units (RFU) for PMCA was suggested for "synuclein positivity" [347].

In regards to prognostic value, several studies have failed in finding a correlation between RT-QuiC quantitative parameters and PD clinical scores [347–349], but a study showed that the T50 values of the PMCA assay correlate with HY stage in 76 PD patients [351].

With regards to PD subtypes, in one study, ten PD patients with extremely high RT-QuiC V max values of > 100,000 RFU were significantly older ($p=0.03$) and had higher scores in the PIGD part of the MDS-UPDRS ($p=0.05$). This study also showed that for three of the four longitudinally followed idiopathic REM sleep behaviour disorder (RBD) cohorts, the RT-QuiC assay had around 90% sensitivity [348].

Much work is ongoing in the field of alpha synuclein aggregation assays in both CSF and other biofluids, which may form part of the clinical diagnosis of PD in the future and could have many uses in the diagnosis and prognosis of PD as well as the stratification and monitoring of PD patients for clinical trials.

CSF beta-amyloid (Aβ)

Beta-amyloid in the CSF has also been postulated as a biomarker in PD, in several different forms, namely Aβ40, Aβ38, and Aβ42.

Beta-amyloid can be determined with diverse assays, such as the MSDV-PLEX Aβ Peptide Panel 1 (LLOD Aβ40: 9.97 pg/mL, Aβ38: 16.7 pg/mL, Aβ42: 0.368 pg/mL),

the Simoa NEUROLOGY 3-PLEX A (LLOD tau: 0.0165 pg/mL, Aβ42: 0.147 pg/mL, Aβ40: 0.243 pg/mL), the Roche Elecsys and Lumipulse Aβ42 automated analyser, and also by ELISA/immunoassay [352,353].

In an effort to reduce inter-laboratory and inter-assay variability, certified reference materials have been created for the CSF measurement of Aβ42 [354]. Recent work has also shown that for measurement of Aβ40 and Aβ42, CSF can be stored for up to 72h at room temperature, 1 week at 4ºC, or at least 2 weeks at either -20 or -80ºC before Aβ measurement. This study also reported that centrifugation after LP or mixing before analysis is not required, and that after discarding the first 2 mL of CSF, any portion of up to 20 mL of CSF is suitable for Aβ analysis [355]. Studies now also show that CSF Aβ42/Aβ40 ratio is a better Aβ pathology test than CSF Aβ42 alone, as the ratio compensates for inter-individual production and release of Aβ into the CSF (high vs. low Aβ producers) [356].

In a recent review of Aβ and PD [357], it was seen that most longitudinal studies and cross-sectional studies suggest that reduced levels of CSF Aβ42 are correlated with PDD and its baseline value is predictive of future cognitive decline. Furthermore, there were decreased Aβ-42 levels among PD patients with gait disturbances, as well as a decreasing trend of CSF Aβ42 level in the following manner: HC > non-demented PD (PDND) > PDD. Consistently, most studies showed the significant association between cognitive impairment and reduced level of CSF Aβ42, and one group reported low level of CSF Aβ 42, 40, and 38 in a subset of newly diagnosed 109 PD patients [358]. In the same cohort, sequential regression analyses showed significant association between those CSF markers and memory cognitive domain. CSF Aβ had also been shown to be related with phonetic fluency in PDND [357]. Moreover, CSF Aβ has been suggested as a potential predictor of motor progression in PD [359].

In summary, the existing evidence suggests that CSF Aβ could be particularly helpful in cognitive and PIGD subgroups of PD patients and that and there are reliable, robust assays available for the measurement of CSF Aβ.

Salivary markers

There is a growing body of evidence supporting salivary biomarkers, and from a practical point of view, they constitute an attractive OM given their feasibility in remote trials. A recent review explores the role of salivary biomarkers in the diagnosis of PD [360], but one of the most widely studied so far is salivary α-syn. A recent systematic review and meta-analysis concluded that salivary α-syn measures (total, oligomeric, ratio oligomeric/total) are a simple, cost-effective and reliable biomarker to monitor PD progression [361], which is particularly relevant in disease-modifying trials. In line with that, a recent publication reported that salivary α-syn obtained by RT-QuIC correlates with disease severity in *de novo* PD [362], which is also relevant when interpreting the results of disease-modifying trials in different patient populations.

However, more data is needed, and salivary biomarkers should be considered Exploratory for the time being.

References

[1] Martínez-Martín P, Rojo-Abuin JM, Rodríguez-Violante M, Serrano-Dueñas M, Garretto N, Martínez-Castrillo JC, Arillo VC, Fernández W, Chaná-Cuevas P, Arakaki T, Alvarez M, Ibañez IP, Rodríguez-Blázquez C, Chaudhuri KR, Merello M (2016) Analysis of four scales for global severity evaluation in Parkinson’s disease. *npj Park Dis* **2**,.

[2] *Guy W (ed). ECDEU Assessment Manual for Psychopharmacology. Rockville, MD: US Department of Health, Education, and Welfare Public Health Service Alcohol, Drug Abuse, and Mental Health Administration, 1976.*

[3] Tomlinson CL, Stowe R, Patel S, Rick C, Gray R, Clarke CE (2010) Systematic review of levodopa dose equivalency reporting in Parkinson’s disease. *Mov Disord* **25**, 2649–2653.

[4] Vijiaratnam N, Girges C, Auld G, Chau M, Maclagan K, King A, Skene S, Chowdhury K, Hibbert S, Morris H, Limousin P, Athauda D, Carroll CB, Hu MT, Silverdale M, Duncan GW, Chaudhuri R, Lo C, Del Din S, Yarnall AJ, Rochester L, Gibson R, Dickson J, Hunter R, Libri V, Foltynie T (2021) Exenatide once weekly over 2 years as a potential disease-modifying treatment for Parkinson’s disease: Protocol for a multicentre, randomised, double blind, parallel group, placebo controlled, phase 3 trial: The Exenatide-PD3’ study. *BMJ Open* **11**,.

[5] Goetz CG, Tilley BC, Shaftman SR, Stebbins GT, Fahn S, Martinez-Martin P, Poewe W, Sampaio C, Stern MB, Dodel R, Dubois B, Holloway R, Jankovic J, Kulisevsky J, Lang AE, Lees A, Leurgans S, LeWitt PA, Nyenhuis D, Olanow CW, Rascol O, Schrag A, Teresi JA, van Hilten JJ, LaPelle N, Agarwal P, Athar S, Bordelan Y, Bronte-Stewart HM, Camicioli R, Chou K, Cole W, Dalvi A,

Delgado H, Diamond A, Dick JP, Duda J, Elble RJ, Evans C, Evidente VG, Fernandez HH, Fox S, Friedman JH, Fross RD, Gallagher D, Goetz CG, Hall D, Hermanowicz N, Hinson V, Horn S, Hurtig H, Kang UJ, Kleiner-Fisman G, Klepitskaya O, Kompoliti K, Lai EC, Leehey ML, Leroi I, Lyons KE, McClain T, Metzer SW, Miyasaki J, Morgan JC, Nance M, Nemeth J, Pahwa R, Parashos SA, Schneider JSJS, Schrag A, Sethi K, Shulman LM, Siderowf A, Silverdale M, Simuni T, Stacy M, Stern MB, Stewart RM, Sullivan K, Swope DM, Wadia PM, Walker RW, Walker R, Weiner WJ, Wiener J, Wilkinson J, Wojcieszek JM, Wolfrath S, Wooten F, Wu A, Zesiewicz TA, Zweig RM (2008) Movement Disorder Society-Sponsored Revision of the Unified Parkinson's Disease Rating Scale (MDS-UPDRS): Scale presentation and clinimetric testing results. *Mov Disord* **23**, 2129–2170.

[6] Martinez-Martin P, Rodriguez-Blazquez C, Alvarez-Sanchez M, Arakaki T, Bergareche-Yarza A, Chade A, Garretto N, Gershanik O, Kurtis MM, Martinez-Castrillo JC, Mendoza-Rodriguez A, Moore HP, Rodriguez-Violante M, Singer C, Tilley BC, Huang J, Stebbins GT, Goetz CG (2013) Expanded and independent validation of the Movement Disorder Society-Unified Parkinson's Disease Rating Scale (MDS-UPDRS). *J Neurol* **260**, 228–236.

[7] Evers LJW, Krijthe JH, Meinders MJ, Bloem BR, Heskes TM (2019) Measuring Parkinson's disease over time: The real-world within-subject reliability of the MDS-UPDRS. *Mov Disord* **34**, 1480–1487.

[8] Richards M, Marder K, Cote L, Mayeux R (1994) Interrater reliability of the unified Parkinson's disease rating scale motor examination. *Mov Disord* **9**, 89–91.

[9] Palmer JL, Coats MA, Roe CM, Hanko SM, Xiong C, Morris JC (2010) Unified Parkinson's Disease Rating Scale-Motor Exam: Inter-rater reliability of advanced

practice nurse and neurologist assessments. *J Adv Nurs* **66**, 1382–1387.

[10] Post B, Merkus MP, de Bie RMA, de Haan RJ, Speelman JD (2005) Unified Parkinson's Disease Rating Scale motor examination: Are ratings of nurses, residents in neurology, and movement disorders specialists interchangeable? *Mov Disord* **20**, 1577–1584.

[11] de Deus Fonticoba T, Santos García D, Macías Arribí M (2019) Inter-rater variability in motor function assessment in Parkinson's disease between experts in movement disorders and nurses specialising in PD management. *Neurologia* **34**, 520–526.

[12] Goetz CG, Stebbins GT (2004) Assuring interrater reliability for the UPDRS motor section: Utility of the UPDRS teaching tape. *Mov Disord* **19**, 1453–1456.

[13] Martínez-Martín P, Rodríguez-Blázquez C, Alvarez M, Arakaki T, Arillo VC, Chaná P, Fernández W, Garretto N, Martínez-Castrillo JC, Rodríguez-Violante M, Serrano-Dueñas M, Ballesteros D, Rojo-Abuin JM, Chaudhuri KR, Merello M (2015) Parkinson's disease severity levels and MDS-Unified Parkinson's Disease Rating Scale. *Park Relat Disord* **21**, 50–54.

[14] Goetz CG, Choi D, Guo Y, Stebbins GT, Mestre TA, Luo S (2022) It Is as It Was: MDS-UPDRS Part III Scores Cannot Be Combined with Other Parts to Give a Valid Sum. *Mov Disord*.

[15] Horváth K, Aschermann Z, Kovács M, Makkos A, Harmat M, Janszky J, Komoly S, Karádi K, Kovács N (2017) Minimal clinically important differences for the experiences of daily living parts of movement disorder society–sponsored unified Parkinson's disease rating scale. *Mov Disord* **32**, 789–793.

[16] Abdolahi A, Scoglio N, Killoran A, Dorsey ER, Biglan KM (2013) Potential

reliability and validity of a modified version of the Unified Parkinson's Disease Rating Scale that could be administered remotely. *Park Relat Disord* **19**, 218–221.

[17] Stillerova T, Liddle J, Gustafsson L, Lamont R, Silburn P (2016) Remotely assessing symptoms of Parkinson's disease using videoconferencing: A feasibility study. *Neurol Res Int* **2016**,.

[18] Morinan G, Hauser RA, Schrag A, Tang J, O'Keeffe J, Mds-Nms Scale Development Study Group (2022) Abbreviated MDS-UPDRS for Remote Monitoring in PD Identified Using Exhaustive Computational Search. *Parkinsons Dis* **2022**,.

[19] Stebbins GT, Goetz CG (1998) Factor structure of the Unified Parkinson's Disease Rating Scale: Motor Examination section. *Mov Disord* **13**, 633–636.

[20] Stebbins GT, Goetz CG, Lang AE, Cubo E (1999) Factor analysis of the motor section of the unified Parkinson's disease rating scale during the off-state. *Mov Disord* **14**, 585–589.

[21] Evans JR, Mason SL, Williams-Gray CH, Foltynie T, Trotter M, Barker RA (2011) The factor structure of the UPDRS as an index of disease progression in Parkinson's disease. *J Parkinsons Dis* **1**, 75–82.

[22] Vassar SD, Bordelon YM, Hays RD, Diaz N, Rausch R, Mao C, Vickrey BG (2012) Confirmatory factor analysis of the motor unified Parkinson's disease rating scale. *Parkinsons Dis* **2012**,.

[23] Levy G, Louis ED, Cote L, Perez M, Mejia-Santana H, Andrews H, Harris J, Waters C, Ford B, Frucht S, Fahn S, Marder K (2005) Contribution of aging to the severity of different motor signs in Parkinson disease. *Arch Neurol* **62**, 467–472.

[24] Evans JR, Mason SL, Williams-Gray CH, Foltynie T, Brayne C, Robbins TW,

Barker RA (2011) The natural history of treated Parkinson's disease in an incident, community based cohort. *J Neurol Neurosurg Psychiatry* **82**, 1112–1118.

[25] Velseboer DC, De Bie RMA, Wieske L, Evans JR, Mason SL, Foltynie T, Schmand B, De Haan RJ, Post B, Barker RA, Williams-Gray CH (2016) Development and external validation of a prognostic model in newly diagnosed Parkinson disease. *Neurology* **86**, 986–993.

[26] Hoehn MM, Yahr MD (1967) Parkinsonism: Onset, progression, and mortality. *Neurology* **17**, 427–442.

[27] Goetz CG, Poewe W, Rascol O, Sampaio C, Stebbins GT, Counsell C, Giladi N, Holloway RG, Moore CG, Wenning GK, Yahr MD, Seidl L (2004) Movement Disorder Society Task Force report on the Hoehn and Yahr staging scale: Status and recommendations. *Mov Disord* **19**, 1020–1028.

[28] Goetz CG, Nutt JG, Stebbins GT (2008) The unified dyskinesia rating scale: Presentation and clinimetric profile. *Mov Disord* **23**, 2398–2403.

[29] Colosimo C, Martínez-Martín P, Fabbrini G, Hauser RA, Merello M, Miyasaki J, Poewe W, Sampaio C, Rascol O, Stebbins GT, Schrag A, Goetz CG (2010) Task force report on scales to assess dyskinesia in Parkinson's disease: Critique and recommendations. *Mov Disord* **25**, 1131–1142.

[30] Makkos A, Kovács M, Pintér D, Janszky J, Kovács N (2019) Minimal clinically important difference for the historic parts of the Unified Dyskinesia Rating Scale. *Park Relat Disord* **58**, 79–82.

[31] Goetz CG, Stebbins GT, Chung KA, Hauser RA, Miyasaki JM, Nicholas AP, Poewe W, Seppi K, Rascol O, Stacy MA, Nutt JG, Tanner CM, Urkowitz A, Jaglin JA, Ge S (2013) Which dyskinesia scale best detects treatment response? *Mov*

Disord **28**, 341–346.

[32] Port RJ, Rumsby M, Brown G, Harrison IF, Amjad A, Bale CJ (2021) People with Parkinson's Disease: What Symptoms Do They Most Want to Improve and How Does This Change with Disease Duration? *J Parkinsons Dis* **11**, 715–724.

[33] Bloem BR, Marinus J, Almeida Q, Dibble L, Nieuwboer A, Post B, Ruzicka E, Goetz C, Stebbins G, Martinez-Martin P, Schrag A (2016) Measurement instruments to assess posture, gait, and balance in Parkinson's disease: Critique and recommendations. *Mov Disord* **31**, 1342–1355.

[34] Bouça-Machado R, Duarte GS, Patriarca M, Castro Caldas A, Alarcão J, Fernandes RM, Mestre TA, Matias R, Ferreira JJ (2020) Measurement Instruments to Assess Functional Mobility in Parkinson's Disease: A Systematic Review. *Mov Disord Clin Pract* **7**, 129–139.

[35] Winser SJ, Kannan P, Bello UM, Whitney SL (2019) Measures of balance and falls risk prediction in people with Parkinson's disease: a systematic review of psychometric properties. *Clin Rehabil* **33**, 1949–1962.

[36] ProFaNE Prevention of Falls Network Earth – Definition of a Fall, Last updated 2012, Accessed on 2012.

[37] Franchignoni F, Horak F, Godi M, Nardone A, Giordano A (2010) Using psychometric techniques to improve the balance evaluation systems test: The mini-bestest. *J Rehabil Med* **42**, 323–331.

[38] StatPearls [Internet], Berg Balance Testing, Last updated 2022, Accessed on 2022.

[39] Tinetti ME, Richman D, Powell L (1990) Falls efficacy as a measure of fear of falling. *Journals Gerontol* **45**, 239–243.

[40] Powell LE, Myers AM (1995) The Activities-specific Balance Confidence (ABC) Scale. *Journals Gerontol* **50A**, M28-34.

[41] Evatt ML, Chaudhuri KR, Chou KL, Cubo E, Hinson V, Kompoliti K, Yang C, Poewe W, Rascol O, Sampaio C, Stebbins GT, Goetz CG (2009) Dysautonomia rating scales in Parkinson's disease: Sialorrhea, dysphagia, and constipation - Critique and recommendations by movement disorders task force on rating scales for Parkinson's disease. *Mov Disord* **24**, 635–646.

[42] Patel DA, Sharda R, Hovis KL, Nichols EE, Sathe N, Penson DF, Feurer ID, McPheeters ML, Vaezi MF, Francis DO (2017) Patient-reported outcome measures in dysphagia: A systematic review of instrument development and validation. *Dis Esophagus* **30**, 1–23.

[43] Kalf JG, Borm GF, De Swart BJ, Bloem BR, Zwarts MJ, Munneke M (2011) Reproducibility and validity of patient-rated assessment of speech, swallowing, and saliva control in parkinson's disease. *Arch Phys Med Rehabil* **92**, 1152–1158.

[44] McHorney CA, Bricker DE, Kramer AE, Rosenbek JC, Robbins JA, Chignell KA, Logemann JA, Clarke C (2000) The SWAL-QOL outcomes tool for oropharyngeal dysphagia in adults: I. Conceptual foundation and item development. *Dysphagia* **15**, 115–121.

[45] McHorney CA, Earl Bricker D, Robbins J, Kramer AE, Rosenbek JC, Chignell KA (2000) The SWAL-QOL outcomes tool for oropharyngeal dysphagia in adults: II. Item reduction and preliminary scaling. *Dysphagia* **15**, 122–133.

[46] McHorney CA, Robbins JA, Lomax K, Rosenbek JC, Chignell K, Kramer AE, Bricker DE (2002) The SWAL-QOL and SWAL-CARE outcomes tool for oropharyngeal dysphagia in adults: III. Documentation of reliability and validity.

Dysphagia **17**, 97–114.

[47] McHorney CA, Martin-Harris B, Robbins JA, Rosenbek J (2006) Clinical validity of the SWAL-QOL and SWAL-CARE outcome tools with respect to bolus flow measures. *Dysphagia* **21**, 141–148.

[48] Lam K, Lam FKY, Kwok KL, Yiu KC, Kan EYL, Woo J, Fat KW, Ko A (2007) Simple clinical tests may predict severe oropharyngeal dysphagia in Parkinson's disease. *Mov Disord* **22**, 640–644.

[49] Leow LP, Huckabee ML, Anderson T, Beckert L (2010) The impact of dysphagia on quality of life in ageing and parkinson's disease as measured by the Swallowing Quality of Life (SWAL-QOL) questionnaire. *Dysphagia* **25**, 216–220.

[50] Carneiro D, das Graças Wanderley de Sales Coriolano M, Belo LR, de Marcos Rabelo AR, Asano AG, Lins OG (2014) Quality of life related to swallowing in Parkinson's disease. *Dysphagia* **29**, 578–582.

[51] Cardoso AR, Guimarães I, Santos H, Carvalho J, Abreu D, Gonçalves N, Ferreira JJ (2021) Cross-cultural adaptation and validation of the Swallowing Disturbance Questionnaire and the Sialorrhea Clinical Scale in Portuguese patients with Parkinson's disease. *Logop Phoniatr Vocology* **46**, 163–170.

[52] Rajaei A, Azargoon SA, Nilforoush MH, Barzegar Bafrooei E, Ashtari F, Chitsaz A (2014) Validation of the Persian translation of the swallowing disturbance questionnaire in Parkinson's disease patients. *Parkinsons Dis* **2014**,.

[53] Yamamoto T, Ikeda K, Usui H, Miyamoto M, Murata M (2012) Validation of the japanese translation of the swallowing disturbance questionnaire in parkinson's disease patients. *Qual Life Res* **21**, 1299–1303.

[54] Mamolar Andrés S, Santamarina Rabanal ML, Granda Membiela CM, Fernández

Gutiérrez MJ, Sirgo Rodríguez P, Álvarez Marcos C (2017) Swallowing disorders in Parkinson’s disease. *Acta Otorrinolaringológica Española* **68**, 15–22.

[55] Presotto M, Olchik MR, Kalf JG, Rieder CRM (2018) Translation, linguistic and cultural adaptation, reliability and validity of the Radboud oral motor inventory for Parkinson’s disease – ROMP questionnaire. *Arq Neuropsiquiatr* **76**, 316–323.

[56] Nascimento D, Carmona J, Mestre T, Ferreira JJ, Guimarães I (2021) Drooling rating scales in Parkinson’s disease: A systematic review. *Park Relat Disord* **91**, 173–180.

[57] Antonini A, Martinez-Martin P, Chaudhuri RK, Merello M, Hauser R, Katzenschlager R, Odin P, Stacy M, Stocchi F, Poewe W, Rascol O, Sampaio C, Schrag A, Stebbins GT, Goetz CG (2011) Wearing-off scales in Parkinson’s disease: Critique and recommendations. *Mov Disord* **26**, 2169–2175.

[58] Hauser RA, Friedlander J, Zesiewicz TA, Adler CH, Seeberger LC, O’Brien CF, Molho ES, Factor SA (2000) A home diary to assess functional status in patients with Parkinson’s disease with motor fluctuations and dyskinesia. *Clin Neuropharmacol* **23**, 75–81.

[59] Hauser RA, Deckers F, Lehert P (2004) Parkinson’s disease home diary: Further validation amd implications for clinical trials. *Mov Disord* **19**, 1409–1413.

[60] Hauser RA, Russ H, Haeger DA, Bruguiere-Fontenille M, Müller T, Wenning GK (2006) Patient evaluation of a home diary to assess duration and severity of dyskinesia in parkinson disease. *Clin Neuropharmacol* **29**, 322–330.

[61] Reimer J, Grabowski M, Lindvall O, Hagell P (2004) Use and interpretation of on/off diaries in Parkinson’s disease. *J Neurol Neurosurg Psychiatry* **75**, 396–400.

[62] Stacy M, Hauser R, Oertel W, Schapira A, Sethi K, Stocchi F, Tolosa E (2006)

End-of-dose wearing off in parkinson disease: A 9-question survey assessment. *Clin Neuropharmacol* **29**, 312–321.

[63] Stacy M, Hauser R (2007) Development of a patient questionnaire to facilitate recognition of motor and non-motor wearing-off in Parkinson's disease. *J Neural Transm* **114**, 211–217.

[64] Stacy M, Bowron A, Guttman M, Hauser R, Hughes K, Larsen JP, Le Witt P, Oertel W, Quinn N, Sethi K, Stocchi F (2005) Identification of motor and nonmotor wearing-off in Parkinson's disease: Comparison of a patient questionnaire versus a clinician assessment. *Mov Disord* **20**, 726–733.

[65] Martinez-Martin P, Tolosa E, Hernandez B, Badia X (2008) Validation of the "QUICK" Questionnaire - A tool for diagnosis of "wearing-off" in patients with Parkinson's disease. *Mov Disord* **23**, 830–836.

[66] Mantese CE, Schumacher-Schuh A, Rieder CRM (2018) Clinimetrics of the 9-And 19-item wearing-off questionnaire: A systematic review. *Parkinsons Dis* **2018**,.

[67] Abbruzzese G, Antonini A, Barone P, Stocchi F, Tamburini T, Bernardi L, Amboni M, Vacca L, Posocco V, Colombo D (2012) Linguistic, psychometric validation and diagnostic ability assessment of an Italian version of a 19-item wearing-off questionnaire for wearing-off detection in Parkinson's disease. *Neurol Sci* **33**, 1319–1327.

[68] Pistacchi M, Gioulis M, Sanson F, Marsala S (2017) Wearing off: A complex phenomenon often poorly recognized in Parkinson's disease. A study with the WOQ-19 questionnaire. *Neurol India* **65**, 1271–1279.

[69] Tolosa E, Hernández B, Linazasoro G, López-Lozano JJ, Mir P, Marey J, Kulisevsky J (2014) Efficacy of levodopa/carbidopa/entacapone versus

levodopa/carbidopa in patients with early Parkinson’s disease experiencing mild wearing-off: A randomised, double-blind trial. *J Neural Transm* **121**, 357–366.

[70] Chaudhuri KR, Martinez-Martin P, Schapira AHV, Stocchi F, Sethi K, Odin P, Brown RG, Koller W, Barone P, MacPhee G, Kelly L, Rabey M, MacMahon D, Thomas S, Ondo W, Rye D, Forbes A, Tluk S, Dhawan V, Bowron A, Williams AJ, Olanow CW (2006) International multicenter pilot study of the first comprehensive self-completed nonmotor symptoms questionnaire for Parkinson’s disease: The NMSQuest study. *Mov Disord* **21**, 916–923.

[71] Chaudhuri KR, Sauerbier A, Rojo JM, Sethi K, Schapira AHV, Brown RG, Antonini A, Stocchi F, Odin P, Bhattacharya K, Tsuboi Y, Abe K, Rizos A, Rodriguez-Blazquez C, Martinez-Martin P (2015) The burden of non-motor symptoms in Parkinson’s disease using a self-completed non-motor questionnaire: A simple grading system. *Park Relat Disord* **21**, 287–291.

[72] Cova I, Di Battista ME, Vanacore N, Papi CP, Alampi G, Rubino A, Valente M, Meco G, Contri P, Di Pucchio A, Lacorte E, Priori A, Mariani C, Pomati S (2017) Adaptation and psychometric properties of the Italian version of the Non-Motor Symptoms Questionnaire for Parkinson’s disease. *Neurol Sci* **38**, 673–678.

[73] Li HJ, Zhang MF, Chen MX, Hu AL, Li J Bin, Zhang B, Liu W (2015) Validation of the nonmotor symptoms questionnaire for Parkinson’s disease: Results from a Chinese pilot study. *Int J Neurosci* **125**, 929–935.

[74] Rios Romenets S, Wolfson C, Galatas C, Pelletier A, Altman R, Wadup L, Postuma RB (2012) Validation of the non-motor symptoms questionnaire (NMS-Quest). *Park Relat Disord* **18**, 54–58.

[75] Chaudhuri KR, Martinez-Martin P, Brown RG, Sethi K, Stocchi F, Odin P, Ondo

W, Abe K, MacPhee G, MacMahon D, Barone P, Rabey M, Forbes A, Breen K, Tluk S, Naidu Y, Olanow W, Williams AJ, Thomas S, Rye D, Tsuboi Y, Hand A, Schapira AHV (2007) The metric properties of a novel non-motor symptoms scale for Parkinson's disease: Results from an international pilot study. *Mov Disord* **22**, 1901–1911.

[76] van Wamelen DJ, Martinez-Martin P, Weintraub D, Schrag A, Antonini A, Falup-Pecurariu C, Odin P, Ray Chaudhuri K (2021) The Non-Motor Symptoms Scale in Parkinson's disease: Validation and use. *Acta Neurol Scand* **143**, 3–12.

[77] van Wamelen DJ, Sauerbier A, Leta V, Rodriguez-Blazquez C, Falup-Pecurariu C, Rodriguez-Violante M, Rizos A, Tsuboi Y, Metta V, Bhidayasiri R, Bhattacharya K, Borgohain R, Prashanth LK, Rosales R, Lewis S, Fung V, Behari M, Goyal V, Kishore A, Lloret SP, Martinez-Martin P, Chaudhuri KR (2021) Cross-sectional analysis of the Parkinson's disease Non-motor International Longitudinal Study baseline non-motor characteristics, geographical distribution and impact on quality of life. *Sci Rep* **11**, 1–11.

[78] Martinez-Martin P, Schrag A, Weintraub D, Rizos A, Rodriguez-Blazquez C, Chaudhuri KR (2019) Pilot Study of the International Parkinson and Movement Disorder Society-sponsored Non-motor Rating Scale (MDS-NMS). *Mov Disord Clin Pract* **6**, 227–234.

[79] Chaudhuri KR, Schrag A, Weintraub D, Rizos A, Rodriguez-Blazquez C, Mamikonyan E, Martinez-Martin P (2020) The movement disorder society nonmotor rating scale: Initial validation study. *Mov Disord* **35**, 116–133.

[80] Martinez-Martin P, Leentjens AFG, de Pedro-Cuesta J, Chaudhuri KR, Schrag AE, Weintraub D (2016) Accuracy of screening instruments for detection of

neuropsychiatric syndromes in Parkinson's disease. *Mov Disord* **31**, 270–279.

[81] Krupp LB, Larocca NG, Muir Nash J, Steinberg AD (1989) The fatigue severity scale: Application to patients with multiple sclerosis and systemic lupus erythematosus. *Arch Neurol* **46**, 1121–1123.

[82] Tyson SF, Brown P (2014) How to measure fatigue in neurological conditions? A systematic review of psychometric properties and clinical utility of measures used so far. *Clin Rehabil* **28**, 804–816.

[83] Friedman JH, Alves G, Hagell P, Marinus J, Marsh L, Martinez-Martin P, Goetz CG, Poewe W, Rascol O, Sampaio C, Stebbins G, Schrag A (2010) Fatigue rating scales critique and recommendations by the Movement Disorders Society Task Force on rating scales for Parkinson's disease. *Mov Disord* **25**, 805–822.

[84] Lerdal A, Moum T, Wahl AK, Rustøen T, Hanestad BR (2005) Fatigue in the general population: A translation and test of the psychometric properties of the Norwegian version of the fatigue severity scale. *Scand J Public Health* **33**, 123–130.

[85] Chaudhuri KR, Rizos A, Trenkwalder C, Rascol O, Pal S, Martino D, Carroll C, Paviour D, Falup-Pecurariu C, Kessel B, Silverdale M, Todorova A, Sauerbier A, Odin P, Antonini A, Martinez-Martin P (2015) King's Parkinson's disease pain scale, the first scale for pain in PD: An international validation. *Mov Disord* **30**, 1623–1631.

[86] Perez-Lloret S, Ciampi de Andrade D, Lyons KE, Rodríguez-Blázquez C, Chaudhuri KR, Deuschl G, Cruccu G, Sampaio C, Goetz CG, Schrag A, Martinez-Martin P, Stebbins G, Cubo E, Hall D, Luo S, Marinus J, Marsh L, Skorvanek M (2016) Rating Scales for Pain in Parkinson's Disease: Critique and

Recommendations. *Mov Disord Clin Pract* **3**, 527–537.

[87] Rukavina K, Batzu L, Leta V, Chaudhuri KR (2022) New approaches to treatments for sleep, pain and autonomic failure in Parkinson’s disease - Pharmacological therapies. *Neuropharmacology* **208**, 108959.

[88] Taghizadeh G, Joghataei MT, Goudarzi S, Bakhsheshi M, Habibi SAH, Mehdizadeh M (2021) King’s Parkinson’s disease pain scale cut-off points for detection of pain severity levels: A reliability and validity study. *Neurosci Lett* **745**, 135620.

[89] Högl B, Arnulf I, Comella C, Ferreira J, Iranzo A, Tilley B, Trenkwalder C, Poewe W, Rascol O, Sampaio C, Stebbins GT, Schrag A, Goetz CG (2010) Scales to assess sleep impairment in Parkinson’s disease: Critique and recommendations. *Mov Disord* **25**, 2704–2716.

[90] Trenkwalder C, Kohnen R, Högl B, Metta V, Sixel-Döring F, Frauscher B, Hülsmann J, Martinez-Martin P, Chaudhuri KR (2011) Parkinson’s disease sleep scale-validation of the revised version PDSS-2. *Mov Disord* **26**, 644–652.

[91] Zea-Sevilla MA, Martínez-Martín P (2014) Rating scales and questionnaires for assessment of sleep disorders in Parkinson’s disease: What they inform about? *J Neural Transm* **121**,.

[92] Trenkwalder C, Kies B, Rudzinska M, Fine J, Nikl J, Honczarenko K, Dioszeghy P, Hill D, Anderson T, Myllyla V, Kassubek J, Steiger M, Zucconi M, Tolosa E, Poewe W, Surmann E, Whitesides J, Boroojerdi B, Chaudhuri KR (2011) Rotigotine effects on early morning motor function and sleep in Parkinson’s disease: A double-blind, randomized, placebo-controlled study (RECOVER). *Mov Disord* **26**, 90–99.

[93] Zibetti M, Rizzone M, Merola A, Angrisano S, Rizzi L, Montanaro E, Cicolin A, Lopiano L (2013) Sleep improvement with levodopa/carbidopa intestinal gel infusion in Parkinson disease. *Acta Neurol Scand* **127**, 28–32.

[94] Muntean ML, Benes H, Sixel-Döring F, Chaudhuri KR, Suzuki K, Hirata K, Zimmermann J, Trenkwalder C (2016) Clinically relevant cut-off values for the Parkinson's Disease Sleep Scale-2 (PDSS-2): a validation study. *Sleep Med* **24**, 87–92.

[95] Johns MW (1991) A new method for measuring daytime sleepiness: The Epworth sleepiness scale. *Sleep* **14**, 540–545.

[96] Schrag A, Barone P, Brown RG, Leentjens AFG, McDonald WM, Starkstein S, Weintraub D, Poewe W, Rascol O, Sampaio C, Stebbins GT, Goetz CG (2007) Depression rating scales in Parkinson's disease: Critique and recommendations. *Mov Disord* **22**, 1077–1092.

[97] Williams JR, Hirsch ES, Anderson K, Bush AL, Goldstein SR, Grill S, Lehmann S, Little JT, Margolis RL, Palanci J, Pontone G, Weiss H, Rabins P, Marsh L (2012) A comparison of nine scales to detect depression in Parkinson disease: Which scale to use? *Neurology* **78**, 998–1006.

[98] Yesavage JA, Brink TL, Rose TL, Lum O, Huang V, Adey M, Leirer VO (1982) Development and validation of a geriatric depression screening scale: A preliminary report. *J Psychiatr Res* **17**, 37–49.

[99] Ertan FS, Ertan T, Kiziltan G, Uyguçgil H (2005) Reliability and validity of the Geriatric Depression Scale in depression in Parkinson's disease. *J Neurol Neurosurg Psychiatry* **76**, 1445–1447.

[100] McDonald WM, Holtzheimer PE, Haber M, Vitek JL, McWhorter K, DeLong M

(2006) Validity of the 30-item geriatric depression scale in patients with Parkinson's disease. *Mov Disord* **21**, 1618–1622.

[101] Santangelo G, Vitale C, Trojano L, Picillo M, Moccia M, Pisano G, Pezzella D, Cuoco S, Erro R, Longo K, Pellecchia MT, Amboni M, De Rosa A, De Michele G, Barone P (2015) Relationship between apathy and cognitive dysfunctions in de novo untreated Parkinson's disease: A prospective longitudinal study. *Eur J Neurol* **22**, 253–260.

[102] Weintraub D, Saboe K, Stern MB (2007) Effect of age on geriatric depression scale performance in Parkinson's disease. *Mov Disord* **15**, 1331–1335.

[103] Kroenke K, Spitzer RL, Williams JBW (2001) The PHQ-9: Validity of a Brief Depression Severity Measure. *J Gen Intern Med* **16**, 606–613.

[104] Thompson AW, Liu H, Hays RD, Katon WJ, Rausch R, Diaz N, Jacob EL, Vassar SD, Vickrey BG (2011) Diagnostic accuracy and agreement across three depression assessment measures for Parkinson's disease. *Park Relat Disord* **17**, 40–45.

[105] New York, NY: The Research Foundation for Mental Hygiene, Columbia-Suicide Severity Rating Scale (C-SSRS), Last updated 2008, Accessed on 2008.

[106] Posner K, Brown GK, Stanley B, Brent DA, Yershova K V., Oquendo MA, Currier GW, Melvin GA, Greenhill L, Shen S, Mann JJ (2011) The Columbia-suicide severity rating scale: Initial validity and internal consistency findings from three multisite studies with adolescents and adults. *Am J Psychiatry* **168**, 1266–1277.

[107] Mohammad D, Ellis C, Rau A, Ruthirakuhan M, Lanctôt KL, Herrmann N (2018) Psychometric properties of apathy scales in Parkinson's disease: a systematic review. *Neurodegener Dis Manag* **8**, 267–282.

[108] Starkstein SE, Mayberg HS, Preziosi TJ, Andrezejewski P, Leiguarda R, Robinson RG (1992) Reliability, validity, and clinical correlates of apathy in Parkinson's disease. *J Neuropsychiatry Clin Neurosci* **4**, 134–139.

[109] Leentjens AFG, Dujardin K, Marsh L, Martinez-Martin P, Richard IH, Starkstein SE, Weintraub D, Sampaio C, Poewe W, Rascol O, Stebbins GT, Goetz CG (2008) Apathy and anhedonia rating scales in Parkinson's disease: Critique and recommendations. *Mov Disord* **23**, 2004–2014.

[110] Pedersen KF, Alves G, Larsen JP, Tysnes OB, Møller SG, Brønnick K (2012) Psychometric properties of the starkstein apathy scale in patients with early untreated Parkinson disease. *Am J Geriatr Psychiatry* **20**, 142–148.

[111] Kay DB, Kirsch-Darrow L, Zahodne LB, Okun MS, Bowers D (2012) Dimensions of apathy in Parkinson's disease: Exploratory factor analysis of the apathy scale. *J Parkinsons Dis* **2**, 161–166.

[112] Lopez F V., Eglit GML, Schiehser DM, Pirogovsky-Turk E, Litvan I, Lessig S, Filoteo JV (2019) Factor Analysis of the Apathy Scale in Parkinson's Disease. *Mov Disord Clin Pract* **6**, 379–386.

[113] Marin RS, Biedrzycki RC, Firinciogullari S (1991) Reliability and validity of the Apathy Evaluation Scale. *Psychiatry Res* **38**, 143–162.

[114] Lazcano-Ocampo C, Wan YM, van Wamelen DJ, Batzu L, Boura I, Titova N, Leta V, Qamar M, Martinez-Martin P, Ray Chaudhuri K (2020) Identifying and responding to fatigue and apathy in Parkinson's disease: a review of current practice. *Expert Rev Neurother* **20**, 477–495.

[115] Santangelo G, Barone P, Cuoco S, Raimo S, Pezzella D, Picillo M, Erro R, Moccia M, Pellecchia MT, Amboni M, Franco S, Grossi D, Trojano L, Vitale C (2014)

Apathy in untreated, de novo patients with Parkinson's disease: validation study of Apathy Evaluation Scale. *J Neurol* **261**, 2319–2328.

[116] Sockeel P, Dujardin K, Devos D, Denève C, Destée A, Defebvre L (2006) The Lille apathy rating scale (LARS), a new instrument for detecting and quantifying apathy: Validation in Parkinson's disease. *J Neurol Neurosurg Psychiatry* **77**, 579–584.

[117] Fernandez HH, Aarsland D, Fénelon G, Friedman JH, Marsh L, Tröster AI, Poewe W, Rascol O, Sampaio C, Stebbins GT, Goetz CG (2008) Scales to assess psychosis in Parkinson's disease: Critique and recommendations. *Mov Disord* **23**, 484–500.

[118] Voss TS, Brocht AFD, Ravina B (2010) Performance of the Scale for Assessment of Positive Symptoms in Parkinson's Disease Psychosis. *Mov Disord* **25**, 124–125.

[119] Voss T, Bahr D, Cummings J, Mills R, Ravina B, Williams H (2013) Performance of a shortened Scale for Assessment of Positive Symptoms for Parkinson's disease psychosis. *Park Relat Disord* **19**, 295–299.

[120] Kulick C V., Montgomery KM, Nirenberg MJ (2018) Comprehensive identification of delusions and olfactory, tactile, gustatory, and minor hallucinations in Parkinson's disease psychosis. *Park Relat Disord* **54**, 40–45.

[121] Pavy-Le Traon A, Amarenco G, Duerr S, Kaufmann H, Lahrmann H, Shaftman SR, Tison F, Wenning GK, Goetz CG, Poewe W, Sampaio C, Schrag A, Stebbins GT, Rascol O (2011) The Movement Disorders task force review of dysautonomia rating scales in Parkinson's disease with regard to symptoms of orthostatic hypotension. *Mov Disord* **26**, 1985–1992.

[122] Visser M, Marinus J, Stiggelbout AM, van Hilten JJ (2004) Assessment of

autonomic dysfunction in Parkinson's disease: The SCOPA-AUT. *Mov Disord* **19**, 1306–1312.

[123] Rodriguez-Blazquez C, Forjaz MJ, Frades-Payo B, De Pedro-Cuesta J, Martinez-Martin P (2010) Independent validation of the scales for outcomes in Parkinson's disease-autonomic (SCOPA-AUT). *Eur J Neurol* **17**, 194–201.

[124] Skorvanek M, Goldman JG, Jahanshahi M, Marras C, Rektorova I, Schmand B, van Duijn E, Goetz CG, Weintraub D, Stebbins GT, Martinez-Martin P (2018) Global scales for cognitive screening in Parkinson's disease: Critique and recommendations. *Mov Disord* **33**, 208–218.

[125] Nasreddine ZS, Phillips NA, Bédirian V, Charbonneau S, Whitehead V, Collin I, Cummings JL, Chertkow H (2005) The Montreal Cognitive Assessment, MoCA: A brief screening tool for mild cognitive impairment. *J Am Geriatr Soc* **53**, 695–699.

[126] Pagan FL, Wilmarth B, Torres-Yaghi Y, Hebron ML, Mulki S, Ferrante D, Matar S, Ahn J, Moussa C (2021) Long-Term Safety and Clinical Effects of Nilotinib in Parkinson's Disease. *Mov Disord* **36**, 740–749.

[127] Gill DJ, Freshman A, Blender JA, Ravina B (2008) The Montreal Cognitive Assessment as a screening tool for cognitive impairment in Parkinson's disease. *Mov Disord* **23**, 1043–1046.

[128] Hoops S, Nazem S, Siderowf AD, Duda JE, Xie SX, Stern MB, Weintraub D (2009) Validity of the MoCA and MMSE in the detection of MCI and dementia in parkinson disease. *Neurology* **73**, 1738–1745.

[129] Dalrymple-Alford JC, MacAskill MR, Nakas CT, Livingston L, Graham C, Crucian GP, Melzer TR, Kirwan J, Keenan R, Wells S, Porter RJ, Watts R,

Anderson TJ (2010) The MoCA: Well-suited screen for cognitive impairment in parkinson disease. *Neurology* **75**, 1717–1725.

[130] Mazancova AF, Růžička E, Jech R, Bezdicek O (2020) Test the best: Classification accuracies of four cognitive rating scales for Parkinson's disease mild cognitive impairment. *Arch Clin Neuropsychol* **35**, 1069–1077.

[131] Lucza T, Karádi K, Kállai J, Weintraut R, Janszky J, Makkos A, Komoly S, Kovács N (2015) Screening mild and major neurocognitive disorders in Parkinson's disease. *Behav Neurol* **2015**,.

[132] Caspell-Garcia C, Simuni T, Tosun-Turgut D, Wu IW, Zhang Y, Nalls M, Singleton A, Shaw LA, Kang JH, Trojanowski JQ, Siderowf A, Coffey C, Lasch S, Aarsland D, Burn D, Chahine LM, Espay AJ, Foster ED, Hawkins KA, Litvan I, Richard I, Weintraub D (2017) Multiple modality biomarker prediction of cognitive impairment in prospectively followed de novo Parkinson disease. *PLoS One* **12**, 1–18.

[133] Chen L, Yu C, Zhang N, Liu J, Liu W (2016) Cognitive impairment in patients with Parkinson's disease: A 30-month follow-up study. *Clin Neurol Neurosurg* **151**, 65–69.

[134] Matteau E, Dupré N, Langlois M, Provencher P, Simard M (2012) Clinical validity of the Mattis dementia rating scale-2 in Parkinson disease with MCI and dementia. *J Geriatr Psychiatry Neurol* **25**, 100–106.

[135] Dujardin K, Devos D, Duhem S, Destée A, Marié RM, Durif F, Lacomblez L, Touchon J, Pollak P, Péré JJ (2006) Utility of the Mattis dementia rating scale to assess the efficacy of rivastigmine in dementia associated with Parkinson's disease. *J Neurol* **253**, 1154–1159.

[136] Bezdicek O, Michalec J, Nikolai T, Havránková P, Roth J, Jech R, Růžička E (2015) Clinical validity of the mattis dementia rating scale in differentiating mild cognitive impairment in parkinson's disease and normative data. *Dement Geriatr Cogn Disord* **39**, 303–311.

[137] Siderowf A, Xie SX, Hurtig H, Weintraub D, Duda J, Chen-Plotkin A, Shaw LM, Van Deerlin V, Trojanowski JQ, Clark C (2010) CSF amyloid β 1-42 predicts cognitive decline in Parkinson disease. *Neurology* **75**, 1055–1061.

[138] Fernández de Bobadilla R, Pagonabarraga J, Martínez-Horta S, Pascual-Sedano B, Campolongo A, Kulisevsky J (2013) Parkinson's disease-cognitive rating scale: Psychometrics for mild cognitive impairment. *Mov Disord* **28**, 1376–1383.

[139] Pagonabarraga J, Kulisevsky J, Llebaria G, García-Sánchez C, Pascual-Sedano B, Gironell A (2008) Parkinson's disease-cognitive rating scale: A new cognitive scale specific for Parkinson's disease. *Mov Disord* **23**, 998–1005.

[140] Serrano-Dueñas M, Serrano M, Villena D, Granda D (2017) Validation of the Parkinson's Disease-Cognitive Rating Scale Applying the Movement Disorder Society Task Force Criteria for Dementia Associated with Parkinson's Disease. *Mov Disord Clin Pract* **4**, 51–57.

[141] Greenland JC, Cutting E, Kadyan S, Bond S, Chhabra A, Williams-Gray CH (2020) Azathioprine immunosuppression and disease modification in Parkinson's disease (AZA-PD): a randomised double-blind placebo-controlled phase II trial protocol. *BMJ Open* **10**, e040527.

[142] McColgan P, Evans JR, Breen DP, Mason SL, Barker RA, Williams-Gray CH (2012) Addenbrooke's Cognitive Examination-Revised for mild cognitive impairment in Parkinson's disease. *Mov Disord* **27**, 1173–1177.

[143] Reyes MA, Lloret SP, Gerscovich ER, Martin ME, Leiguarda R, Merello M (2009) Addenbrooke's Cognitive Examination validation in Parkinson's disease. *Eur J Neurol* **16**, 142–147.

[144] Berankova D, Janousova E, Mrackova M, Eliasova I, Kostalova M, Skutilova S, Rektorova I (2015) Addenbrooke's cognitive examination and individual domain cut-off scores for discriminating between different cognitive subtypes of Parkinson's disease. *Parkinsons Dis* **2015**,.

[145] Rittman T, Ghosh BC, McColgan P, Breen DP, Evans J, Williams-Gray CH, Barker RA, Rowe JB (2013) The Addenbrooke's Cognitive Examination for the differential diagnosis and longitudinal assessment of patients with parkinsonian disorders. *J Neurol Neurosurg Psychiatry* **84**, 544–551.

[146] Mioshi E, Dawson K, Mitchell J, Arnold R, Hodges JR (2006) The Addenbrooke's Cognitive Examination revised (ACE-R): A brief cognitive test battery for dementia screening. *Int J Geriatr Psychiatry* **21**, 1078–1085.

[147] Hsieh S, Schubert S, Hoon C, Mioshi E, Hodges JR (2013) Validation of the Addenbrooke's Cognitive Examination III in Frontotemporal Dementia and Alzheimer's Disease. *Dement Geriatr Cogn Disord* **36**, 242–250.

[148] Rosen WG, Mohs RC, Davis KL (1984) A new rating scale for Alzheimer's disease. *Am J Psychiatry* **141**, 1356–1364.

[149] Skorvanek M, Goldman JG, Jahanshahi M, Marras C (2018) Global Scales for Cognitive Screening in Parkinson ' s Disease : Critique and Recommendations Organization and Review Process. **33**,.

[150] Folstein MF, Folstein SE, McHugh PR (1975) "Mini-mental state". A practical method for grading the cognitive state of patients for the clinician. *J Psychiatr Res*

12, 189–198.

[151] Dubois B, Burn D, Goetz C, Aarsland D, Brown RG, Broe GA, Dickson D, Duyckaerts C, Cummings J, Gauthier S, Korczyn A, Lees A, Levy R, Litvan I, Mizuno Y, McKeith IG, Olanow CW, Poewe W, Sampaio C, Tolosa E, Emre M (2007) Diagnostic procedures for Parkinson’s disease dementia: Recommendations from the Movement Disorder Society Task Force. *Mov Disord* **22**, 2314–2324.

[152] Salmon DP, Thal LJ, Butters N, Heindel WC (1990) Longitudinal evaluation of dementia of the Alzheimer type: A comparison of 3 standardized mental status examinations. *Neurology* **40**, 1225–1230.

[153] Mahieux F, Michelet D, Manifacier M-J, Boller F, Fermanian J, Guillard A (1995) Mini-Mental Parkinson: First Validation Study of a New Bedside Test Constructed for Parkinson’s Disease. *Behav Neurol* **8**, 15–22.

[154] Serrano-Dueñas M, Calero B, Serrano S, Serrano M, Coronel P (2010) Metric properties of the mini-mental Parkinson and SCOPA-COG scales for rating cognitive deterioration in Parkinson’s disease. *Mov Disord* **25**, 2555–2562.

[155] Marinus J, Visser M, Na V, Fr V, Ha M, Am S, Hilten V (2003) Assessment of cognition in Parkinson’s disease. *Neurology* **61**, 1222–1228.

[156] National Institute of Neurological Disorders and Stroke (2022, August 31). Parkinson’s Disease Version 2.0 NINDS CDE Project Cognitive Subgroup Summary. Retrieved 12/01/2023 from: https://www.commondataelements.ninds.nih.gov/sites/nindscde/files/Doc/PD/F3.

[157] Williams-Gray CH, Mason SL, Evans JR, Foltynie T, Brayne C, Robbins TW, Barker RA (2013) The CamPaIGN study of Parkinson’s disease: 10-year outlook

in an incident population-based cohort. *J Neurol Neurosurg Psychiatry* **84**, 1258–1264.

[158] Anang JBM, Gagnon J, Bertrand J, Romentes SR, Latreille V, Passinet M, Montplaisir J, Postuma RB (2014) Predictors of dementia in Parkinson disease. *Neurology* **83**, 1253–1260.

[159] Zarkali A, McColgan P, Leyland LA, Lees AJ, Weil RS (2021) Visual Dysfunction Predicts Cognitive Impairment and White Matter Degeneration in Parkinson's Disease. *Mov Disord* **36**, 1191–1202.

[160] Benton AL, Varney N, Hamsher K DeS (1978) Visuospatial Judgement. A Clinical Test. *Arch Neurol* **35**, 364–367.

[161] Johnstone B, Wilhelm KL (1997) The Construct Validity of the Hooper Visual Organization Test. *Assessment* **4**, 243–248.

[162] Shulman LM, Armstrong M, Ellis T, Gruber-Baldini A, Horak F, Nieuwboer A, Parashos S, Post B, Rogers M, Siderowf A, Goetz CG, Schrag A, Stebbins GT, Martinez-Martin P (2016) Disability Rating Scales in Parkinson's Disease: Critique and Recommendations. *Mov Disord* **31**, 1455–1465.

[163] Bouça-Machado R, Fernandes A, Ranzato C, Beneby D, Nzwalo H, Ferreira JJ (2022) Measurement tools to assess activities of daily living in patients with Parkinson's disease: A systematic review. *Front Neurosci* **16**,.

[164] Schwab J, England A (1969) Projection technique for evaluating surgery in Parkinson's disease. In *Third Symposium on Parkinson's Disease. Vol. 232*, Gillingham F, Donaldson M, eds. Churchill Livingstone, Edinburgh, Scotland, pp. 152–157.

[165] McRae C, Diem G, Vo A, O'Brien C, Seeberger L (2000) Schwab & England-

standardization of administration McRae et al Mov Disord 2000.pdf. *Mov Disord* **15**, 335–336.

[166] Schrag A, Spottke A, Quinn NP, Dodel R (2009) Comparative responsiveness of Parkinson's disease scales to change over time. *Mov Disord* **24**, 813–818.

[167] Martinez-Martin P, Prieto L, Forjaz MJ (2006) Longitudinal metric properties of disability rating scales for Parkinson's disease. *Value Heal* **9**, 386–393.

[168] Bello-Haas VD, Klassen L, Sheppard S, Metcalfe A (2011) Psychometric properties of activity, self-efficacy and quality-of-life measures in individuals with parkinson disease. *Physiother Canada* **63**, 47–57.

[169] Jette AM, Davies AR, Cleary PD, Calkins DR, Rubenstein L V., Fink A, Kosecoff J, Young RT, Brook RH, Delbanco TL (1986) The functional status questionnaire: Reliability and validity when used in primary care. *J Gen Intern Med* **1**, 427–427.

[170] Rubenstein LM, Voelker MD, Chrischilles EA, Glenn DC, Wallace RB, Rodnitzky RL (1998) The usefulness of the Functional Status Questionnaire and Medical Outcomes Study Short Form in Parkinson's disease research. *Qual Life Res* **7**, 279–290.

[171] Clarke CE, Speller J, Clarke JA (2000) Pramipexole versus bromocriptine for levodopa-induced complications in Parkinson's disease. *Cochrane Database Syst Rev* **2000**,.

[172] Gallanagh S, Castagno D, Wilson B, Erdmann E, Zannad F, Remme WJ, Lopez-Sendon JL, Lechat P, Follath F, Höglund C, Mareev V, Sadowski Z, Seabra-Gomes RJ, Dargie HJ, McMurray JJV (2011) Evaluation of the functional status questionnaire in heart failure: A sub-study of the second Cardiac Insufficiency Bisoprolol Survival Study (CIBIS-II). *Cardiovasc Drugs Ther* **25**, 77–85.

[173] Cleary PD, Jette AM (2000) Reliability and validity of the functional status questionnaire. *Qual Life Res* **9**, 747–753.

[174] Al-Janabi H, Flynn TN, Coast J (2012) Development of a self-report measure of capability wellbeing for adults: The ICECAP-A. *Qual Life Res* **21**, 167–176.

[175] Coast J, Flynn TN, Natarajan L, Sproston K, Lewis J, Louviere JJ, Peters TJ (2008) Valuing the ICECAP capability index for older people. *Soc Sci Med* **67**, 874–882.

[176] Pillas M, Selai C, Quinn NP, Lees A, Litvan I, Lang A, Bower J, Burn D, Low P, Schrag A (2016) Development and validation of a carers quality-of-life questionnaire for parkinsonism (PQoL Carers). *Qual Life Res* **25**, 81–88.

[177] Jenkinson C, Dummett S, Kelly L, Peters M, Dawson J, Morley D, Fitzpatrick R (2012) The development and validation of a quality of life measure for the carers of people with Parkinson's disease (the PDQ-Carer). *Park Relat Disord* **18**, 483–487.

[178] Ferrer-Cascales R, Cabañero-Martínez MJ, Sánchez-SanSegundo M, Congost-Maestre N, Jenkinson C (2016) Spanish version of the Parkinson's Disease Questionnaire-Carer (PDQ-Carer). *Health Qual Life Outcomes* **14**, 1.

[179] Zarit SH, Reever KE, Bach-Peterson J (1980) Relatives of the impaired elderly: Correlates of feelings of burden. *Gerontologist* **20**, 649–655.

[180] Hagell P, Alvariza A, Westergren A, Årestedt K (2017) Assessment of Burden Among Family Caregivers of People With Parkinson's Disease Using the Zarit Burden Interview. *J Pain Symptom Manage* **53**, 272–278.

[181] Martinez-Martin P, Jeukens-Visser M, Lyons KE, Rodriguez-Blazquez C, Selai C, Siderowf A, Welsh M, Poewe W, Rascol O, Sampaio C, Stebbins GT, Goetz CG, Schrag A (2011) Health-related quality-of-life scales in Parkinson's disease:

Critique and recommendations. *Mov Disord* **26**, 2371–2380.

[182] Dodel R, Jönsson B, Reese JP, Winter Y, Martinez-Martin P, Holloway R, Sampaio C, Růžička E, Hawthorne G, Oertel W, Poewe W, Stebbins G, Rascol O, Goetz CG, Schrag A (2014) Measurement of costs and scales for outcome evaluation in health economic studies of Parkinson's disease. *Mov Disord* **29**, 169–176.

[183] (1990) The EuroQoL Group. EuroQol - a new facility for the measurement of health-related quality of life. *Health Policy (New York)* **16**, 199–208.

[184] Feng YS, Kohlmann T, Janssen MF, Buchholz I (2021) Psychometric properties of the EQ-5D-5L: a systematic review of the literature. *Qual Life Res* **30**, 647–673.

[185] Hunter RM, Baio G, Butt T, Morris S, Round J, Freemantle N (2015) An Educational Review of the Statistical Issues in Analysing Utility Data for Cost-Utility Analysis. *Pharmacoeconomics* **33**, 355–366.

[186] Ware JE, Sherbourne CD (1992) The MOS 36-item short-form health survey (Sf-36): I. conceptual framework and item selection. *Med Care* **30**, 473–483.

[187] Brown CA, Cheng EM, Hays RD, Vassar SD, Vickrey BG (2009) SF-36 includes less Parkinson Disease (PD)-targeted content but is more responsive to change than two PD-targeted health-related quality of life measures. *Qual Life Res* **18**, 1219–1237.

[188] Steffen T, Seney M (2008) Test-retest reliability and minimal detectable change on balance and ambulation tests, the 36-item short-form health survey, and the unified Parkinson disease rating scale in people with parkinsonism. *Phys Ther* **88**, 733–746.

[189] Brazier JE, Roberts J (2004) The estimation of a preference-based index from the

SF-12. *Med Care* **42**, 851–859.

[190] Ware JE, Kosinski M, Keller SD (1996) A 12-Item Short-Form Health Survey: Construction of Scales and Preliminary Tests of Reliability and Validity. *Med Care* **34**, 220–233.

[191] Furlong WJ, Feeny DH, Torrance GW, Barr RD (2001) The Health Utilities Index (HUI®) system for assessing health-related quality of life in clinical studies. *Ann Med* **33**, 375–384.

[192] Horsman J, Furlong W, Feeny D, Torrance G (2003) The Health Utilities Index (HUI®): concepts, measurement properties and applications. **13**, 1–13.

[193] Gershon RC, Lai JS, Bode R, Choi S, Moy C, Bleck T, Miller D, Peterman A, Cella D (2012) Neuro-QOL: Quality of life item banks for adults with neurological disorders: Item development and calibrations based upon clinical and general population testing. *Qual Life Res* **21**, 475–486.

[194] Rose M, Bjorner JB, Becker J, Fries JF, Ware JE (2008) Evaluation of a preliminary physical function item bank supported the expected advantages of the Patient-Reported Outcomes Measurement Information System (PROMIS). *J Clin Epidemiol* **61**, 17–33.

[195] Nowinski CJ, Siderowf A, Simuni T, Wortman C, Moy C, Cella D (2016) Neuro-QoL health-related quality of life measurement system: Validation in Parkinson's disease. *Mov Disord* **31**, 725–733.

[196] Shin JY, Pohlig RT, Habermann B (2018) Feasibility of Using PROMIS® in Individuals With Advanced Parkinson's Disease and Their Caregivers. *Res Gerontol Nurs* **11**, 129–136.

[197] Terwee CB, Peipert JD, Chapman R, Lai JS, Terluin B, Cella D, Griffith P,

Mokkink LB (2021) Minimal important change (MIC): a conceptual clarification and systematic review of MIC estimates of PROMIS measures. *Qual Life Res* **30**, 2729–2754.

[198] Kozlowski AJ, Cella D, Nitsch KP, Heinemann AW (2016) Evaluating Individual Change with the Quality of Life in Neurological Disorders (Neuro-QoL) Short Forms. *Arch Phys Med Rehabil* **97**, 650-654.e8.

[199] Jones J, Nielson SA, Trout J, Swenson M, Reiley J, Tanner J, Bowers D, Kay DB (2021) A Validation Study of PROMIS Sleep Disturbance (PROMIS-SD) and Sleep Related Impairment (PROMIS-SRI) Item Banks in Individuals with Idiopathic Parkinson's Disease and Matched Controls. *J Parkinsons Dis* **11**, 877–883.

[200] Berardi A, Regoli E, Tofani M, Valente D, Fabbrini G, Fabbrini A, Ruggieri M, Panuccio F, Galeoto G (2021) Tools to assess the quality of life in patients with Parkinson's disease: a systematic review. *Expert Rev Pharmacoeconomics Outcomes Res* **21**, 55–68.

[201] Peto V, Jenkinson C, Fitzpatrick R, Greenhall R (1995) The Development and Validation of a Short Measure of Functioning and Well Being for Individuals with Parkinson's Disease. *Qual Life Res* **4**, 241–248.

[202] Jenkinson C, Fitzpatrick R, Peto V, Greenhall R, Hyman N (1997) The PDQ-8: Development and validation of a short-form Parkinson's disease questionnaire. *Psychol Heal* **12**, 805–814.

[203] Marinus J, Ramaker C, van Hilten JJ, Stiggelbout AM (2002) Health related quality of life in Parkinson's disease: A systematic review of disease specific instruments. *J Neurol Neurosurg Psychiatry* **72**, 241–248.

[204] Clarke CE, Furmston A, Morgan E, Patel S, Sackley C, Walker M, Bryan S, Wheatley K (2009) Pilot randomised controlled trial of occupational therapy to optimise independence in Parkinson’s disease: The PD OT trial. *J Neurol Neurosurg Psychiatry* **80**, 976–978.

[205] Peto V, Jenkinson C, Fitzpatrick RAY (2001) Determining minimally important differences for the PDQ-39 Parkinson’s disease questionnaire. *Age Ageing* **30**, 299–302.

[206] Fitzpatrick R, Peto V, Jenkinson C, Greenhall R, Hyman N (1997) Health-related quality of life in Parkinson’s disease: A study of outpatient clinic attenders. *Mov Disord* **12**, 916–922.

[207] Jenkinson C, Fitzpatrick R, Peto V, Dummett S, Morley D, Saunders P (2018) *The Parkinson ’ s disease questionnaires user manual*.

[208] Dams J, Klotsche J, Bornschein B, Reese JP, Balzer-Geldsetzer M, Winter Y, Schrag A, Siderowf A, Oertel WH, Deuschl G, Siebert U, Dodel R (2013) Mapping the EQ-5D index by UPDRS and PDQ-8 in patients with Parkinson’s disease. *Health Qual Life Outcomes* **11**, 1–7.

[209] Luo N, Tan LCS, Zhao Y, Lau PN, Au WL, Li SC (2009) Determination of the longitudinal validity and minimally important difference of the 8-item Parkinson’s disease questionnaire (PDQ-8). *Mov Disord* **24**, 183–187.

[210] Huang TT, Hsu HY, Wang BH, Chen KH (2011) Quality of life in Parkinson’s disease patients: validation of the Short-Form Eight-item Parkinson’s Disease Questionnaire (PDQ-8) in Taiwan. *Qual Life Res* **20**, 499–505.

[211] Beecham J, Knapp M (2001) Costing psychiatric interventions. In *Measuring Mental Health Needs*, Thornicroft G, ed. Royal College of Psychiatrists, London,

pp. 200–224.

[212] McCrone P (2009) Capturing the Costs of End-of-Life Care: Comparisons of Multiple Sclerosis, Parkinson's Disease, and Dementia. *J Pain Symptom Manage* **38**, 62–67.

[213] Personal Social Services Research Unit. Client Service Receipt Inventory (CSRI). https://www.pssru.ac.uk/csri/client-service-receipt-inventory/.

[214] National Institute for Health and Care Excellence (2022) NICE health technology evaluations: the manual. 1–181.

[215] Parkinson-Study-Group (1989) DATATOP: A multicenter controlled clinical trial in early Parkinson's disease. *Arch Neurol* **46**, 1052–1060.

[216] Tosin MHS, Simuni T, Stebbins GT, Cedarbaum JM (2022) Tracking Emergence of New Motor and Non-Motor Symptoms Using the MDS-UPDRS: A Novel Outcome Measure for Early Parkinson's Disease? *J Parkinsons Dis* 1–7.

[217] Movement Disorders, Time-to-event approach mitigates the treatment masking effect of symptomatics on MDS-UPDRS Part III [abstract], Last updated 2022, Accessed on 2022.

[218] Gonzalez MC, Dalen I, Maple-Grødem J, Tysnes OB, Alves G (2022) Parkinson's disease clinical milestones and mortality. *npj Park Dis* **8**, 58.

[219] Simuni T, Siderowf A, Lasch S, Coffey CS, Caspell-Garcia C, Jennings D, Tanner CM, Trojanowski JQ, Shaw LM, Seibyl J, Schuff N, Singleton A, Kieburtz K, Toga AW, Mollenhauer B, Galasko D, Chahine LM, Weintraub D, Foroud T, Tosun D, Poston K, Arnedo V, Frasier M, Sherer T, Chowdhury S, Marek K (2018) Longitudinal Change of Clinical and Biological Measures in Early Parkinson's Disease: Parkinson's Progression Markers Initiative Cohort. *Mov Disord* **33**, 771–

782.

[220] Arora S, Baig F, Lo C, Barber TR, Lawton MA, Zhan A, Rolinski M, Ruffmann C, Klein JC, Rumbold J, Louvel A, Zaiwalla Z, Lennox G, Quinnell T, Dennis G, Wade-Martins R, Ben-Shlomo Y, Little MA, Hu MT (2018) Smartphone motor testing to distinguish idiopathic REM sleep behavior disorder, controls, and PD. *Neurology* **91**, E1528–E1538.

[221] Jha A, Menozzi E, Oyekan R, Latorre A, Mulroy E, Schreglmann SR, Stamate C, Daskalopoulos I, Kueppers S, Luchini M, Rothwell JC, Roussos G, Bhatia KP (2020) The CloudUPDRS smartphone software in Parkinson's study: cross-validation against blinded human raters. *npj Park Dis* **6**, 1–8.

[222] Hasegawa N, Shah V V., Carlson-Kuhta P, Nutt JG, Horak FB, Mancini M (2019) How to select balance measures sensitive to parkinson's disease from body-worn inertial sensors—separating the trees from the forest. *Sensors (Switzerland)* **19**,.

[223] Shah V V., McNames J, Mancini M, Carlson-Kuhta P, Spain RI, Nutt JG, El-Gohary M, Curtze C, Horak FB (2020) Quantity and quality of gait and turning in people with multiple sclerosis, Parkinson's disease and matched controls during daily living. *J Neurol* **267**, 1188–1196.

[224] Zhan A, Mohan S, Tarolli C, Schneider RB, Adams JL, Sharma S, Elson MJ, Spear KL, Glidden AM, Little MA, Terzis A, Ray Dorsey E, Saria S (2018) Using smartphones and machine learning to quantify Parkinson disease severity the mobile Parkinson disease score. *JAMA Neurol* **75**, 876–880.

[225] Griffiths RI, Kotschet K, Arfon S, Xu ZM, Johnson W, Drago J, Evans A, Kempster P, Raghav S, Horne MK (2012) Automated assessment of bradykinesia and dyskinesia in Parkinson's disease. *J Parkinsons Dis* **2**, 47–55.

[226] Powers R, Etezadi-Amoli M, Arnold EM, Kianian S, Mance I, Gibiansky M, Trietsch D, Alvarado AS, Kretlow JD, Herrington TM, Brillman S, Huang N, Lin PT, Pham HA, Ullal A V. (2021) Smartwatch inertial sensors continuously monitor real-world motor fluctuations in Parkinson's disease. *Sci Transl Med* **13**, 1–12.

[227] Rehman RZU, Zhou Y, Din S Del, Alcock L, Hansen C, Guan Y, Hortobágyi T, Maetzler W, Rochester L, Lamoth CJC (2020) Gait analysis with wearables can accurately classify fallers from non-fallers: A step toward better management of neurological disorders. *Sensors (Switzerland)* **20**, 1–17.

[228] Polhemus A, Ortiz LD, Brittain G, Chynkiamis N, Salis F, Gaßner H, Gross M, Kirk C, Rossanigo R, Taraldsen K, Balta D, Breuls S, Buttery S, Cardenas G, Endress C, Gugenhan J, Keogh A, Kluge F, Koch S, Micó-Amigo ME, Nerz C, Sieber C, Williams P, Bergquist R, de Basea MB, Buckley E, Hansen C, Mikolaizak AS, Schwickert L, Scott K, Stallforth S, van Uem J, Vereijken B, Cereatti A, Demeyer H, Hopkinson N, Maetzler W, Troosters T, Vogiatzis I, Yarnall A, Becker C, Garcia-Aymerich J, Leocani L, Mazzà C, Rochester L, Sharrack B, Frei A, Puhan M (2021) Walking on common ground: a cross-disciplinary scoping review on the clinical utility of digital mobility outcomes. *npj Digit Med* **4**,.

[229] Del Din S, Kirk C, Yarnall AJ, Rochester L, Hausdorff JM (2021) Body-Worn Sensors for Remote Monitoring of Parkinson's Disease Motor Symptoms: Vision, State of the Art, and Challenges Ahead. *J Parkinsons Dis* **11**, S35–S47.

[230] Pagano G, Boess FG, Taylor KI, Ricci B, Mollenhauer B, Poewe W, Boulay A, Anzures-Cabrera J, Vogt A, Marchesi M, Post A, Nikolcheva T, Kinney GG, Zago WM, Ness DK, Svoboda H, Britschgi M, Ostrowitzki S, Simuni T, Marek K, Koller M, Sevigny J, Doody R, Fontoura P, Umbricht D, Bonni A, Altendorf C,

Anandan C, Andrews G, Ansquer S, Arrouasse R, Aslam S, Azulay JP, Baker J, Martinez EB, Barbu S, Bardram K, Bega D, Bejr-Kasem Marco H, Benatru I, Benchetrit E, Bernhard F, Besharat A, Bette S, Bichon A, Billnitzer A, Blondeau S, Boraud T, Borngräber F, Boyd J, Brockmann K, Brodsky M, Brown E, Bruecke C, Calvas F, Canelo M, Carbone F, Carroll C, Fernandez LC, Cassé-Perrot C, Castrioto A, Catala H, Chan J, Cheriet S, Ciabarra A, Classen J, Coleman J, Coleman R, Compta Y, Corbillé AG, Corvol JC, Cosgaya M, Dahodwala N, Damier P, David E, Davis T, Dean M, Debilly B, DeGiorgio J, Deik A, Delaby L, Delfini MH, Derkinderen P, Derost P, de Toledo M, Deuel L, Diaz-Hernandez AM, Dietiker C, Dimenshteyn K, Dotor J, Durif F, Ebentheuer J, Eggert KM, Madueño SE, Eickhoff C, Ellenbogen A, Ellmerer P, Vazquez IE, Eusebio A, Ewert S, Fang J, Feigenbaum D, Fluchere F, Foubert-Samier A, Fournier M, Fradet A, Fraix V, Frank S, Fries F, Galitzky M, Pérez MG, Moreno JMG, Gasca C, Gasser T, Gibbons J, Giordana C, Martinez AG, Goodman I, Gorospe A, Goubeaud M, Grabli D, Graziella M, Grimaldi S, Gross J, Guimaraes-Costa R, Hartmann A, Hartmann C, Hassell T, Hauser R, Hernandez A, Hernandez-Vara J, Hoeglinger G, Homedes C, Horta-Barba A, Houeto JL, Huebl J, Hui J, Isaacson S, Jankovic J, Janzen A, Jauregui J, Jiao J, Domenech MJM, Joseph X, Kadimi S, Kaminski P, Kannenberg S, Kassubek J, Katz M, Klos K, Klos S, Kobet C, Koebert J, Krause P, Kuehn A, Bojarsky JK, Kumar R, Kunz M, Kurvits L, Kwei K, Laganiere S, Laurens B, Levin J, Levy O, LeWitt P, Cristóbal GL, Litvan I, Lizarraga K, Longardner K, Lopez R, Manzanares LL, del Pozo SL, Puido MRL, Luthra N, Lyons K, Maass S, Machetanz G, Macias Y, Maltete D, Manez Miro JU, Mariani LL, Marin J, Marini K, Marques A, Marti G, Domenech MJM, Martinez S, Meissner W, Meoni S, Mollenhauer B, Martinez DM, Moon J, Moro E,

Morrison P, Muehlberg C, Multani M, Murphy C, Nicholas A, Pahwa R, Palasi A, Pape H, Patel N, Patel P, Peball M, Peckham E, Peery T, Perez R, Perez J, Petit A, Pinkhardt E, Poewe W, Pomies E, Preterre C, Quinn J, Rascol O, Remy P, Richard I, Roeben B, Ruether E, Rumpf JJ, Russell D, Salhi H, Samaniego-Toro D, Samier-Foubert A, Sanchez A, Schmitt E, Schnitzler A, Schorr O, Schwartzbard J, Schweyer K, Seppi K, Sergo V, Shill H, Siderowf A, Simuni T, Spampinato U, Sriram A, Stover N, Tanner C, Tarakad A, Taylor C, Thalamus C, Toothaker T, Van Blercom N, Vanegas-Arrogave N, Vela L, Vergnet S, Vidal T, Vöglein J, Walsh R, Waters C, Wegscheider M, Weidinger E, Weill C, Wenzel G, Witjas T, Wurster I, Wright B, Zimmermann M, Zuzuarregui R, Abt M, Bamdadian A, Barata T, Barbet N, Belli S, Boess F, Bonni A, Borroni E, Boulay A, Britschgi M, Chague J, Cosson V, Czech C, Deptula D, Diack C, Doody R, Dukart J, D'Urso G, Dziadek S, Eddleston H, Edgar C, Essioux L, Farell M, Finch R, Fontoura P, Gruenbauer W, Hahn A, Holiga S, Honer M, Jadidi S, Johnson-Wood K, Keller M, Kilchenmann T, Koller M, Kremer T, Kustermann T, Landsdall C, Lindemann M, Lipsmeier F, Luzy C, Manchester M, Marchesi M, Martenyi F, Martin-Facklam M, Mironova K, Monnet A, Moore E, Ness DK, Niggli M, Nikolcheva T, Ostrowitzki S, Pagano G, Passmard B, Poirier A, Post A, Prasad M, Pross N, Quock T, Ricci B, Rose E, Sarry C, Schubert C, Selkoe D, Sevigny J, Sink K, Staunton H, Steven T, Strasak A, Svoboda H, Taylor K, Tripuraneni R, Trundell D, Umbricht D, Verselis L, Vogt A, Volkova-Volkmar E, Weber C, Weber S, Zago W (2021) A Phase II Study to Evaluate the Safety and Efficacy of Prasinezumab in Early Parkinson's Disease (PASADENA): Rationale, Design, and Baseline Data. *Front Neurol* **12**, 1–17.

[231] Lipsmeier F, Taylor KI, Postuma RB, Kilchenmann T, Wolf D, Zhang Y-P, Cheng

W-Y, Volkova-Volkmar E, Scotland A, Schjodt-Eriksen J, Boess F, Ness D, Gossens C, Post A, Lindemann M (2019) Preliminary validation of a novel, comprehensive digital biomarker smartphone application to assess motor symptoms in recently diagnosed Parkinson patients. *Neurology* **92 (15 Sup**, 4.7-005.

[232] Lipsmeier F, Taylor KI, Kilchenmann T, Wolf D, Scotland A, Schjodt-Eriksen J, Cheng WY, Fernandez-Garcia I, Siebourg-Polster J, Jin L, Soto J, Verselis L, Boess F, Koller M, Grundman M, Monsch AU, Postuma RB, Ghosh A, Kremer T, Czech C, Gossens C, Lindemann M (2018) Evaluation of smartphone-based testing to generate exploratory outcome measures in a phase 1 Parkinson's disease clinical trial. *Mov Disord* **33**, 1287–1297.

[233] Mikolaizak AS, Rochester L, Maetzler W, Sharrack B, Demeyer H, Mazzà C, Caulfield B, Garcia-Aymerich J, Vereijken B, Arnera V, Miller R, Piraino P, Ammour N, Gordon MF, Troosters T, Yarnall AJ, Alcock L, Gaßner H, Winkler J, Klucken J, Schlenstedt C, Watz H, Kirsten A-M, Vogiatzis I, Chynkiamis N, Hume E, Megaritis D, Nieuwboer A, Ginis P, Buckley E, Brittain G, Comi G, Leocani L, Helbostad JL, Johnsen LG, Taraldsen K, Blain H, Driss V, Frei A, Puhan MA, Polhemus A, Bosch de Basea M, Gimeno E, Hopkinson NS, Buttery SC, Hausdorff JM, Mirelman A, Evers J, Neatrour I, Singleton D, Schwickert L, Becker C, Jansen C-P, consortium members of the clinical validation study (WP4) on behalf of M-D (2022) Connecting real-world digital mobility assessment to clinical outcomes for regulatory and clinical endorsement – the Mobilise-D study protocol. *medRxiv* 2022.05.25.22275598.

[234] Podsiadlo D, Richardson S (1991) The timed "Up & Go": a test of basic functional mobility for frail elderly persons. *J Am Geriatr Soc* **39**, 142–148.

[235] Spagnuolo G, Faria CDCM, Da Silva BA, Ovando AC, Gomes-Osman J, Swarowsky A (2018) Are functional mobility tests responsive to group physical therapy intervention in individuals with Parkinson's disease? *NeuroRehabilitation* **42**, 465–472.

[236] Johnston M, De Morton N, Harding K, Taylor N (2013) Measuring mobility in patients living in the community with Parkinson disease. *NeuroRehabilitation* **32**, 957–966.

[237] Tiffin J, Asher EJ (1948) The Purdue Pegboard: norms and studies of reliability and validity. *J Appl Psychol* **32**, 234–247.

[238] Proud EL, Miller KJ, Bilney B, Morris ME, McGinley JL (2020) Construct validity of the 9-Hole Peg Test and Purdue Pegboard Test in people with mild to moderately severe Parkinson's disease. *Physiother (United Kingdom)* **107**, 202–208.

[239] Postuma RB, Iranzo A, Hu M, Högl B, Boeve BF, Manni R, Oertel WH, Arnulf I, Ferini-Strambi L, Puligheddu M, Antelmi E, Cochen De Cock V, Arnaldi D, Mollenhauer B, Videnovic A, Sonka K, Jung KY, Kunz D, Dauvilliers Y, Provini F, Lewis SJ, Buskova J, Pavlova M, Heidbreder A, Montplaisir JY, Santamaria J, Barber TR, Stefani A, Louis SEK, Terzaghi M, Janzen A, Leu-Semenescu S, Plazzi G, Nobili F, Sixel-Doering F, Dusek P, Bes F, Cortelli P, Ehgoetz Martens K, Gagnon JF, Gaig C, Zucconi M, Trenkwalder C, Gan-Or Z, Lo C, Rolinski M, Mahlknecht P, Holzknecht E, Boeve AR, Teigen LN, Toscano G, Mayer G, Morbelli S, Dawson B, Pelletier A (2019) Risk and predictors of dementia and parkinsonism in idiopathic REM sleep behaviour disorder: A multicentre study. *Brain* **142**, 744–759.

[240] Kawada T (2015) Predictors of dementia in Parkinson disease: A prospective cohort study. *Neurology* **84**, 1285.

[241] Nutt JG, Lea ES, Van Houten L, Schuff RA, Sexton GJ (2000) Determinants of tapping speed in normal control subjects and subjects with Parkinson's disease: Differing effects of brief and continued practice. *Mov Disord* **15**, 843–849.

[242] Boraud T, Tison F, Gross C (1997) Quantification of motor slowness in Parkinson's disease: Correlations between the tapping test and single joint ballistic movement parameters. *Park Relat Disord* **3**, 47–50.

[243] Burns BD, Dejong JD (1960) A preliminary report on the measurement of parkinson's disease. *Neurology* **10**, 1096–1102.

[244] Turjanski N, Fernandez W, Lees AJ (1993) The effects of acute levodopa withdrawal on motor performance and dopaminergic receptor sensitivity in patients with parkinson's disease. *J Neurol Neurosurg Psychiatry* **56**, 771–775.

[245] Hasan H, Burrows M, Athauda DS, Hellman B, James B, Warner T, Foltynie T, Giovannoni G, Lees AJ, Noyce AJ (2019) The BRadykinesia Akinesia INcoordination (BRAIN) Tap Test: Capturing the Sequence Effect. *Mov Disord Clin Pract* **6**, 462–469.

[246] Noyce AJ, Nagy A, Acharya S, Hadavi S, Bestwick JP, Fearnley J, Lees AJ, Giovannoni G (2014) Bradykinesia-akinesia incoordination test: Validating an online keyboard test of upper limb function. *PLoS One* **9**,.

[247] Earhart GM, Cavanaugh JT, Ellis T, Ford MP, Foreman KB, Dibble L (2011) The 9-hole peg test of upper extremity function: Average values, test-retest reliability, and factors contributing to performance in people with parkinson disease. *J Neurol Phys Ther* **35**, 157–163.

[248] Proud E, Morris ME, Bilney B, Miller KJ, Nijkrake MJ, Munneke M, McGinley JL (2021) Hand dexterity assessment in Parkinson's disease: construct validity of the 9-Hole peg test for the more affected hand. *Disabil Rehabil* **43**, 3834–3838.

[249] Lo C, Arora S, Lawton M, Barber T, Quinnell T, Dennis GJ, Shlomo YB-, Tao- M (2022) A composite clinical motor score as a comprehensive and sensitive outcome measure for Parkinson ' s disease. 1–8.

[250] Saeed U, Lang AE, Masellis M (2020) Neuroimaging Advances in Parkinson's Disease and Atypical Parkinsonian Syndromes. *Front Neurol* **11**,.

[251] Nicastro N, Nencha U, Burkhard PR, Garibotto V (2021) Dopaminergic imaging in degenerative parkinsonisms, an established clinical diagnostic tool. *J Neurochem* 1–18.

[252] Wang L, Zhang Q, Li H, Zhang H (2012) SPECT molecular imaging in Parkinson's disease. *J Biomed Biotechnol* **2012**,.

[253] Palermo G, Giannoni S, Bellini G, Siciliano G, Ceravolo R (2021) Dopamine transporter imaging, current status of a potential biomarker: A comprehensive review. *Int J Mol Sci* **22**,.

[254] Porter E, Roussakis AA, Lao-Kaim NP, Piccini P (2020) Multimodal dopamine transporter (DAT) imaging and magnetic resonance imaging (MRI) to characterise early Parkinson's disease. *Park Relat Disord* **79**, 26–33.

[255] Asenbaum S, Brücke T, Pirker W, Podreka I, Angelberger P, Wenger S, Wöber C, Müller C, Deecke L (1997) Imaging of dopamine transporters with iodine-123-β-CIT and SPECT in Parkinson's disease. *J Nucl Med* **38**, 1–6.

[256] Vermeulen RJ, Wolters EC, Tissingh G, Booij J, Janssen AGM, Habraken J, Sokole-Busemann E, Stoof JC, Van Royen EA (1995) Evaluation of [123I]β-CIT

binding with SPECT in controls, early and late Parkinson's disease. *Nucl Med Biol* **22**, 985–991.

[257] Antonini A, Schwarz J, Oertel WH, Pogarell O, Leenders KL (1997) Long-term changes of striatal dopamine D2 receptors in patients with Parkinson's disease: A study with positron emission tomography and [11C]raclopride. *Mov Disord* **12**, 33–38.

[258] Kim YJ, Ichise M, Ballinger JR, Vines D, Erami SS, Tatschida T, Lang AE (2002) Combination of dopamine transporter and D2 receptor SPECT in the diagnostic evaluation of PD, MSA, and PSP. *Mov Disord* **17**, 303–312.

[259] Snow BJ, Tooyama I, McGeer EG, Yamada T, Calne DB, Takahashi H, Kimura H (1993) Human positron emission tomographic [18F]Fluorodopa studies correlate with dopamine cell counts and levels. *Ann Neurol* **34**, 324–330.

[260] Li W, Lao-Kaim NP, Roussakis AA, Martín-Bastida A, Valle-Guzman N, Paul G, Loane C, Widner H, Politis M, Foltynie T, Barker RA, Piccini P (2018) 11 C-PE2I and 18 F-Dopa PET for assessing progression rate in Parkinson's: A longitudinal study. *Mov Disord* **33**, 117–127.

[261] Chou KL, Kotagal V, Bohnen NI (2016) Neuroimaging and clinical predictors of fatigue in Parkinson disease. *Park Relat Disord* **23**, 45–49.

[262] Brück A, Aalto S, Rauhala E, Bergman J, Marttila R, Rinne JO (2009) A follow-up study on 6-[18F]Fluoro-L-dopa uptake in early Parkinson's disease shows nonlinear progressionin the putamen. *Mov Disord* **24**, 1009–1015.

[263] Kapucu ÖL, Nobili F, Varrone A, Booij J, Vander Borght T, Någren K, Darcourt J, Tatsch K, Van Laere KJ (2009) EANM procedure guideline for brain perfusion SPECT using 99mTc-labelled radiopharmaceuticals, version 2. *Eur J Nucl Med*

Mol Imaging **36**, 2093–2102.

[264] Pavel DG, Henderson TA, DeBruin S (2022) The Legacy of the TTASAAN Report—Premature Conclusions and Forgotten Promises: A Review of Policy and Practice Part I. *Front Neurol* **12**, 1–34.

[265] Pavel DG, Henderson TA, DeBruin S, Cohen PF (2022) The Legacy of the TTASAAN Report – Premature Conclusions and Forgotten Promises About SPECT Neuroimaging: A Review of Policy and Practice Part II. *Front Neurol* **13**, 1–28.

[266] Kramberger MG, Štukovnik V, Čuš A, Repovš G, Tomše P, Meglič NP, Garašević Z, Jensterle J, Pirtošek Z (2010) Parkinson's disease dementia: Clinical correlates of brain SPECT perfusion and treatment. *Psychiatr Danub* **22**, 446–449.

[267] Zhu L, Zhao W, Chen J, Li G, Qu J (2022) Systematic review and meta-analysis of diagnostic test accuracy (DTA) studies: the role of cerebral perfusion imaging in prognosis evaluation of mild cognitive impairment. *Ann Palliat Med* **11**, 673–683.

[268] Feigin A, Antonini A, Fukuda M, De Notaris R, Benti R, Pezzoli G, Mentis MJ, Moeller JR, Eidelberg D (2002) Tc-99m ethylene cysteinate dimer SPECT in the differential diagnosis of parkinsonism. *Mov Disord* **17**, 1265–1270.

[269] Juni JE, Waxman AD, Devous MD, Tikofsky RS, Ichise M, Van Heertum RL, Carretta RF, Chen CC (2009) Procedure guideline for brain perfusion SPECT using99mTc radiopharmaceuticals 3.0. *J Nucl Med Technol* **37**, 191–195.

[270] Kotagal V, Spino C, Bohnen NI, Koeppe R, Albin RL (2018) Serotonin, β-amyloid, and cognition in Parkinson disease. *Ann Neurol* **83**, 994–1002.

[271] van der Zee S, Müller MLTM, Kanel P, van Laar T, Bohnen NI (2021) Cholinergic

Denervation Patterns Across Cognitive Domains in Parkinson’s Disease. *Mov Disord* **36**, 642–650.

[272] Boertien JM, Van Der Zee S, Chrysou A, Gerritsen MJJ, Jansonius NM, Spikman JM, Van Laar T, Verwey NA, Van Harten B, Portman AT, Langedijk MJH, Oomes PG, Jansen BJAM, Van Wieren T, Van Den Bogaard SJA, Van Steenbergen W, Duyff R, Van Amerongen JP, Fransen PSS, Polman SKL, Zwartbol RT, Van Kesteren ME, Braakhekke JP, Trip J, Koops L, De Langen CJ, De Jong G, Hartono JES, Ybema H, Bartels AL, Reesink FE, Postma AG, Vonk GJH, Oen JMTH, Brinkman MJ, Mondria T, Holscher RS, Van Der Meulen AAE, Rutgers AWF, Boekestein WA, Teune LK, Orsel PJL, Hoogendijk JE, Van Laar T (2020) Study protocol of the DUtch PARkinson Cohort (DUPARC): A prospective, observational study of de novo Parkinson’s disease patients for the identification and validation of biomarkers for Parkinson’s disease subtypes, progression and pathophysiology. *BMC Neurol* **20**, 1–11.

[273] Melzer TR, Stark MR, Keenan RJ, Myall DJ, MacAskill MR, Pitcher TL, Livingston L, Grenfell S, Horne KL, Young BN, Pascoe MJ, Almuqbel MM, Wang J, Marsh SH, Miller DH, Dalrymple-Alford JC, Anderson TJ (2019) Beta amyloid deposition is not associated with cognitive impairment in Parkinson’s disease. *Front Neurol* **10**, 1–9.

[274] Sabri O, Sabbagh MN, Seibyl J, Barthel H, Akatsu H, Ouchi Y, Senda K, Murayama S, Ishii K, Takao M, Beach TG, Rowe CC, Leverenz JB, Ghetti B, Ironside JW, Catafau AM, Stephens AW, Mueller A, Koglin N, Hoffmann A, Roth K, Reininger C, Schulz-Schaeffer WJ (2015) Florbetaben PET imaging to detect amyloid beta plaques in Alzheimer’s disease: Phase 3 study. *Alzheimer’s Dement* **11**, 964–974.

[275] Mashima K, Ito D, Kameyama M, Osada T, Tabuchi H, Nihei Y, Yoshizaki T, Noguchi E, Tanikawa M, Iizuka T, Date Y, Ogata Y, Nakahara T, Iwabuchi Y, Jinzaki M, Murakami K, Suzuki N (2017) Extremely low prevalence of amyloid positron emission tomography positivity in Parkinson's disease without dementia. *Eur Neurol* **77**, 231–237.

[276] Na S, Jeong H, Park J, Chung Y, Song I (2020) The Impact of Amyloid-Beta Positivity with 18F-Florbetaben PET on Neuropsychological Aspects in Parkinson ' s Disease Dementia. *Metabolites* **10**, 380.

[277] Chou KL, Dayalu P, Koeppe RA, Gilman S, Spears CC, Albin RL, Kotagal V (2022) Serotonin Transporter Imaging in Multiple System Atrophy and Parkinson's Disease. *Mov Disord* 1–7.

[278] Fazio P, Ferreira D, Svenningsson P, Halldin C, Farde L, Westman E, Varrone A (2020) High-resolution PET imaging reveals subtle impairment of the serotonin transporter in an early non-depressed Parkinson's disease cohort. *Eur J Nucl Med Mol Imaging* **47**, 2407–2416.

[279] Gomperts SN, Locascio JJ, Johnson KA, Growdon H (2016) PET Radioligands Reveal the Basis of Dementia in Parkinson's Disease and Dementia with Lewy Bodies. *Neurodegener Dis* **02129**, 118–124.

[280] Waggan I, Rissanen E, Tuisku J, Joutsa J, Helin S, Parkkola R, Rinne JO, Airas L (2022) Adenosine A2A receptor availability in patients with early- and moderate-stage Parkinson's disease. *J Neurol*.

[281] Ishibashi K, Miura Y, Wagatsuma K, Toyohara J, Ishiwata K, Ishii K (2018) Occupancy of adenosine A2A receptors by istradefylline in patients with Parkinson's disease using 11C-preladenant PET. *Neuropharmacology* **143**, 106–

112.

[282] Guedj E, Varrone A, Boellaard R, Albert NL, Barthel H, van Berckel B, Brendel M, Cecchin D, Ekmekcioglu O, Garibotto V, Lammertsma AA, Law I, Peñuelas I, Semah F, Traub-Weidinger T, van de Giessen E, Van Weehaeghe D, Morbelli S (2022) EANM procedure guidelines for brain PET imaging using [18F]FDG, version 3. *Eur J Nucl Med Mol Imaging* **49**, 632–651.

[283] Waggan I, Rissanen E, Tuisku J, Matilainen M, Helin S, Parkkola R, Rinne JO, Airas L (2021) Effect of dopaminergic medication on adenosine 2A receptor availability in patients with Parkinson's disease. *Park Relat Disord* **86**, 40–44.

[284] Minoshima S, Drzezga AE, Barthel H, Bohnen N, Djekidel M, Lewis DH, Mathis CA, Mcconathy J, Nordberg A, Sabri O, Seibyl JP (2016) SNMMI Procedure Standard / EANM Practice Guideline for Amyloid PET Imaging of the Brain 1 . 0. *J Nucl Med* **57**, 1316–1322.

[285] Ciurleo R, Di Lorenzo G, Bramanti P, Marino S (2014) Magnetic Resonance Spectroscopy: An in Vivo Molecular Imaging Biomarker for Parkinson's Disease? *Biomed Res Int* **2014**,.

[286] Payne T, Sassani M, Buckley E, Moll S, Anton A, Appleby M, Maru S, Taylor R, McNeill A, Hoggard N, Mazza C, Wilkinson ID, Jenkins T, Foltynie T, Bandmann O (2020) Ursodeoxycholic acid as a novel disease-modifying treatment for Parkinson's disease: Protocol for a two-centre, randomised, double-blind, placebo-controlled trial, the "UP" study. *BMJ Open* **10**,.

[287] Warren Olanow C, Bartus RT, Baumann TL, Factor S, Boulis N, Stacy M, Turner DA, Marks W, Larson P, Starr PA, Jankovic J, Simpson R, Watts R, Guthrie B, Poston K, Henderson JM, Stern M, Baltuch G, Goetz CG, Herzog C, Jeffrey H,

Alterman R, Lozano AM, Lang AE (2015) Gene delivery of neurturin to putamen and substantia nigra in Parkinson disease: A double-blind, randomized, controlled trial. *Ann Neurol* **78**, 248–257.

[288] Li X, Xing Y, Martin-Bastida A, Piccini P, Auer DP (2018) Patterns of grey matter loss associated with motor subscores in early Parkinson's disease. *NeuroImage Clin* **17**, 498–504.

[289] Compta Y, Pereira JB, Ríos J, Ibarretxe-Bilbao N, Junqué C, Bargalló N, Cámara A, Buongiorno M, Fernández M, Pont-Sunyer C, Martí MJ (2013) Combined dementia-risk biomarkers in Parkinson's disease: A prospective longitudinal study. *Park Relat Disord* **19**, 717–724.

[290] Agosta F, Kostic VS, Davidovic K, Kresojević N, Sarro L, Svetel M, Stanković I, Comi G, Klein C, Filippi M (2013) White matter abnormalities in Parkinson's disease patients with glucocerebrosidase gene mutations. *Mov Disord* **28**, 772–778.

[291] Burton EJ, McKeith IG, Burn DJ, Williams ED, O'Brien JT (2004) Cerebral atrophy in Parkinson's disease with and without dementia: A comparison with Alzheimer's disease, dementia with Lewy bodies and controls. *Brain* **127**, 791–800.

[292] Lanskey JH, McColgan P, Schrag AE, Acosta-Cabronero J, Rees G, Morris HR, Weil RS (2018) Can neuroimaging predict dementia in Parkinson's disease? *Brain* **141**, 2545–2560.

[293] Rolheiser TM, Fulton HG, Good KP, Fisk JD, McKelvey JR, Scherfler C, Khan NM, Leslie RA, Robertson HA (2011) Diffusion tensor imaging and olfactory identification testing in early-stage Parkinson's disease. *J Neurol* **258**, 1254–1260.

[294] Seider NA, Adeyemo B, Miller R, Newbold DJ, Hampton JM, Scheidter KM, Rutlin J, Laumann TO, Roland JL, Montez DF, Van AN, Zheng A, Marek S, Kay BP, Bretthorst GL, Schlaggar BL, Greene DJ, Wang Y, Petersen SE, Barch DM, Gordon EM, Snyder AZ, Shimony JS, Dosenbach NUF (2022) Accuracy and reliability of diffusion imaging models. *Neuroimage* **254**, 119138.

[295] Archer DB, Bricker JT, Chu WT, Burciu RG, McCracken JL, Lai S, Coombes SA, Fang R, Barmpoutis A, Corcos DM, Kurani AS, Mitchell T, Black ML, Herschel E, Simuni T, Parrish TB, Comella C, Xie T, Seppi K, Bohnen NI, Müller ML, Albin RL, Krismer F, Du G, Lewis MM, Huang X, Li H, Pasternak O, McFarland NR, Okun MS, Vaillancourt DE (2019) Development and validation of the automated imaging differentiation in parkinsonism (AID-P): a multicentre machine learning study. *Lancet Digit Heal* **1**, e222–e231.

[296] Andica C, Kamagata K, Hatano T, Saito A, Uchida W, Ogawa T, Takeshige-Amano H, Zalesky A, Wada A, Suzuki M, Hagiwara A, Irie R, Hori M, Kumamaru KK, Oyama G, Shimo Y, Umemura A, Pantelis C, Hattori N, Aoki S (2019) Free-water imaging in white and gray matter in Parkinson's disease. *Cells* **8**,.

[297] Biondetti E, Gaurav R, Yahia-Cherif L, Mangone G, Pyatigorskaya N, Valabrègue R, Ewenczyk C, Hutchison M, François C, Corvol JC, Vidailhet M, Lehéricy S (2020) Spatiotemporal changes in substantia nigra neuromelanin content in Parkinson's disease. *Brain* **143**, 2757–2770.

[298] Matsuura K, Maeda M, Tabei K ichi, Umino M, Kajikawa H, Satoh M, Kida H, Tomimoto H (2016) A longitudinal study of neuromelanin-sensitive magnetic resonance imaging in Parkinson's disease. *Neurosci Lett* **633**, 112–117.

[299] Thomas GEC, Leyland LA, Schrag AE, Lees AJ, Acosta-Cabronero J, Weil RS

(2020) Brain iron deposition is linked with cognitive severity in Parkinson’s disease. *J Neurol Neurosurg Psychiatry* **91**, 418–425.

[300] Uchida Y, Kan H, Sakurai K, Arai N, Kato D, Kawashima S, Ueki Y, Matsukawa N (2019) Voxel-based quantitative susceptibility mapping in Parkinson’s disease with mild cognitive impairment. *Mov Disord* **34**, 1164–1173.

[301] Lee JH, Lee MS (2019) Brain iron accumulation in atypical parkinsonian syndromes: In vivo MRI evidences for distinctive patterns. *Front Neurol* **10**, 1–9.

[302] Cho SJ, Bae YJ, Kim JM, Kim HJ, Baik SH, Sunwoo L, Choi BS, Jung C, Kim JH (2021) Iron-sensitive magnetic resonance imaging in Parkinson’s disease: a systematic review and meta-analysis. *J Neurol* **268**, 4721–4736.

[303] Parnetti L, Gaetani L, Eusebi P, Paciotti S, Hansson O, El-Agnaf O, Mollenhauer B, Blennow K, Calabresi P (2019) CSF and blood biomarkers for Parkinson’s disease. *Lancet Neurol* **18**, 573–586.

[304] Compta Y, Revesz T (2021) Neuropathological and biomarker findings in Parkinson’s disease and Alzheimer’s disease: From protein aggregates to synaptic dysfunction. *J Parkinsons Dis* **11**, 107–121.

[305] Mullin S, Smith L, Lee K, D’Souza G, Woodgate P, Elflein J, Hällqvist J, Toffoli M, Streeter A, Hosking J, Heywood WE, Khengar R, Campbell P, Hehir J, Cable S, Mills K, Zetterberg H, Limousin P, Libri V, Foltynie T, Schapira AHV (2020) Ambroxol for the Treatment of Patients with Parkinson Disease with and Without Glucocerebrosidase Gene Mutations: A Nonrandomized, Noncontrolled Trial. *JAMA Neurol* **77**, 427–434.

[306] Katayama T, Sawada J, Takahashi K, Yahara O (2020) Cerebrospinal fluid biomarkers in parkinson’s disease: A critical overview of the literature and meta-

analyses. *Brain Sci* **10**, 1–14.

[307] Tönges L, Buhmann C, Klebe S, Klucken J, Kwon EH, Müller T, Pedrosa DJ, Schröter N, Riederer P, Lingor P (2022) Blood-based biomarker in Parkinson's disease: potential for future applications in clinical research and practice. *J Neural Transm* **129**, 1201–1217.

[308] Morris HR (2022) Blood based biomarkers for movement disorders. *Acta Neurol Scand* **146**, 353–361.

[309] Altmann P, Leutmezer F, Zach H, Wurm R, Stattmann M, Ponleitner M, Petzold A, Zetterberg H, Berger T, Rommer P, Bsteh G (2020) Serum neurofilament light chain withstands delayed freezing and repeated thawing. *Sci Rep* **10**, 1–8.

[310] Altmann P, Ponleitner M, Rommer PS, Haslacher H, Mucher P, Leutmezer F, Petzold A, Wotawa C, Lanzenberger R, Berger T, Zetterberg H, Bsteh G (2021) Seven day pre-analytical stability of serum and plasma neurofilament light chain. *Sci Rep* **11**, 1–8.

[311] Sejbaek T, Nielsen HH, Penner N, Plavina T, Mendoza JP, Martin NA, Elkjaer ML, Ravnborg MH, Illes Z (2019) Dimethyl fumarate decreases neurofilament light chain in CSF and blood of treatment naïve relapsing MS patients. *J Neurol Neurosurg Psychiatry* **90**, 1324–1330.

[312] Ashton NJ, Janelidze S, Al Khleifat A, Leuzy A, van der Ende EL, Karikari TK, Benedet AL, Pascoal TA, Lleó A, Parnetti L, Galimberti D, Bonanni L, Pilotto A, Padovani A, Lycke J, Novakova L, Axelsson M, Velayudhan L, Rabinovici GD, Miller B, Pariante C, Nikkheslat N, Resnick SM, Thambisetty M, Schöll M, Fernández-Eulate G, Gil-Bea FJ, López de Munain A, Al-Chalabi A, Rosa-Neto P, Strydom A, Svenningsson P, Stomrud E, Santillo A, Aarsland D, van Swieten

JC, Palmqvist S, Zetterberg H, Blennow K, Hye A, Hansson O (2021) A multicentre validation study of the diagnostic value of plasma neurofilament light. *Nat Commun* **12**,.

[313] Mollenhauer B, Dakna M, Kruse N, Galasko D, Foroud T, Zetterberg H, Schade S, Gera RG, Wang W, Gao F, Frasier M, Chahine LM, Coffey CS, Singleton AB, Simuni T, Weintraub D, Seibyl J, Toga AW, Tanner CM, Kieburtz K, Marek K, Siderowf A, Cedarbaum JM, Hutten SJ, Trenkwalder C, Graham D (2020) Validation of Serum Neurofilament Light Chain as a Biomarker of Parkinson's Disease Progression. *Mov Disord* **35**, 1999–2008.

[314] Oosterveld LP, Verberk IMW, Majbour NK, El-Agnaf OM, Weinstein HC, Berendse HW, Teunissen CE, van de Berg WDJ (2020) CSF or serum neurofilament light added to α-Synuclein panel discriminates Parkinson's from controls. *Mov Disord* **35**, 288–295.

[315] Huang Y, Huang C, Zhang Q, Shen T, Sun J (2022) Serum NFL discriminates Parkinson disease from essential tremor and reflect motor and cognition severity. *BMC Neurol* **22**, 1–8.

[316] Ygland Rödström E, Mattsson-Carlgren N, Janelidze S, Hansson O, Puschmann A (2021) Serum Neurofilament Light Chain as a Marker of Progression in Parkinson's Disease: Long-Term Observation and Implications of Clinical Subtypes. *J Parkinsons Dis* **12**, 571–584.

[317] Ng ASL, Tan YJ, Yong ACW, Saffari SE, Lu Z, Ng EY, Ng SYE, Chia NSY, Choi X, Heng D, Neo S, Xu Z, Keong NCH, Tay KY, Au WL, Tan LCS, Tan EK (2020) Utility of plasma Neurofilament light as a diagnostic and prognostic biomarker of the postural instability gait disorder motor subtype in early Parkinson's disease.

Mol Neurodegener **15**, 1–8.

[318] Sampedro F, Pérez-González R, Martínez-Horta S, Marín-Lahoz J, Pagonabarraga J, Kulisevsky J (2020) Serum neurofilament light chain levels reflect cortical neurodegeneration in de novo Parkinson's disease. *Park Relat Disord* **74**, 43–49.

[319] Gaetani L, Blennow K, Calabresi P, Di Filippo M, Parnetti L, Zetterberg H (2019) Neurofilament light chain as a biomarker in neurological disorders. *J Neurol Neurosurg Psychiatry* 870–881.

[320] Karikari TK, Pascoal TA, Ashton NJ, Janelidze S, Benedet AL, Rodriguez JL, Chamoun M, Savard M, Kang MS, Therriault J, Schöll M, Massarweh G, Soucy JP, Höglund K, Brinkmalm G, Mattsson N, Palmqvist S, Gauthier S, Stomrud E, Zetterberg H, Hansson O, Rosa-Neto P, Blennow K (2020) Blood phosphorylated tau 181 as a biomarker for Alzheimer's disease: a diagnostic performance and prediction modelling study using data from four prospective cohorts. *Lancet Neurol* **19**, 422–433.

[321] Gonzalez MC, Ashton NJ, Gomes BF, Tovar-Rios DA, Blanc F, Karikari TK, Mollenhauer B, Pilotto A, Lemstra A, Paquet C, Abdelnour C, Kramberger MG, Bonanni L, Vandenberghe R, Hye A, Blennow K, Zetterberg H, Aarsland D (2022) Association of Plasma p-tau181 and p-tau231 Concentrations with Cognitive Decline in Patients with Probable Dementia with Lewy Bodies. *JAMA Neurol* **79**, 32–37.

[322] Ding XL, Tuo QZ, Lei P (2021) An Introduction to Ultrasensitive Assays for Plasma Tau Detection. *J Alzheimer's Dis* **80**, 1353–1362.

[323] Lin CH, Yang SY, Horng HE, Yang CC, Chieh JJ, Chen HH, Liu BH, Chiu MJ (2018) Plasma biomarkers differentiate Parkinson's disease from atypical

parkinsonism syndromes. *Front Aging Neurosci* **10**, 1–9.

[324] Chen NC, Chen HL, Li SH, Chang YH, Chen MH, Tsai NW, Yu CC, Yang SY, Lu CH, Lin WC (2020) Plasma Levels of α-Synuclein, Aβ-40 and T-tau as Biomarkers to Predict Cognitive Impairment in Parkinson's Disease. *Front Aging Neurosci* **12**, 1–8.

[325] Ren J, Pan C, Wang Y, Xue C, Lin H, Xu J, Wang H, Zhang W, Xu P, Chen Y, Liu W (2022) Plasma α-synuclein and phosphorylated tau 181 as a diagnostic biomarker panel for de novo Parkinson's Disease . *J Neurochem* 0–2.

[326] Youssef P, Kim WS, Halliday GM, Lewis SJG, Dzamko N (2021) Comparison of Different Platform Immunoassays for the Measurement of Plasma Alpha-Synuclein in Parkinson's Disease Patients. *J Parkinsons Dis* **11**, 1761–1772.

[327] Barbour R, Kling K, Anderson JP, Banducci K, Cole T, Diep L, Fox M, Goldstein JM, Soriano F, Seubert P, Chilcote TJ (2008) Red blood cells are the major source of alpha-synuclein in blood. *Neurodegener Dis* **5**, 55–59.

[328] Zubelzu M, Morera-Herreras T, Irastorza G, Gómez-Esteban JC, Murueta-Goyena A (2022) Plasma and serum alpha-synuclein as a biomarker in Parkinson's disease: A meta-analysis. *Park Relat Disord* **99**, 107–115.

[329] Williams SM, Schulz P, Sierks MR (2016) Oligomeric α-synuclein and β-amyloid variants as potential biomarkers for Parkinson's and Alzheimer's diseases. *Eur J Neurosci* **43**, 3–16.

[330] Li XY, Li W, Li X, Li XR, Sun L, Yang W, Cai Y, Chen Z, Wu J, Wang C, Yu S (2021) Alterations of Erythrocytic Phosphorylated Alpha-Synuclein in Different Subtypes and Stages of Parkinson's Disease. *Front Aging Neurosci* **13**, 1–12.

[331] Niu M, Li Y, Li G, Zhou L, Luo N, Yao M, Kang W, Liu J (2020) A longitudinal

study on α-synuclein in plasma neuronal exosomes as a biomarker for Parkinson's disease development and progression. *Eur J Neurol* **27**, 967–974.

[332] Kluge A, Bunk J, Schaeffer E, Drobny A, Xiang W, Knacke H, Bub S, Lückstädt W, Arnold P, Lucius R, Berg D, Zunke F (2022) Detection of neuron-derived pathological α -synuclein in blood. 3058–3071.

[333] Foulds PG, Diggle P, Mitchell JD, Parker A, Hasegawa M, Masuda-Suzukake M, Mann DMA, Allsop D (2013) A longitudinal study on a-synuclein in blood plasma as a biomarker for Parkinson's disease. *Sci Rep* **3**, 1–6.

[334] Malec-Litwinowicz M, Plewka A, Plewka D, Bogunia E, Morek M, Szczudlik A, Szubiga M, Rudzińska-Bar M (2018) The relation between plasma α-synuclein level and clinical symptoms or signs of Parkinson's disease. *Neurol Neurochir Pol* **52**, 243–251.

[335] Chung CC, Chan L, Chen JH, Hung YC, Hong CT (2021) Plasma extracellular vesicle α-synuclein level in patients with parkinson's disease. *Biomolecules* **11**, 1–10.

[336] Bäckström D, Granåsen G, Mo SJ, Riklund K, Trupp M, Zetterberg H, Blennow K, Forsgren L, Domellöf ME (2022) Prediction and early biomarkers of cognitive decline in Parkinson disease and atypical parkinsonism : a population-based study. *Brain Commun* **15**, fcac040.

[337] Lerche S, Wurster I, Röben B, Zimmermann M, Machetanz G, Wiethoff S, Dehnert M, Rietschel L, Riebenbauer B, Deuschle C, Stransky E, Lieplt-Scarfone I, Gasser T, Brockmann K (2020) CSF NFL in a Longitudinally Assessed PD Cohort: Age Effects and Cognitive Trajectories. *Mov Disord* **35**, 1138–1144.

[338] Kwon EH, Tennagels S, Gold R, Gerwert K, Beyer L, Tönges L (2022) Update on

CSF Biomarkers in Parkinson's Disease. *Biomolecules* **12**, 329.

[339] Bäckström D, Linder J, Jakobson Mo S, Riklund K, Zetterberg H, Blennow K, Forsgren L, Lenfeldt N (2020) NfL as a biomarker for neurodegeneration and survival in Parkinson disease. *Neurology* **95**, e827–e838.

[340] Bäckström DC, Domellöf ME, Linder J, Olsson B, Öhrfelt A, Trupp M, Zetterberg H, Blennow K, Forsgren L (2015) Cerebrospinal fluid patterns and the risk of future dementia in early, incident Parkinson disease. *JAMA Neurol* **72**, 1175–1182.

[341] Lifke V, Kollmorgen G, Manuilova E, Oelschlaegel T, Hillringhaus L, Widmann M, von Arnim CAF, Otto M, Christenson RH, Powers JL, Shaw LM, Hansson O, Doecke JD, Li QX, Teunissen C, Tumani H, Blennow K (2019) Elecsys® Total-Tau and Phospho-Tau (181P) CSF assays: Analytical performance of the novel, fully automated immunoassays for quantification of tau proteins in human cerebrospinal fluid. *Clin Biochem* **72**, 30–38.

[342] Liu C, Cholerton B, Shi M, Ginghina C, Cain KC, Auinger P, Zhang J (2015) CSF tau and tau/Aβ42 predict cognitive decline in Parkinson's disease. *Park Relat Disord* **21**, 271–276.

[343] Mollenhauer B, Bowman FDB, Drake D, Duong J, Blennow K, El-Agnaf O, Shaw LM, Masucci J, Taylor P, Umek RM, Dunty JM, Smith CL, Stoops E, Vanderstichele H, Schmid AW, Moniatte M, Zhang J, Kruse N, Lashuel HA, Teunissen C, Schubert T, Dave KD, Hutten SJ, Zetterberg H (2019) Antibody-based methods for the measurement of α-synuclein concentration in human cerebrospinal fluid – method comparison and round robin study. *J Neurochem* **149**, 126–138.

[344] Constantinides VC, Majbour NK, Paraskevas GP, Abdi I, Safieh-Garabedian B,

Stefanis L, El-Agnaf OM, Kapaki E (2021) Cerebrospinal fluid α-synuclein species in cognitive and movements disorders. *Brain Sci* **11**, 1–12.

[345] Majbour NK, Vaikath NN, Eusebi P, Chiasserini D, Ardah M, Varghese S, Haque ME, Tokuda T, Auinger P, Calabresi P, Parnetti L, El-Agnaf OMA (2016) Longitudinal changes in CSF alpha-synuclein species reflect Parkinson's disease progression. *Mov Disord* **31**, 1535–1542.

[346] Bargar C, Wang W, Gunzler SA, LeFevre A, Wang Z, Lerner AJ, Singh N, Tatsuoka C, Appleby B, Zhu X, Xu R, Haroutunian V, Zou WQ, Ma J, Chen SG (2021) Streamlined alpha-synuclein RT-QuIC assay for various biospecimens in Parkinson's disease and dementia with Lewy bodies. *Acta Neuropathol Commun* **9**, 1–13.

[347] Kang UJ, Boehme AK, Fairfoul G, Shahnawaz M, Ma TC, Hutten SJ, Green A, Soto C (2019) Comparative study of cerebrospinal fluid α-synuclein seeding aggregation assays for diagnosis of Parkinson's disease. *Mov Disord* **34**, 536–544.

[348] Poggiolini I, Gupta V, Lawton M, Lee S, El-Turabi A, Querejeta-Coma A, Trenkwalder C, Sixel-Döring F, Foubert-Samier A, Pavy-Le Traon A, Plazzi G, Biscarini F, Montplaisir J, Gagnon J-F, Postuma RB, Antelmi E, Meissner WG, Mollenhauer B, Ben-Shlomo Y, Hu MT, Parkkinen L (2021) Diagnostic value of cerebrospinal fluid alpha-synuclein seed quantification in synucleinopathies. *Brain*.

[349] Orrù CD, Ma TC, Hughson AG, Groveman BR, Srivastava A, Galasko D, Angers R, Downey P, Crawford K, Hutten SJ, Kang UJ, Caughey B (2021) A rapid α-synuclein seed assay of Parkinson's disease CSF panel shows high diagnostic accuracy. *Ann Clin Transl Neurol* **8**, 374–384.

[350] Iranzo A, Fairfoul G, Ayudhaya ACN, Serradell M, Gelpi E, Vilaseca I, Sanchez-Valle R, Gaig C, Santamaria J, Tolosa E, Riha RL, Green AJE (2021) Detection of α-synuclein in CSF by RT-QuIC in patients with isolated rapid-eye-movement sleep behaviour disorder: a longitudinal observational study. *Lancet Neurol* **20**, 203–212.

[351] Shahnawaz M, Tokuda T, Waraga M, Mendez N, Ishii R, Trenkwalder C, Mollenhauer B, Soto C (2017) Development of a biochemical diagnosis of Parkinson disease by detection of α-synuclein misfolded aggregates in cerebrospinal fluid. *JAMA Neurol* **74**, 163–172.

[352] V-PLEX Aβ Peptide Panel 1 (6E10) Kit, Last updated 2022, Accessed on 2022.

[353] NEUROLOGY 3-PLEX A (TAU, Aβ42, Aβ40), Last updated 2022, Accessed on 2022.

[354] Boulo S, Kuhlmann J, Andreasson U, Brix B, Venkataraman I, Herbst V, Rutz S, Manuilova E, Vandijck M, Dekeyser F, Bjerke M, Pannee J, Charoud-Got J, Auclair G, Mazoua S, Pinski G, Trapmann S, Schimmel H, Emons H, Quaglia M, Portelius E, Korecka M, Shaw LM, Lame M, Chambers E, Vanderstichele H, Stoops E, Leinenbach A, Bittner T, Jenkins RG, Kostanjevecki V, Lewczuk P, Gobom J, Zetterberg H, Zegers I, Blennow K (2020) First amyloid β1-42 certified reference material for re-calibrating commercial immunoassays. *Alzheimer's Dement* **16**, 1493–1503.

[355] Janelidze S, Stomrud E, Brix B, Hansson O (2019) Towards a unified protocol for handling of CSF before β-amyloid measurements. *Alzheimer's Res Ther* **11**, 1–12.

[356] Lewczuk P, Riederer P, O'Bryant SE, Verbeek MM, Dubois B, Visser PJ, Jellinger KA, Engelborghs S, Ramirez A, Parnetti L, Jack CR, Teunissen CE, Hampel H,

Lleó A, Jessen F, Glodzik L, de Leon MJ, Fagan AM, Molinuevo JL, Jansen WJ, Winblad B, Shaw LM, Andreasson U, Otto M, Mollenhauer B, Wiltfang J, Turner MR, Zerr I, Handels R, Thompson AG, Johansson G, Ermann N, Trojanowski JQ, Karaca I, Wagner H, Oeckl P, van Waalwijk van Doorn L, Bjerke M, Kapogiannis D, Kuiperij HB, Farotti L, Li Y, Gordon BA, Epelbaum S, Vos SJB, Klijn CJM, Van Nostrand WE, Minguillon C, Schmitz M, Gallo C, Lopez Mato A, Thibaut F, Lista S, Alcolea D, Zetterberg H, Blennow K, Kornhuber J (2018) Cerebrospinal fluid and blood biomarkers for neurodegenerative dementias: An update of the Consensus of the Task Force on Biological Markers in Psychiatry of the World Federation of Societies of Biological Psychiatry. *World J Biol Psychiatry* **19**, 244–328.

[357] Lim EW, Aarsland D, Ffytche D, Taddei RN, van Wamelen DJ, Wan YM, Tan EK, Ray Chaudhuri K (2019) Amyloid-β and Parkinson's disease. *J Neurol* **266**, 2605–2619.

[358] Alves G, Brønnick K, Aarsland D, Blennow K, Zetterberg H, Ballard C, Kurz MW, Andreasson U, Tysnes OB, Larsen JP, Mulugeta E (2010) CSF amyloid-β and tau proteins, and cognitive performance, in early and untreated Parkinson's Disease: The Norwegian ParkWest study. *J Neurol Neurosurg Psychiatry* **81**, 1080–1086.

[359] Hall S, Surova Y, Öhrfelt A, Zetterberg H, Lindqvist D, Hansson O (2015) CSF biomarkers and clinical progression of Parkinson disease. *Neurology* **84**, 57–63.

[360] Pawlik P, Błochowiak K (2021) The role of salivary biomarkers in the early diagnosis of Alzheimer's disease and Parkinson's disease. *Diagnostics* **11**,.

[361] Kharel S, Ojha R, Bist A, Joshi SP, Rauniyar R, Yadav JK (2022) Salivary alpha-synuclein as a potential fluid biomarker in Parkinson's disease: A systematic

review and meta-analysis. *Aging Med* **5**, 53–62.

[362] Vivacqua G, Mason M, De Bartolo MI, Węgrzynowicz M, Calò L, Belvisi D, Suppa A, Fabbrini G, Berardelli A, Spillantini MG (2022) Salivary α-Synuclein RT-QuIC Correlates with Disease Severity in de novo Parkinson's Disease. *Mov Disord* 1–4.

Supplementary Table 1. Summary of MDS and NINDS-CDE recommendations and our selected OM

Category	Recommended in<br>MDS review	Included in<br>NINDS-CDE v2.0	Included in this longlist	Comments
Scales for specific motor symptoms – Gait, balance and falls [33]	Scales without extra accessories:<br>PIGD Score<br>Scales that require extra accessories:<br>Berg Balance Scale<br>Mini-BESTest®<br>Dynamic Gait Index<br>Functional Gait Assessment®<br>Questionnaires:<br>ABC Scale<br>FES<br>FOG Questionnaire<br>SAFFE-m	No specific instruments included in the NINDS-CDE v2.0	Motor part of the MDS-UPDRS (as motor measure)<br>ProFaNE definition of a fall<br>TUG (as quantitative motor measure)<br>Mini-BESTest®<br>Berg Balance Scale<br>FES-I<br>ABC Scale	Final list based on a recent systematic review of tools to measure balance and predict risk of falls in PD, and on expert recommendation

	Tests:<br>6-minute walk test<br>10-m walk test<br>TUG<br>Functional Reach			
Scales for specific motor symptoms – Speech and swallowing [41]	No MDS review on speech scales in PD.<br>Swallowing – None recommended:<br>SWAL-QOL (Suggested)<br>SDQ (Suggested)	No specific instruments for speech included in the NINDS-CDE v2.0<br>Swallowing:<br>Supplemental:<br>SWAL-QOL<br>ROMP<br>SDQ<br>SCAS-PD	SWAL-QOL<br>SDQ<br>ROMP	SDQ and ROMP are PD-specific, and ROMP was developed after the 2009 MDS review
Diaries and other fluctuation questionnaires [57]	WOQ19<br>WOQ9<br>Motor Fluctuation Diaries (most common: PD Diary by Hauser)	Supplemental – Highly Recommended:<br>WOQ<br>Hauser Motor Fluctuation Diary<br>CAPSIT-PD	WOQ9 and WOQ19<br>Hauser PD Diary<br>CAPSIT-PD Diaries	All OM included both in the MDS review and in the NINDS-CDE

	CAPSIT-PD Diaries			
Scales for specific non-motor symptoms: Apathy [109]	AS<br>UPDRS item 4	Supplemental – Highly Recommended:<br>AS<br>DAS<br>DAS – Informant/Carer-Rated Version<br>LARS	AS<br>AES<br>LARS	AS and LARS preferred, but AES now also meets criteria for "Recommended".
Scales for specific non-motor symptoms: Depression [96]	No definite recommendations, but the following are deemed useful for PD with dementia:<br>MADRS<br>GDS<br>CSDD<br>And the following are identified as validated and widely used in PD patients:	Supplemental – Highly Recommended:<br>GDS-15<br>C-SSRS<br>C-SSRS Screener Version	GDS-15<br>PHQ-9<br>C-SSRS*	GDS-15 is an expert-based recommendation. A 2012 comparison of 9 depression rating scales in PD concluded that the GDS-30 was the most complete OM,

	BDI<br>HAM-D<br>Observer-rated scales (MADRS, HAM-D) should be preferred to self-rated scales since they have better psychometric properties			and for brevity we have included the GDS-15.<br>PHQ-9 and C-SSRS were included as expert recommendation.
Scales for specific non-motor symptoms: Fatigue [83]	FSS<br>FACIT-F<br>PFS	Supplemental:<br>FSS<br>PFS-16	FSS	
Scales for specific non-motor symptoms: Pain [86]	KPPS	Supplemental:<br>KPPS<br>NRS-Box 21 Scale<br>IASP Definitions for Pain	KPPS	Only pain measure included both in the MDS review and in the NINDS-CDE
Scales for specific non-motor	None alone, but the authors recommend using a scale to	Supplemental – Highly Recommended:<br>SAPS-PD	SAPS-PD<br>eSAPS-PD	SAPS-PD included both in the MDS

symptoms:<br>Psychosis [117]	measure clinical response and change over time with another one which catalogues specific features.<br><br>For clinical trials on PD psychosis assessing new treatments, the following are recommended primary outcome scales:<br>NPI (for the cognitively impaired PD population or when a caregiver is required)<br>SAPS<br>PANSS<br>BPRS (for the cognitively intact PD population or when the patient is the sole informant)	eSAPS-PD	review and in the NINDS-CDE

	The CGIS is suggested as a<br>secondary<br>outcome scale to measure change<br>and response to treatment over<br>time.			
Scales for specific non-motor symptoms: Sleep [89](MDS review 2010)	PDSS<br>PSQI<br>SCOPA-Sleep<br>ESS	Supplemental – Highly Recommended:<br>ESS<br>MSLT and Guidelines<br>PDSS-2<br>PSG and Guidelines<br>SCOPA-Sleep<br>AASM: ICSD Criteria	PDSS-2 (for sleep)<br>ESS (for sleepiness)	ESS included both in the MDS review and in the NINDS-CDE. PDSS-2 developed after the MDS review but already validated.
Scales for specific non-motor symptoms:	2009 – Recommended:<br>SCOPA-AUT<br>NMSQ	Supplemental – Highly Recommended:<br>COMPASS-31<br><br>Supplemental:	SCOPA-AUT	SCOPA-AUT included in both MDS reviews and in the NINDS-CDE.

Dysautonomia [41,121]	2011 – Recommended (with some limitations):<br>SCOPA-AUT<br>COMPASS	SCOPA-AUT		
Disability [162]	FSQ<br>Lawton-Brody ADL<br>Nottingham Extended ADL<br>PROMIS and Neuro-QoL<br>S&E ADL<br>SPDDS<br>SPES-SCOPA<br>UPDRS-ADL (part II)<br>MDS-UPDRS M-EDL (part II)	QoL/PRO:<br>EQ-5D (Supplemental – Highly Recommended as a generic QoL measure)<br>PDQL (Supplemental)<br>PDQUALIF (Supplemental)<br>PDQ-39 (Supplemental – Highly Recommended as a PD-specific QoL measure)<br>PIMS (Supplemental)<br>PROMIS v1.2 – Global Health (Supplemental as a global HR-QoL measure)	S&E ADL (also included in NINDS-CDE, in “Motor function” (Supplemental – Highly Recommended))<br>MDS-UPDRS M-EDL (part II) (MDS-UPDRS also included in NINDS-CDE, in “Motor function” (Core))<br>FSQ<br>PROMIS/Neuro-QoL	MDS-UPDRS (either full or only part II) and S&E ADL were the only recommended scales in a 2022 systematic review

HR-QoL (generic) [181]	EQ-5D<br>NHP<br>SF-36<br>SIP	PROMIS-29 Profile (Supplemental as a global HR-QoL measure)<br>Neuro-QoL (Supplemental – Highly Recommended)<br>SCOPA-PS (Supplemental)<br>SEIQoL (Supplemental)<br>SEIQoL-DW (Supplemental)<br>SF-36 (Supplemental - Highly Recommended as a generic QoL measure)<br>WHOQOL-BREF (Supplemental)	EQ-5D<br>SF-36<br>HUI	HUI is also a Health-Economics measure, "Recommended" in the 2013 MDS review of measures for health-economic studies
HR-QoL (PD-specific) [181]	PDQ-39<br>PDQ-8<br>PDQL<br>PIMS<br>SCOPA-PS		PDQ-39<br>PDQ-8	Both PDQs are included in both classifications (MDS and NINDS-CDE).

Health economics – Resource use [182]	Cost-utility analysis<br><br>Recommended (with some limitations):<br>EQ-5D<br><br>Recommended (but requires validation in PD):<br>SF-6D<br>15D<br>HUI-III<br>HUI-II	N/A	CSRI<br>EHR	Based on expert recommendation.
Cognitive scales [124]	MoCA<br>DRS-2<br>PD-CRS	Core:<br>MoCA<br>Supplemental:	MoCA<br>ACE-III<br>ADAS-cog	Based on expert recommendation.

	ACE-III<br>ADAS-cog<br>CAMCOG-R<br>CANTAB<br>CDR System<br>MDRS<br>MMSE<br>PD-CRS<br>SCOPA-COG<br>PD-CFRS	MMSE<br>MMP<br>PD-CRS<br>SCOPA-COG<br>DRS-2

*Administration of the C-SSRS is recommended if screening question on the PHQ-9 is > 0.

Note: Only OM categories for which MDS critique and review papers existed have been included in this table (e.g., wet biomarkers have not been included). Tremor and dyskinesia are included within the “motor” section of this publication, and the recommendations in that case were mostly derived from expert consensus. For a detailed list of included outcomes in the NINDS-CDE version 2.0, please visit https://www.commondataelements.ninds.nih.gov/Parkinson%27s%20Disease#pane-158.

AASM: American Academy of Sleep Medicine; ABC Scale: Activities-Specific Balance Confidence Scale; ACE-III: Addenbrooke’s Cognitive Examination; ADAS-Cog: Alzheimer's Disease Assessment Scale-Cognitive Subscale; ADL: Activities of Daily Living; AES:

Apathy Evaluation Scale; AS: Apathy Scale; BDI: Beck Depression Inventory; BPRS: Brief Psychiatric Rating Scale; C-SSRS: Columbia Suicide Severity Rating Scale; CAMCOG: Cambridge Cognition Examination; CANTAB: Cambridge Neuropsychological Test Automated Battery; CAPSIT-PD: Core assessment program for surgical interventional therapies in Parkinson's disease; CGIS: Clinical Global Impression Scale; COMPASS(-31): (31-item) Composite Autonomic Symptom Scale; CSDD: Cornell Scale for Depression in Dementia; CSRI: Client Service Receipt Inventory; DAS: Dimensional Apathy Scale; DRS-2: Mattis Dementia Rating Scale Second Edition; EHR: Electronic health records; eSAPS-PD: Scale for the Assessment of Positive Symptoms in Parkinson's Disease, enhanced version; ESS: Epworth Sleepiness Scale; FACIT-F: Functional Assessment of Chronic Illness Therapy - Fatigue; FES(-I): Falls Efficacy Scale (-International); FOG: Freezing Of Gait; FSQ: Functional Status Questionnaire; FSS: Fatigue Severity Scale; GDS(-15/-30): (15-/30-item) Geriatric Depression Scale; HAM-D: Hamilton Depression Scale; HUI(-II/-III): Health Utility Index (-Mark2/-Mark 3); IASP: International Association for the Study of Pain; ICSD: International Classification of Sleep Disorders; KPPS: King's Parkinson's Disease Pain Scale; LARS: Lille Apathy Rating Scale; M-EDL: Motor Experiences of Daily Living; MADRS: Montgomery-Åsberg Depression Rating Scale; MDRS: Mattis Dementia Rating Scale; MDS: Movement Disorders Society; MDS-UPDRS: Movement Disorders Society-sponsored revision of the Unified Parkinson's Disease Rating Scale; MDT-PD: Munich Dysphagia Test – Parkinson's Disease; Mini-BESTest: Mini-Balance Evaluation Systems Test; MMP: Mini-Mental Parkinson; MMSE: Mini-Mental State Examination; MoCA: Montreal Cognitive Assessment; MSLT: Multiple Sleep Latency Test; NHP: Nottingham Health Profile; NINDS-CDE: National Institute of Neurological Disorders and Stroke Common Data Elements; NMSQ: Non-motor Symptoms Questionnaire; NPI: Neuropsychiatric

Inventory; NRS: Numeric Rating Scale; OM: outcome measure(s); PANSS: Positive and Negative Syndrome Scale; PD-CFRS: Parkinson's Disease - Cognitive Functional Rating Scale; PD-CRS: Parkinson's Disease-Cognitive Rating Scale; PDQ-8: 8-item version of the Parkinson's Disease Questionnaire; PDQ-39: 39-tem version of the Parkinson's Disease Questionnaire; PDQL: Parkinson's Disease Quality of Life Questionnaire; PDQUALIF: Parkinson's Disease Quality of Life Scale; PDSS(-2): Parkinson's Disease Sleep Scale(-2); PFS(-16): (16-item) Parkinson's Disease Fatigue Scale; PHQ-9: 9-item Patient Health Questionnaire; PIGD: Postural Instability and Gait Disorder; PIMS: Parkinson's Impact Scale; PRO: Patient-wReported Outcomes; ProFaNE: Prevention of Falls Network Earth; PROMIS/Neuro-QoL: Patient-Reported Outcomes Measurement Information System/Quality of Life in Neurological Disorders; PSG: Polysomnography; PSQI: Pittsburgh Sleep Quality Index; QoL: Quality of Life; ROMP: Radboud Oral Motor Inventory for Parkinson's Disease; S&E ADL SCALE: Schwab and England Activities of Daily Living Scale; SAFFE-m: Modified Survey of Activities and Fear of Falling in the Elderly; SAPS-PD: Scale for the Assessment of Positive Symptoms in Parkinson's Disease; SCAS-PD: Swallowing Clinical Assessment Score in Parkinson's Disease; SCOPA-AUT: Scales for Outcomes in Parkinson's disease-AUTonomic symptoms; SCOPA-COG: Scales for Outcomes in Parkinson's disease-COGnitive symptoms; SCOPA-PS: SCales for Outcomes in PArkinson's disease- Psychosocial Functioning; SCOPA-Sleep: Scales for Outcomes in Parkinson's disease-Sleep; SDQ: Swallowing Disturbance Questionnaire; SEIQoL(-DW): Schedule for the Evaluation of Individual Quality of Life (-Direct Weighting); SF-36: 36-Item Short Form Survey; SIP: Sickness Impact Profile; SPDDS: Self-Assessment Parkinson's Disease Disability Scale; SPES/SCOPA: Short Parkinson's Evaluation Scale (SPES)/SCales for Outcomes in Parkinson's disease; SWAL-QOL: Generic Scale for Dysphagia-Related Outcomes (Quality of Life); TUG:

Timed Up and Go; UPDRS: Unified Parkinson's Disease Rating Scale; WHOQOL-BREF: WHO Quality of Life Assessment Short Version; WOQ-9: 9-item Wearing Off Questionnaire; WOQ-19: 19-item Wearing Off Questionnaire.

Supplementary Table 2. Recommended Cognitive Core Data Elements' (CDEs) scorings (reproduced with permission from Table 2 of the NINDS-CDE v2.0 Parkinson's Disease Cognitive Subgroup Summary document) [156].

Instrument Type/Name	Rating Scale Usage				
	Screening Instrument[a]	Severity[b]	Longitudinal[c]	Diagnostic Instrument[d]	Administration Time (minutes)
Paper-and-Pencil					
ADAS-Cog[◊]	2	2	N/A	2	30
ACE-III	1.5	2	3	2	15-20
CAMCOG-R[†]	2	2	2	2	25-30
Mattis DRS-2[□]	1	1	1	1	30-50
MMSE[□]	2.5	2	2	2.5	10-12
MoCA[▲]	1	1	1	1	10-15
PD-CRS[◊]	1.5	1.5	1.5	1.5	17-26
SCOPA-COG[◊]	2	1.5	3	3	10-15

[a] Screening Instrument - For initial identification of possible disorder

[b] Rating Scale - For measurement of disorder severity

[c] Longitudinal - Sensitivity to change over time

[d] Diagnostic Criteria and Instrument – Categorization of patients into those with and without a disorder

[†] Instrument available from author

[◊] Instrument available in public domain

▲ Free to investigators

□ Copyrighted instrument

Please note: The disorder refers to cognitive impairment in Parkinson's disease including mild cognitive impairment and dementia. Each of the above scales are being given a score of 1, 2, 3 for suitability (1= highest or best, 3= lowest or worst). A scale may be suitable for mild cognitive impairment and/or dementia.

Other scales were reviewed by the subgroup but were not recommended for PD v2.0. They were not classified as Exploratory since they do not currently fill gaps in PD research in the context of cognition.

ACE-III: Addenbrooke's Cognitive Examination; ADAS-Cog: Alzheimer's Disease Assessment Scale-Cognitive Subscale; CAMCOG-R: Cambridge Cognitive Assessment-Revised; CDEs: Core Data Elements; Mattis DRS-2: Mattis Dementia Rating Scale Second Edition; MMSE: Mini-Mental State Examination; MoCA: Montreal Cognitive Assessment; NINDS-CDE v2.0: National Institute of Neurological Disorders and Stroke Common Data Elements, version 2.0; PD: Parkinson's Disease; PD-CRS: Parkinson's Disease-Cognitive Rating Scale; SCOPA-COG: SCales for Outcomes in PArkinson's disease- COGnitive symptoms.
