# Supplementary material for: Outcome Measures for Disease-Modifying Trials in Parkinson’s Disease: Consensus Paper by the EJS ACT-PD Multi-Arm Multi-Stage Trial Initiative
Source: J Parkinsons Dis. 2023 Sep 8;13(6):1011–33. doi: 10.3233/JPD-230051 (PMC10578294; doi:10.3233/JPD-230051)
Supplement: Supplementary Material 2 [file jpd-13-jpd230051-s002.pdf]

Supplementary Material

Outcome Measures for Disease-Modifying Trials in Parkinson's Disease: Consensus paper by the EJS ACT-PD Multi-Arm Multi-Stage Trial Initiative

Supplementary Material 2. EJS ACT-PD Consortium

Member	Role	Organisation	Email
Thomas Foltynie	Co-Lead	University College London, London, UK	t.foltynie@ucl.ac.uk
Camille B Carroll	Co-Lead	University of Plymouth, Plymouth, UK	camille.carroll@plymouth.ac.uk
Roger Barker	Chair	University of Cambridge, Cambridge, UK	rab46@cam.ac.uk
James Carpenter	Chair	MRC Clinical Trials Unit at UCL, London, UK	James.Carpenter@lshtm.ac.uk
Yoav Ben Shlomo	Member	University of Bristol, Bristol, UK	Y.Ben-Shlomo@bristol.ac.uk
Mark Edwards	Member	St George's University of London, London, UK	medwards@sgul.ac.uk
Alan Whone	Member	University of Bristol, Bristol, UK	Alan.Whone@bristol.ac.uk
Carl Counsell	Member	University of Bristol, Bristol, UK	carl.counsell@abdn.ac.uk
Caroline S Clarke	Member	University College London, London, UK	caroline.clarke@ucl.ac.uk
Matthew Burnell	Member	MRC Clinical Trials Unit at UCL, London, UK	m.burnell@ucl.ac.uk
Dorothy Salathiel	Patient and Public Involvement and Engagement representative (PPI)	Expert by Experience	dorothy.sm99@gmail.com
Sue Whipps	PPI	Expert by Experience	sue.whipps@btinternet.com
Anna Jewell	PPI	Expert by Experience	anna.lgjewell@gmail.com
Priti Gros	Member	University of Toronto, Toronto, Canada	Priti.Gros@uhn.ca
Tom Barber	Early Career Reaearcher (ECR)	University of Oxford, Oxford, UK	Tom.Barber@ouh.nhs.uk
Anette Schrag	Chair	University College London, London, UK	a.schrag@ucl.ac.uk
Rimona S Weil	Deputy Chair	University College London, London, UK	r.weil@ucl.ac.uk
Caroline H Williams-Gray	Member	University of Cambridge, UK	chm27@cam.ac.uk

Michele T Hu	Member	University of Oxford, Oxford, UK	michele.hu@ndcn.ox.ac.uk
Lynn Rochester	Member	Newcastle University, Newcastle, UK	lynn.rochester@newcastle.ac.uk
Paola Piccini	Member	Imperial College London, London UK	paola.piccini@imperial.ac.uk
Henrik Zetterberg	Member	University College London, London, UK / University of Gothenburg, Mölndal, Sweden	h.zetterberg@ucl.ac.uk
Alastair Noyce	Member	Queen Mary University of London, London UK	a.noyce@qmul.ac.uk
Michael Lawton	Member	University of Bristol, Bristol, UK	Michael.Lawton@bristol.ac.uk
Ashwani Jha	Member	University College London, London, UK	ashwani.jha@ucl.ac.uk
Brook Huxford	Member	Queen Mary University of London, London, UK	b.f.r.huxford@qmul.ac.uk
Shlomi Haar Millo	Member	Imperial College London, London, UK	s.haar@imperial.ac.uk
K. Ray Chaudhuri	Member	King's College London, London, UK	ray.chaudhuri@kcl.ac.uk
Carroll Siu	PPI	Expert by Experience	carrollsiu@gmail.com
Michèle Bartlett	PPI	Expert by Experience	hello@michelebartlett.co.uk
Kuhan Pushparatnam	PPI	Expert by Experience	Kuhan4@gmail.com
Daniel van Wamelen	ECR	King's College London, London, UK	daniel.van_wamelen@kcl.ac.uk
Anthony HV Schapira	Co-Chair	University College London, London, UK	a.schapira@ucl.ac.uk
Oliver Bandmann	Co-Chair	University of Sheffield, Sheffield, UK	o.bandmann@sheffield.ac.uk
Simon Stott	Member	Cure Parkinson's, London, UK	simon@cureparkinsons.org.uk
George Tofaris	Member	University of Oxford, Oxford, UK	george.tofaris@ndcn.ox.ac.uk
Esther Sammler	Member	University of Dundee, Dundee, UK	e.m.sammler@dundee.ac.uk
Heather Mortiboys	Member	University of Sheffield, Sheffield, UK	h.mortiboys@sheffield.ac.uk
Li Wei	Member	University College London, London, UK	l.wei@ucl.ac.uk
Alan Wong	Member	Royal Free Hospital NHS Foundation Trust, London, UK	alan.wong@nhs.net
Susan Duty	Member	King's College London, London, UK	susan.duty@kcl.ac.uk

David Dexter	Member	Parkinson's UK, London, UK	ddexter@parkinsons.org.uk
Paula Scurfield	PPI	Expert by Experience	paula_scurfield@hotmail.com
Keith Martin	PPI	Expert by Experience	keith.martin@zen.co.uk
Edwin Jabbari	ECR	University College London, London, UK	e.jabbari@ucl.ac.uk
Stephen Mullin	Chair	University of Plymouth, Plymouth, UK	stephen.mullin@plymouth.ac.uk
Huw Morris	Member	University College London, London, UK	huwmorris@gmail.com
David Breen	Member	University of Edinburgh, Edinburgh, UK	David.Breen@nhslothian.scot.nhs.uk
Christian Lambert	Member	University College London, London, UK	christian.lambert@ucl.ac.uk
Prasad Korlipara	Member	University College London, London, UK	l.korlipara@ucl.ac.uk
Monty Silverdale	Member	University of Manchester, Manchester, UK	monty.silverdale@nca.nhs.uk
Kailash Bhatia	Member	University College London, London, UK	k.bhatia@ucl.ac.uk
Alison Yarnall	Member	Newcastle University, Newcastle, UK	alison.yarnall@newcastle.ac.uk
Raj Khengar	Member	University College London, London, UK	r.khengar@ucl.ac.uk
Helen Collins	Member	Nat National Institute of Health Research Clinical Research Network, UK	Helen.Collins@ouh.nhs.uk
Fleur Hudson	Member	MRC Clinical Trials Unit at UCL, London, UK.	f.hudson@ucl.ac.uk
Gareth Baxendale	Member	National Institute of Health Research Clinical Research Network, UK	gareth.baxendale@nihr.ac.uk
Rebecca Croucher	Member	National Institute of Health Research Clinical Research Network, UK	rebecca.croucher@nihr.ac.uk
Sandra Bartolomeu-Pires	Member	Southhampton NHS Foundation Trust, Southampton, UK	Sandra.Bartolomeu-Pires@uhs.nhs.uk
Jennifer Allison	Member	National Institute of Health Research Clinical Research Network, UK	jennifer.allison@nihr.ac.uk
Jodie Forbes	PPI	Expert by Experience	jodiefm@hotmail.com

Alex Edwards	Member	Parkinson’s UK, London, UK	aedwards@parkinsons.org.uk
Sheila Wonnacott	PPI	Expert by Experience	wonnacott69@gmail.com
Dilan Athauda	ECR	University College London, London, UK	d.athauda@ucl.ac.uk
Joy Duffen	Co-Chair	Cure Parkinson’s, London, UK	joy.duffen@cantab.net
Sonia Gandhi	Co-Chair	University College London, London, UK	sonia.gandhi@ucl.ac.uk
Emily Henderson	Member	University of Bristol, Bristol, UK	emily.henderson@bristol.ac.uk
Maryanne Graham	Member	University College London, London, UK	maryanne.graham@ucl.ac.uk
Shona Clegg	Member	Parkinson's UK, London, UK	sclegg@parkinsons.org.uk
Karen Matthews	Member	National Institute of Health Research Clinical Research Network, UK	karen.l.matthews@nihr.ac.uk
Vince Greaves	Member	University College London, London, UK	vince.greaves@nhs.net
Eric Deeson	PPI	Expert by Experience	ericdeeson@aol.com
Laurel Miller	PPI	Expert by Experience	laurelfmiller@gmail.com
Joel Handley	ECR	Salford Royal NHS Foundation Trust, UK	joelhandley@doctors.org.uk
David Dexter	Member	Parkinson’s UK, London, UK	ddexter@parkinsons.org.uk
Helen Matthews	Member	Cure Parkinson’s, London, UK	helen@cureparkinsons.org.uk
Kevin McFarthing	Chair	Expert by Experience	kevin.mcfarthing@googlemail.com
Amit Batla	Member	University College London, London, UK	a.batla@ucl.ac.uk
Nikul Bakshi	Member	Parkinson's UK, London, UK	nbakshi@parkinsons.org.uk
Emma Lane	Member	Cardiff University, Cardiff, UK	laneel@cardiff.ac.uk
Miriam Parry	Member	Kings College Hospital NHS Foundation Trust, London, UK	miriamparry@nhs.net
Natasha Ratcliffe	Member	COUCH Health, Manchester, UK	natasha@couchhealth.co
Cristina Gonzalez-Robles	Member	University College London, London, UK	cristina.robles@ucl.ac.uk
Marie-Louise Zeissler	Member	University of Plymouth, Plymouth, UK	marie-louise.zeissler@plymouth.ac.uk
Georgia Mills	Member	University College London, London, UK	georgia.mills@ucl.ac.uk

Romy Ellis-Doyle	Member	University College London, London, UK	r.ellis-doyle@ucl.ac.uk
Sally L Collins	Member	University of Plymouth, Plymouth, UK	sally.l.collins@plymouth.ac.uk
Rebecca Chapman	Member	University of Plymouth, Plymouth, UK	rebecca.chapman@plymouth.ac.uk
Jesse Cedarbaum	Advisory Group (AG) Chair	Yale University, Connecticut, USA	Dr.C@coclinsci.com
Anthony Lang	AG member	University of Toronto, Ontario, Canada	Anthony.Lang@uhnresearch.ca
Brian Fiske	AG member	Michael J. Fox Foundation, New York, USA	bfiske@michaeljfox.org
Richard Wyse	AG member	Cure Parkinson’s, London, UK	richard@cureparkinsons.org.uk
Mahesh Parmar	AG member	MRC Clinical Trials Unit at UCL, London, UK.	m.parmar@ucl.ac.uk
Adam Boxer	AG member	University of California, San Francisco, California, USA	adam.boxer@ucsf.edu
Denise Wilson	AG member	National Institute of Health Research Clinical Research Network, UK	denise.wilson@nihr.ac.uk
Jean Christophe Corvol	AG member	Pitié-Salpêtrière Hospital, Sorbonne University, Assistance Publique Hôpitaux de Paris, Brain and Spine Institute, Paris, France	jean-christophe.corvol@aphp.fr
Jennifer Harris	AG member	National Institute of Health Research Clinical Research Network, UK	JHarris@abpi.org.uk
